# Estimate of Burden and Direct Healthcare Cost of Infectious Waterborne Disease in the United States

**DOI:** 10.3201/eid2701.190676

**Published:** 2021-01

**Authors:** Sarah A. Collier, Li Deng, Elizabeth A. Adam, Katharine M. Benedict, Elizabeth M. Beshearse, Anna J. Blackstock, Beau B. Bruce, Gordana Derado, Chris Edens, Kathleen E. Fullerton, Julia W. Gargano, Aimee L. Geissler, Aron J. Hall, Arie H. Havelaar, Vincent R. Hill, Robert M. Hoekstra, Sujan C. Reddy, Elaine Scallan, Erin K. Stokes, Jonathan S. Yoder, Michael J. Beach

**Affiliations:** Centers for Disease Control and Prevention, Atlanta, Georgia, USA

**Keywords:** burden of disease, cost of illness, waterborne, *Campylobacter*, *Cryptosporidium*, *Giardia*, *Legionella*, nontuberculous mycobacteria, norovirus, otitis externa, *Pseudomonas*, *Salmonella*, Shiga toxin-producing *Escherichia coli*, *Shigella*, *Vibrio*, viruses, bacteria, United States

## Abstract

Provision of safe drinking water in the United States is a great public health achievement. However, new waterborne disease challenges have emerged (e.g., aging infrastructure, chlorine-tolerant and biofilm-related pathogens, increased recreational water use). Comprehensive estimates of the health burden for all water exposure routes (ingestion, contact, inhalation) and sources (drinking, recreational, environmental) are needed. We estimated total illnesses, emergency department (ED) visits, hospitalizations, deaths, and direct healthcare costs for 17 waterborne infectious diseases. About 7.15 million waterborne illnesses occur annually (95% credible interval [CrI] 3.88 million–12.0 million), results in 601,000 ED visits (95% CrI 364,000–866,000), 118,000 hospitalizations (95% CrI 86,800–150,000), and 6,630 deaths (95% CrI 4,520–8,870) and incurring US $3.33 billion (95% CrI 1.37 billion–8.77 billion) in direct healthcare costs. Otitis externa and norovirus infection were the most common illnesses. Most hospitalizations and deaths were caused by biofilm-associated pathogens (nontuberculous mycobacteria, *Pseudomonas*, *Legionella*), costing US $2.39 billion annually.

At the beginning of the 20th century, diseases commonly transmitted by water, such as cholera and typhoid, were major causes of death in the United States (*1*). Reliable provision of treated, safe drinking water dramatically reduced the burden of these diseases and has been recognized as one of the greatest public health achievements of the 20th century (*2*). Despite this achievement, waterborne disease in the United States persists (*3**–**5*).

In the United States, outbreaks associated with large public drinking water systems have sharply declined in the past 40 years (*3*,*6*), likely the result of improvements in regulation and operation. However, transmission of disease via drinking water systems still occurs, often attributable to aging infrastructure, operational challenges, and the private or unregulated water systems (e.g., private wells) that serve an estimated 43 million persons (*7*). At the same time, the complexity and scope of water use has increased; drinking, sanitation, hygiene, cooling, and heating needs are supported by 6 million miles of plumbing inside US buildings (i.e., premise plumbing) (*8*,*9*). Premise plumbing water quality can be compromised by long water residency times, reduced disinfectant levels, and inadequate hot water temperatures, creating environments where pathogens (e.g., nontuberculous mycobacteria [NTM], *Pseudomonas*, and *Legionella*) can amplify in biofilms (*10**)*. People can be exposed to these pathogens through contact, ingestion, or inhalation of aerosols (e.g., from showerheads, building cooling towers, or decorative fountains).

As leisure time has increased, swimming pools, waterparks, water playgrounds, and hot tubs have proliferated (*5*). These venues rely largely on chlorination as the major barrier against disease transmission. *Cryptosporidium* has emerged as the major cause of outbreaks associated with treated aquatic venues because it is extremely chlorine resistant and has a low infectious dose (*5*,*11*,*12*). Warmer oceans have led to *Vibrio*-associated wound infections farther north than previously documented (*13*).

Estimates of the overall burden of foodborne disease in the United States, including both known and unknown agents, have been useful in directing prevention activities and setting public health goals (*14*,*15*). Quantifying the burden of infectious waterborne disease in the United States would also be beneficial.

Previous studies have attempted to estimate the burden of gastrointestinal illness (*16*,*17*) or all illness associated with drinking water (*18*) and untreated recreational water (*19*) in the United States, but the burden of disease from all water sources (drinking, recreational, environmental) and exposure routes (ingestion, contact, inhalation) has not been estimated. We present an estimate of the burden of waterborne disease in the United States that includes gastrointestinal, respiratory, and systemic disease; accounts for underdiagnosis; and includes all water sources and exposure routes.

## Methods

We defined waterborne disease as disease in which water was the proximate vehicle for exposure to an infectious pathogen. Thus, diseases such as Legionnaires’ disease (typically transmitted via inhaled water droplets containing *Legionella* bacteria) were considered waterborne. In contrast, arboviral diseases like malaria, for which standing water can increase the population of mosquitoes that transmit the parasite that causes malaria, were not considered waterborne. Algal toxins and chemical exposures were not considered. We determined the proportion of disease totals that were attributed to domestic waterborne exposure.

For this estimate, we chose diseases for which surveillance data, administrative data, or literature reports indicated that waterborne transmission for the disease in the United States was plausible, the disease was likely to cause substantial illness or death, and data were available to quantify associated health outcomes. Diseases included in this analysis were campylobacteriosis, cryptosporidiosis, giardiasis, Legionnaires’ disease, NTM infection, norovirus infection, acute otitis externa, *Pseudomonas* pneumonia and septicemia, Shiga toxin–producing *Escherichia coli* (STEC) infection serotype O157, non-O157 serotype STEC infection, salmonellosis, shigellosis, and vibriosis (including infection by *Vibrio alginolyticus*, *V. parahaemolyticus*, *V. vulnificus*, and other species). To aid in quantifying the burden of respiratory diseases and enteric disease separately, we considered Legionnaires’ disease, NTM infection, and *Pseudomonas* pneumonia primarily respiratory diseases, whereas we considered campylobacteriosis, cryptosporidiosis, giardiasis, norovirus infection, salmonellosis, and shigellosis primarily enteric diseases.

We employed methods similar to those of Scallan et al. (*14*,*15*) to estimate the number of illnesses, treat-and-release emergency department (ED) visits (i.e., visits in which the person was not admitted to the hospital), hospitalizations, and deaths attributed to waterborne transmission in the United States. We also quantified the direct healthcare costs of treat-and-release ED visits and hospitalizations, as measured by insurer and out-of-pocket payments. Our overall methods are described here; detailed methods are described in Appendix 1,2,3.

Data were for 2000–2015. All estimates were based on the 2014 US population (318.6 million persons); 2014 was the most recent year for which data were available for all surveillance sources. Estimates were derived from statistical models; each model input had uncertainty represented by a distribution of plausible values. Inputs are described in Appendix 1 and more details on the modeling process are described in Appendix 2. All estimates were rounded to 3 significant figures.

### Illnesses

The initial model input was the number of reported or documented cases of illness for each disease, selected hierarchically: data from active surveillance systems were preferred, passive surveillance data were used if active surveillance data were not available, and administrative data were used if no active or passive surveillance system for the disease existed (Table 1). Administrative data sources included the Health Care Utilization Project (HCUP) National Inpatient Sample (HCUP NIS) hospitalization database, the HCUP National Emergency Department Sample (HCUP NEDS) ED visit database, and, in the case of otitis externa, the National Ambulatory Medical Care Survey (NAMCS), which surveys visits to physicians’ offices. These administrative data sources use complex sample survey weighting methods and are considered nationally representative. We multiplied the initial reported or documented number of cases for each disease by a series of multipliers that accounted for underreporting and underdiagnosis (including illness severity, medical care-seeking, likelihood of specimen submission, proportion of laboratories capable of performing a diagnostic test, and test sensitivity).

**Table 1:** Data sources used to estimate the total number of illnesses for selected infectious diseases, United States*

Active surveillance data (name of surveillance system)	Passive surveillance data	Administrative data
Campylobacteriosis (FoodNet)	Giardiasis (NNDSS)	NTM infection (HCUP NEDS/NIS)
Cryptosporidiosis (FoodNet)	Legionnaires’ disease (NNDSS)	Otitis externa (NAMCS, HCUP NEDS/NIS)
Norovirus (*20*,*21*)	*Vibrio* spp. infection (COVIS)	*Pseudomonas* pneumonia (HCUP NEDS/NIS)
Salmonellosis, nontyphoidal (FoodNet)	*Vibrio alginolyticus* infection (COVIS)	*Pseudomonas* septicemia (HCUP NEDS/NIS)
STEC infection, O157 (FoodNet)	*Vibrio parahaemolyticus* infection (COVIS)	
STEC infection, non-O157 (FoodNet)	*Vibrio vulnificus* infection (COVIS)	
Shigellosis (FoodNet)	Other *Vibrio* infection (COVIS)	

*COVIS, Cholera and Other Vibrio Illness Surveillance; FoodNet, Foodborne Diseases Active Surveillance Network; HCUP NEDS/NIS, Healthcare Cost and Utilization Project’s National Emergency Department Sample and National Inpatient Sample; NAMCS, National Ambulatory Medical Care Survey; NNDSS, National Notifiable Diseases Surveillance System; NTM, nontuberculous mycobacterial; STEC, Shiga toxin–producing *Escherichia coli*.

### Emergency Department Visits

The surveillance systems used do not tally treat-and-release ED visits but do capture the proportion of patients hospitalized with a given disease; we combined this proportion with the ratio of treat-and-release ED visits for each disease (reported in HCUP NEDS) to hospitalizations for that disease (in HCUP NIS) to calculate the estimated proportion of reported cases with an ED visit. Although not all patients who visited the ED would have been reported or received a diagnosis, they were assumed to be more likely to receive a diagnosis than patients without an ED visit. Instead of applying the higher underdiagnosis factor used for illness, we used an underdiagnosis factor with a modal value of 2, consistent with previous estimates, and supported by a recent analysis comparing the incidence of bacterial gastroenteritis captured in surveillance and hospital discharge data (*14*,*22*,*23*).

### Hospitalizations

We applied the proportion of patients hospitalized according to surveillance data to the estimated number of reported cases to calculate the estimated number of reported hospitalized patients. If surveillance data were not available, the number of hospitalizations reported in HCUP NIS for a particular disease was used. Hospitalized case-patients were assumed to be more likely to have received a diagnosis than nonhospitalized case-patients. Instead of applying the higher underdiagnosis factor used for illness, we used an underdiagnosis factor with a modal value of 2, consistent with previous estimates, and, for some bacterial enteric diseases, supported by recent work (*14*,*22*,*23*).

### Deaths

We applied the proportion of case-patients who died, as reported by surveillance data, to the estimated number of reported cases to calculate the estimated number of reported deaths. If surveillance data were not available, we used the method of Gargano et al. (*24*). In brief, we combined the number of in-hospital deaths for each disease reported in HCUP NIS with the number of out-of-hospital deaths reported in death certificate records. We assumed that patients who died were more likely have received a diagnosis than patients who did not die. Instead of applying the higher underdiagnosis factor used for illness, we used an underdiagnosis factor with a modal value of 2, consistent with previous estimates (*14*,*22*).

### Domestically Acquired Waterborne Disease

We used surveillance data, when available, to determine the proportion of persons with a given disease who traveled outside the United States during the incubation period. The remaining proportion of cases was considered domestically acquired. When this information was not available, we used literature estimates and expert consultation. We used recent attribution estimates for each disease (*25*; E.M. Beshearse, unpub. data), derived through structured expert judgment (SEJ), a formal process that answers questions for which data are sparse using expert opinions (*26*,*27*), to determine the proportion of disease attributable to waterborne transmission.

### Uncertainty Estimates

For each input and multiplier in the model, we used a distribution that accounted for low, high, and midpoint estimates. This distribution accounted for the uncertainty in each input and multiplier and facilitated calculation of uncertainty intervals for final estimates. For diseases with surveillance data available, we used the methods of Scallan et al. to produce model inputs (*14*). For diseases with administrative data only (e.g., NTM infection and *Pseudomonas* pneumonia and septicemia), we used the mean hospitalization count from HCUP NIS and computed the illness count as the ratio of hospitalization count to hospitalization rate. We assumed the distribution of the hospitalization count to be normal, with the SD calculated from the reported 95% CI. As we did with surveillance data, we included the variation of hospitalization count over time in the model and assumed that the distribution for each multiplier followed the 4-parameter Program Evaluation and Review Technique (PERT) distribution (*28*), with disease-specific parameter values based on available publications.

Uncertainty in the final estimates is a cumulative effect of the uncertainty of each model input. Each multiplier was generated independently. Using 100,000 iterations, we obtained distributions of counts and used them to generate point estimates of means and the corresponding 95% credible interval (CrI, the 2.5th percentile through the 97.5th percentile of the empirical distribution). We generated all-disease totals for each outcome by sampling from the distributions generated for each individual disease, using SAS 9.4 (https://www.sas.com) and R 3.5.1 (*29*).

### Direct Healthcare Cost per ED Visit and Hospitalization

We used methods described previously (*30*,*31*) to calculate the direct cost of healthcare for ED visits and hospitalizations, using the 2012–2013 MarketScan research databases (IBM Watson Health, https://www.ibm.com/watson-health). These databases contain de-identified insurance billing data for tens of millions of persons covered by private, Medicare (which covers primarily persons >65 years of age), and Medicaid (which covers primarily persons with low incomes or disabilities) health insurance plans and contain information on insurance and out-of-pocket payments for hospitalizations, ED visits, doctors’ office visits, laboratory testing, and outpatient drug prescriptions. We used these data to calculate the sum of insurer and out-of-pocket payments per hospitalization or visit, by insurance source. We calculated a weighted cost per hospitalization or visit by multiplying the mean total payments for each insurance source by the proportion of cases with the insurance source in HCUP NIS or HCUP NEDS. We assumed that persons with other sources of health insurance (e.g., Tricare, the US military health insurance plan) or no health insurance have the same costs as persons with private insurance. For ED visit costs, we used the data described by Adam et al. (*30*), except for norovirus infection (not examined by Adam et al.) and STEC O157 and non-O157 (categorized differently by Adam et al.) (Appendix 1).

### Total Direct Health Care Costs of Domestically Acquired Waterborne Hospitalizations and ED Visits

We estimated the total direct healthcare cost of ED visits and hospitalizations attributed to waterborne transmission in the United States using the total number of ED visits and hospitalizations attributed to waterborne transmission in the United States. We multiplied these figures by the weighted average cost per ED visit or hospitalization, using 100,000 iterations, with uncertainty distributions as described (Appendix 1).

## Results

### Illnesses

We estimate that 33,600,000 (95% CrI 23,500,000–48,000,000) illnesses from the diseases in this analysis occurred in 2014, and of those, 7,150,000 (95% CrI 3,880,000–12,000,000; 21.3%) were attributed to waterborne transmission in the United States (Table 2). The diseases that caused the greatest number of domestically acquired waterborne illnesses were otitis externa (4,670,000 illnesses; 95% CrI 2,350,000–7,290,000) and norovirus infection (1,330,000 illnesses; 95% Cr 5,310–5,510,000), followed by giardiasis (415,000 illnesses; 95% CrI 140,000–816,000) and cryptosporidiosis (322,000 illnesses; 95% CrI 61,700–993,000). An estimated 96,000 domestically acquired waterborne respiratory illnesses occurred, and 2,330,000 domestically acquired waterborne enteric illnesses occurred.

**Table 2:** Estimated number of total cases of domestically acquired waterborne illness in 2014 for selected infectious diseases, United States*

Disease or syndrome	Estimated confirmed cases	Multipliers		Estimated total cases (95% CrI)	International travel, %	Waterborne, % (95% CrI)	Domestically acquired waterborne, no. (95% CrI)
		Under-reporting	Under-diagnosis				
Campylobacteriosis	54,000	1.0	28.3	1,540,000 (597,000–3,250,000)	14.4	13 (1–31)	171,000 (13,900–586,000)
Cryptosporidiosis	8,450	1.0	97.3	823,000 (243,000–2,160,000)	9.9	43 (17–73)	322,000 (61,700–993,000)
Giardiasis	17,900	1.30	45.9	1,070,000 (727,000–1,560,000)	12.3	44 (16–78)	415,000 (140,000–816,000)
Legionnaires’ disease	5,030	1.0	2.3	11,400 (8,920–13,600)	1.0	97 (67–100)	11,000 (7,430–13,300)
NTM infection	25,700	1.0	3.8	97,000 (75,700–122,000)	1.0	72 (39–94)	68,900 (35,800–100,000)
Norovirus	NA	1.0	NA	21,800,000 (12,100,000–36,000,000)	1.1	6 (0–25)	1,330,000 (5,310–5,510,000)
Otitis externa†	1,720,000	1.0	3.4	5,980,000 (3,200,000–8,880,000)	1.3	79 (67–95)†	4,670,000 (2,350,000–7,290,000)
*Pseudomonas* pneumonia	15,800	1.0	2.0	31,700 (19,300–46,000)	1.0	51 (14–80)	15,900 (4,240–29,000)
*Pseudomonas* septicemia	13,000	1.0	2.0	26,100 (16,700–35,900)	1.0	22 (3–53)	5,760 (743–14,400)
Salmonellosis, nontyphoidal	46,400	1.0	29.1	1,350,000 (733,000–2,450,000)	9.7	6 (0–22)	77,000 (5,640–277,000)
STEC infection, serotype O157	3,530	1.0	18.2	64,200 (13,000–188,000)	4.0	5 (1–13)	3,360 (336–12,900)
STEC infection, serotype non-O157	4,550	1.0	48.1	219,000 (80,000–493,000)	15.3	6 (0–17)	11,400 (0–43,900)
Shigellosis	13,600	1.0	33.1	449,000 (97,800–1,350,000)	7.8	4 (1–21)	17,300 (1,080–77,500)
*Vibrio* spp. infection	1,230	NA	NA	172,000 (126,000–231,000)	NA	NA	34,600 (17,600–56,900)
*V. alginolyticus*	234	1.1	142.8	36,700 (23,600–54,800)	6.5	37 (13–71)	12,700 (4,100–25,400)
*V. parahaemolyticus*	593	1.1	141.6	92,400 (55,000–144,000)	6.7	24 (7–38)	20,800 (6,000–39,000)
*V. vulnificus*	133	1.1	1.7	249 (178–340)	1.5	77 (40–91)	188 (93–277)
Other *Vibrio*	271	1.1	142.8	42,600 (25,500–66,500)	14.4	2 (0–23)	879 (3–8,490)
Total illness	NA	NA	NA	33,600,000 (23,500,000–48,000,000)	NA	NA	7,150,000 (3,880,000–12,000,000)

*Estimates rounded to 3 significant figures. CrI, credible interval; NA, not applicable; NTM, nontuberculous mycobacteria; STEC, Shiga toxin–producing *Escherichia coli*. †Combines the waterborne source attribution (*25*) for *Pseudomonas* spp. otitis externa (81%) and *Staphylococcus aureus *(75%) in a ratio of 2:1. More details provided in Appendix 1 (https://wwwnc.cdc.gov/EID/article/27/1/19-0676-App1.pdf).

### Emergency Department Visits

An estimated 601,000 (95% CrI 364,000–866,000) treat-and-release emergency department visits for the included diseases were attributed to waterborne transmission in the United States in 2014 (Table 3). Otitis externa caused the largest number of visits (567,000; 95% CrI 337,000–823,000).

**Table 3:** Estimated number of treat-and-release emergency department visits, hospitalizations, and deaths from domestically acquired waterborne transmission in 2014 for selected infectious diseases, United States*

Disease or syndrome	Treat-and-release ED visits†			Hospitalizations				Deaths		
	Total visits (95% CrI)	Domestic waterborne visits (95% CrI)		% Admitted to hospital	Total stays (95% CrI)	Domestic waterborne stays (95% CrI)		% Deaths	Total deaths (95% CrI)	Domestic waterborne deaths (95% CrI)
Campylobacteriosis	2,900 (1,620–4,630)	319 (31–966)		19.5	19,300 (8,790–34,900)	2,150 (192–6,900)		0.2	242 (0–1,150)	27 (0–146)
Cryptosporidiosis	1,260 (742–1,880)	492 (167–957)		19.2	2,870 (439–8,060)	1,120 (102–3,550)		0.3	61 (0–320)	24 (0–136)
Giardiasis	1,460 (902–2,090)	567 (185–1,120)		7.9	2,830 (1,760–4,070)	1,100 (364–2,180)		<0.1	4 (0–11)	1 (0–5)
Legionnaires’ disease	691 (316–1,220)	667 (289–1,200)		98.1	11,200 (8,750–13,300)	10,800 (7,280–13,100)		9.0	1,030 (762–1,330)	995 (655–1,310)
NTM infection	7,150 (5,110–9,620)	5,080 (2,560–7,750)		74.8	72,400 (57,300–89,700)	51,400 (26,800–74,100)		5.5	5,350 (4,020–6,920)	3,800 (1,950–5,620)
Norovirus	429,000‡ (318,000–605,000)	26,300‡ (105–106,000)		0.4	78,100 (58,500–104,000)	4,780 (19–19,300)		<0.1	885 (742–1,120)	54 (0–219)
Otitis externa	726,000 (466,000–994,000)	567,000 (337,000–823,000)		0.9	29,700 (19,200–40,600)	23,200 (13,900–33,600)		<0.1	280 (144–452)	219 (107–367)
*Pseudomonas* pneumonia	580 (321–902)	291 (75–552)		97.2	30,800 (18,700–44,700)	15,500 (4,130–28,100)		4.6	1,450 (786–2,420)	730 (185–1,460)
*Pseudomonas* septicemia	164 (36–326)	36 (2–106)		97.2	25,300 (16,300–34,800)	5,590 (722–14,000)		12.1	3,140 (1,990–4,430)	695 (89–1,740)
Salmonellosis, nontyphoidal	3,410 (2,100–4,900)	194 (15–671)		28.4	26,600 (11,400–52,800)	1,520 (100–5,660)		0.5	421 (0–1,140)	24 (0–103)
STEC infection, serotype O157	252 (92–465)	12 (2–35)		38.5	2,640 (487–7,630)	138 (14–503)		0.7	36 (0–314)	2 (0–17)
STEC infection, serotype non-O157	75 (12–171)	4 (0–16)		16.0	1,420 (264–3,810)	74 (0–308)		0.2	16 (0–184)	1 (0–12)
Shigellosis	1,650 (540–2,870)	64 (5–311)		24.4	6,380 (929–20,300)	245 (12–1,140)		0.1	26 (0–218)	1 (0–9)
*Vibrio* spp. infection	366 (122–700)	76 (14–166)		NA	782 (567–1,030)	251 (153–362)		NA	113 (67–156)	60 (27–92)
* V. alginolyticus*	NA§	NA§		15.9	74 (38–141)	26 (8–58)		0.8	4 (0–11)	1 (0–5)
* V. parahaemolyticus*	NA§	NA§		22.3	264 (136–410)	60 (16–112)		1.4	16 (7–32)	4 (1–9)
* V. vulnificus*	NA§	NA^‡^		85.4	213 (147–297)	161 (79–241)		28.8	72 (38–104)	54 (24–85)
Other *Vibrio*	NA§	NA§		42.5	231 (134–350)	5 (0–46)		3.8	20 (11–33)	0 (0–4)
Total	1,180,000 (877,000–1,490,000)	601,000 (364,000–866,000)		NA	310,000 (263,000–360,000)	118,000 (86,800–150,000)		NA	13,100 (10,600–15,900)	6,630 (4,520–8,870)

*Estimates rounded to 3 significant figures. CrI, credible interval; ED, emergency department; NA, not applicable; NTM, nontuberculous mycobacterial; STEC, Shiga toxin–producing *Escherichia coli*. †Treat-and-release ED visits were defined as visits in which the person was not admitted to the hospital. ‡For norovirus infection only, ED visits in which the person was admitted to the hospital were included, for consistency with previous published estimates. §No International Classification of Diseases, 9th Revision, Clinical Modification, codes are available for *Vibrio* spp. infections, only a general code for “Vibriosis and cholera.” ED visit estimates relied on administrative data that used these codes, and thus are presented only for *Vibrio* infection overall.

### Hospitalizations

We estimate that these diseases were responsible for 118,000 (95% CrI 86,800–150,000) hospitalizations attributed to waterborne transmission in the United States (Table 3). The diseases with the largest number of hospitalizations were NTM infection (51,400 hospitalizations; 95% CrI 26,800–74,100), otitis externa (23,200 hospitalizations; 95% CrI 13,900–33,600), and *Pseudomonas* pneumonia (15,500 hospitalizations; 95% CrI 4,130–28,100). An estimated 77,700 respiratory hospitalizations were attributed to waterborne transmission, and 10,900 enteric hospitalizations were attributed to waterborne transmission.

### Deaths

The diseases examined in this analysis were responsible for 6,630 deaths (95% CrI 4,520–8,870) attributed to waterborne transmission in the United States in 2014 (Table 3). The diseases with the largest number of deaths attributed to waterborne transmission in the United States were NTM infection (3,800, 95% CrI 1,950–5,620), Legionnaires’ disease (995, 95% CrI 655–1,310), and *Pseudomonas* pneumonia (730, 95% CrI 185–1,460). An estimated 5,530 deaths from respiratory disease were attributed to waterborne transmission (83% of all domestically acquired waterborne deaths), and 131 deaths from enteric diseases were attributed to waterborne transmission.

### Direct Healthcare Costs of ED Visits and Hospitalizations 

*Pseudomonas* septicemia had the highest cost per hospital stay ($38,200; 95% CrI $6,340–$172,000), followed by Legionnaires’ disease ($37,300, CrI $7,950–$149,000) (Table 4). Payments for ED visits and hospitalizations attributed to waterborne transmission in the United States totaled US $3.33 billion (95% CrI $1.37–$8.77 billion) in 2014 dollars (Table 5). This amount included $1.33 billion (95% CrI $361 million–$4.44 billion) in commercial insurer payments, $1.52 billion (95% CrI $338 million–$5.84 billion) in Medicare payments, and $284 million (95% CrI $62.7 million–$906 million) in Medicaid payments (Appendix 3 Tables 1–3). The costliest diseases were NTM infection ($1.53 billion; 95% CrI $272 million–$6.38 billion), otitis externa ($564 million; 95% CrI $187 million–$1.57 billion), and *Pseudomonas* pneumonia ($453 million; 95% CrI $49.9 million–$1.95 billion). An estimated $2.39 billion in direct healthcare costs from domestically acquired waterborne respiratory disease were incurred (72% of all costs from domestically acquired waterborne disease), as were $160 million in direct healthcare costs from domestically acquired waterborne enteric diseases.

**Table 4:** Cost per hospital stay for selected diseases that can be transmitted by water, 2012–2013 IBM MarketScan health insurance databases, United States*

Disease/syndrome	Cost in 2014 US dollars (95% CrI)			
	Commercial insurance	Medicare	Medicaid	Overall
Campylobacteriosis	15,200 (1,520–47,100)	15,100 (1,630–55,300)	5,900 (85–29,000)	13,600 (3,850–35,800)
Cryptosporidiosis	17,900 (1,560–82,700)	17,300 (1,800–79,400)	10,700 (22–64,200)	16,100 (4,360–55,400)
Giardiasis	25,300 (1,790–168,000)	22,300 (1,890–96,900)	14,300 (159–88,000)	21,800 (6,160–99,200)
Legionnaires’ disease	45,900 (2,320–306,000)	33,600 (4,210–183,000)	18,700 (17–99,300)	37,100 (7,950–149,000)
NTM infection	44,100 (1,650–244,000)	27,600 (1,720–152,000)	14,800 (49–69,100)	29,600 (6,350–120,000)
Norovirus infection†				6,080
Otitis externa	13,800 (1,480–56,500)	14,400 (1,490–65,100)	6,680 (43–36,900)	12,200 (3,320–42,400)
*Pseudomonas* pneumonia	45,100 (1,510–193,000)	28,200 (1,890–146,000)	11,600 (18–53,200)	29,300 (5,910–114,000)
*Pseudomonas* septicemia	63,600 (1,450–386,000)	34,400 (2,200–181,000)	19,800 (47–113,000)	38,200 (6,340–172,000)
Salmonellosis, nontyphoidal	17,200 (2,010–73,600)	17,100 (1,400–62,700)	6,940 (70–26,300)	14,900 (4,300–46,900)
STEC infection, serotype O157	25,900 (2,410–150,000)	17,200 (1,860–82,200)	4,530 (3–30,200)	19,000 (3,790–85,000)
STEC infection, serotype non-O157	23,600 (1,390–95,700)	31,900 (2,620–250,000)	5,020 (458–32,000)	24,200 (4,780–138,000)
Shigellosis	19,000 (2,910–85,300)	13,500 (1,610–39,600)	7,710 (37–51,300)	14,200 (4,130–48,000)
*Vibrio* spp. infection	17,400 (2,260–50,500)	18,400 (0,977–78,700)	4,600 (13–46,000)	16,000 (3,780–39,900)

*Estimates rounded to 3 significant figures. Overall cost calculated using the sum of insurer and out-of-pocket payments per stay for each payment source multiplied by the proportion of persons in the Health Care Utilization Project’s Nationwide Inpatient Sample with each payment source, for the corresponding disease or syndrome. This produces a weighted average cost per stay that reflects the differing proportion of payment sources for each disease or syndrome. Persons who had a payment source other than commercial insurance, Medicare, or Medicaid (i.e., persons covered by Tricare (the healthcare plan for persons affiliated with the US armed services, who were uninsured, or who had an unknown source of insurance) were assumed to have a cost per stay equivalent to the commercial insurance cost per stay. NTM, nontuberculous mycobacterial; STEC, Shiga toxin–producing *Escherichia coli*. †Norovirus costs were derived from previously published estimates that did not specify cost per insurance source or include uncertainty intervals.

**Table 5:** Total direct healthcare cost of ED visits and hospitalizations from domestically acquired waterborne transmission of selected infectious diseases, United States, 2014*

Disease or syndrome	Value (95% CrI)							
	Treat-and-release ED visits†				Hospitalization			Direct healthcare cost, millions
	Cost per visit	Total no. visits	Total cost, millions		Cost per stay	Total no. hospital stays	Total cost, millions	
Campylobacteriosis	1,710 (137–5,810)	319 (31–966)	0.545 (0.0177–2.61)		13,600 (3,850–35,800)	2,150 (192–6,900)	30.0 (1.71–121)	30.5 (2.10–121)
Cryptosporidiosis	1,960 (238–6,270)	492 (167–957)	0.963 (0.0802–3.44)		16,100 (4,360–55,400)	1,120 (102–3,550)	17.9 (1.10–79.5)	18.9 (1.82–80.4)
Giardiasis	1,620 (196–7,510)	567 (185–1,120)	0.917 (0.0861–3.78)		21,800 (6,160–99,200)	1,100 (364–2,180)	23.9 (3.53–104)	24.8 (4.21–105)
Legionnaires’ disease	691 (288–1,390)	667 (289–1,200)	0.460 (0.127–1.13)		37,100 (7,950–149,000)	10,800 (7,280–13,100)	401 (79.0–1,690)	402 (79.5–1,690)
NTM infection	1,610 (129–6,430)	5,080 (2,560–7,750)	8.17 (0.584–34.0)		29,600 (6,350–120,000)	51,400 (26,800–74,100)	1,520 (266–6,370)	1,530 (272–6,380)
Norovirus‡	1,140	26,300	30.1		6,080	4,780	29	59.1
Otitis externa	494 (120–1,430)	567,000 (337,000–823,000)	280 (60.2–846)		12,200 (3,320–42,400)	23,200 (13,900–33,600)	285 (67.8–1,040)	564 (187–1,570)
*Pseudomonas* pneumonia	856 (89–4,190)	291 (75–552)	0.249 (0.0162–1.27)		29,300 (5,910–114,000)	15,500 (4,130–28,100)	452 (49.8–1,950)	453 (49.9– 1,950)
*Pseudomonas* septicemia	923 (95–3,190)	36 (2–106)	0.0334 (0.000716–0.186)		38,200 (6,340–172,000)	5,590 (722–14,000)	214 (11.4–1,030)	214 (11.4–1,030)
Salmonellosis, nontyphoidal	1,230 (161–4,500)	194 (15–671)	0.240 (0.00734–1.24)		14,900 (4,300–46,900)	1,520 (100–5,660)	22.6 (0.870–110)	22.8 (1.08–110)
STEC infection, serotype O157	1,070 (109–2,350)	12 (2–35)	0.0130 (0.00734–0.051)		19,000 (3,790–85,000)	138 (14–503)	2.67 (0.129–14.5)	2.68 (0.141–14.5)
STEC infection, serotype non-O157	1,070 (109–2,350)	4 (0–16)	0.00440 (0–0.0223)		24,200 (4,780–138,000)	74 (0–308)	1.76 (0–11.0)	1.76 (0.00186–11.0)
Shigellosis	952 (115–3,980)	64 (5–311)	0.0609 (0.00171–0.349)		14,200 (4,130–48,000)	245 (13–1,140)	3.41 (0.106–18.9)	3.47 (0.140–19.0)
*Vibrio* spp. infection	1,030 (293–3,330)	76 (14–166)	0.0777 (0.00765–0.276)		16,000 (3,780–39,900)	251 (153–362)	4.02 (0.811–10.7)	4.10 (0.891–10.8)
Total cost			322 (100–889)				3,010 (1,120–8,410)	3,330 (1,370–8,770)

*Values are 2004 US dollars except as indicated. Estimates rounded to 3 significant figures. CrI, credible interval; ED, emergency department; NTM, nontuberculous mycobacterial; STEC, Shiga toxin-producing *E. coli*. †Treat-and-release ED visits were defined as visits in which the person was not admitted to the hospital. ‡For norovirus only, costs were derived from previously published estimates that did not include uncertainty intervals. In addition, the number of ED visits includes visits in which the patient was admitted to the hospital.

## Discussion

Domestic waterborne transmission of 17 diseases in the United States caused ≈7.15 million (95% CrI 3.88–12.0 million) waterborne illnesses to occur annually during the study period, including 601,000 ED visits (95% CrI 364,000–866,000), 118,000 hospitalizations (95% CrI 86,800–150,000), and 6,630 deaths (95% CrI 4,520–8,870), and incurred $3.33 billion (95% CrI $1.31–$8.71 billion) in hospitalization and ED visit costs. This estimate includes drinking, recreational, and environmental water exposures. Although the risk of illness from enteric pathogens readily controlled by water treatment processes still exists, this analysis highlights the expanding role of environmental pathogens (e.g., mycobacteria, *Pseudomonas*, *Legionella*) that can grow in drinking water distribution systems; plumbing in hospitals, homes, and other buildings; recreational water venues; and industrial water systems (e.g., cooling towers). This snapshot of waterborne disease transmission in the United States circa 2014 contrasts with historical waterborne disease transmission before the implementation of drinking water treatment and sanitation systems (e.g., cholera, typhoid fever, and other enteric pathogens) (*1**)*.

Few comparable waterborne disease burden estimates exist for the United States or other high-income countries. The World Health Organization (WHO) has estimated water, sanitation, and hygiene-related disease and injury (i.e., diarrhea, drowning, malnutrition) (*32*). WHO’s estimated 6,600 annual US deaths from nondiarrheal infectious diseases is within the range of our estimate, although the infectious diseases included were not specified, making direct comparison difficult. Work from Australia used the WHO estimates to calculate the waterborne burden of 5 enteric pathogens, whereas estimates from Canada assessed the burden of AGI from drinking water and the burden of 5 enteric pathogens from private wells and small water systems (*33**–**35*). Work in Europe estimated the proportion of 9 primarily enteric diseases attributable to water (*36**)*. Prior estimates of the burden of waterborne disease in the United States focused on the burden of gastrointestinal illness associated with drinking water and an estimated 4–32 million cases of illness each year (*16**–**18*). Our estimate differs from previous work because it focuses on specific pathogens, includes nongastrointestinal diseases, and considers all waterborne exposure routes.

A previous estimate of foodborne disease found fewer illness, hospitalizations, and deaths from foodborne disease due to known pathogens (*14*), although it found more illness when unspecified agents were considered (*15**)*. For pathogens included in both estimates, underdiagnosis multipliers did not differ substantially, except for decreases in STEC multipliers because of improved laboratory capacity. The higher totals in this analysis reflect the diseases selected for inclusion, some of which cause severe respiratory diseases more likely to result in hospitalization and death than the diseases with primarily enteric effects that were included in the foodborne estimate. When estimates for the enteric pathogens included in both analyses are compared, the waterborne burden is lower than the foodborne burden. This difference could be because drinking and treated recreational water systems were designed to prevent enteric illness, and the intervention (disinfection) is relatively simple compared with the manifold interventions needed to prevent foodborne illness.

This work is subject to several limitations. First, we used a series of multipliers to generate estimates of disease, and accuracy of these estimates relies on the accuracy of the multipliers. Although we attempted to account for the uncertainty of each data point using uncertainty intervals, any systematic errors in multipliers will produce a biased estimate. For example, waterborne transmission is not the sole route of transmission for any of the diseases in this work; many of the included diseases can be transmitted through multiple pathways (e.g., cryptosporidiosis can be waterborne, foodborne, or transmitted directly from animals or humans). We also relied on structured expert judgment (SEJ) to estimate the proportions of diseases attributed to waterborne transmission. SEJ is an approach used when primary data are not available, and is subject to limitations including expert bias (*26*,*27*). For norovirus infection, the uncertainty interval for the waterborne attribution percentage was large, reflecting a lack of consensus among experts, and resulting in an estimate of illness with a wide credibility interval (1,330,000 [95% CrI 5,310–5,510,000] illnesses). Second, this analysis is limited to 17 infectious diseases with adequate surveillance or administrative data available and does not include all disease associated with waterborne transmission in the United States. Insufficient data were available to quantify the contribution of many viral diseases, including sapovirus, rotavirus, and astrovirus; or free-living ameba infections, which cause deaths in the United States each year (*5*). Noninfectious diseases (e.g., from exposure to harmful algal blooms, heavy metals, disinfection byproducts) were not considered. Third, these estimates used administrative data and relied on coding from the International Classification of Diseases, 9th Revision, Clinical Modification, which might not accurately capture the actual disease of the ill person. Fourth, the cost estimates consider only out-of-pocket and insurer payments and do not account for the total amount of time or wages lost to ill health, disability, early death, or other indirect costs. Physicians’ office visits were not included, because data were not available. Payment totals might not reflect the actual cost incurred by healthcare providers. Fifth, this work did not make separate estimates for different age, demographic, or risk groups. Risks could differ by group (e.g., children swim more often and have higher rates of cryptosporidiosis), resulting in over- or underestimation of waterborne disease (*37*,*38*). Cost estimates did not consider the contribution of immunosuppressing conditions or other concurrent conditions to the healthcare costs incurred. Finally, some estimates used data from FoodNet. In 2007, Hispanic persons were underrepresented in FoodNet sites (*39*). Appendix 1 contains additional pathogen-specific limitations. Analytic strengths of these burden estimates include the use of active surveillance data when possible, estimates from a comprehensive structured expert judgment, and credible intervals to acknowledge the inherent uncertainty in the model inputs and outputs.

The data presented here reflect the changing picture of waterborne disease in the United States and underscore the role of environmental pathogens that grow in biofilms. An estimated 7.15 million (95% CrI 3.88 million–12.0 million) domestically acquired waterborne illnesses occur in the United States each year, highlighting the need to focus public health resources on the prevention and control of these diseases, including surveillance for the diseases in this estimate that do not have a dedicated national case surveillance system (e.g., NTM infections). These findings should serve as a foundation for improved disease surveillance, inform waterborne disease prevention priorities, and help measure progress in the prevention of waterborne disease in the United States.

Appendix 1 Estimation and uncertainty model inputs for study of the burden and direct healthcare cost of infectious waterborne disease in the United States.

Appendix 2 Model types used to make estimates for study of the burden and direct healthcare cost of infectious waterborne disease in the United States.

Appendix 3 Additional information on the burden and direct healthcare cost of infectious waterborne disease in the United States.
