# Supplementary material for: Estimate of Burden and Direct Healthcare Cost of Infectious Waterborne Disease in the United States
Source: Emerg Infect Dis. 2021 Jan;27(1):140–9. doi: 10.3201/eid2701.190676 (PMC7774540; doi:10.3201/eid2701.190676)
Supplement: Appendix 1 — Estimation and uncertainty model inputs for study of the burden and direct healthcare cost of infectious waterborne disease in the United States. [file 19-0676-Techapp-s1.pdf]

Article DOI: https://doi.org/10.3201/eid2701.190676

Estimate of Burden and Direct Healthcare Cost of Infectious Waterborne Disease in the United States

Appendix 1

Estimation and Uncertainty Model Inputs for Selected Diseases Transmitted through Water

Appendix 1 Table 1. Estimation and uncertainty model inputs for selected diseases transmitted through water, United States

Pathogen: *Campylobacter* spp.			
Model input	Data source(s)	Distribution*	Parameters
Reported/projected US illnesses	Number of illnesses caused by *Campylobacter* spp. infection reported to CDC's Foodborne Diseases Active Surveillance Network (FoodNet) by FoodNet site (n = 10) and year (2012–2015) (*1*) scaled up to the US population (the FoodNet catchment area covers 10 sites around the United States and represented 15.3% of the US population in the study time period.)	Empirical	By site and year (2012–2015), Appendix 1 Table 2
Population adjustment (year)	Incidence of *Campylobacter* infection in each FoodNet site by year applied to 2014 US Census population estimates (*2*).	Degenerate	Adjustment by year (2012–2015): 1.01, 1.0, 1.0, 0.99
Underreporting	No underreporting multiplier; we assumed that all laboratory-confirmed *Campylobacter* illnesses were enumerated by FoodNet active surveillance.		
Underdiagnosis (for number of illnesses)			
Proportion severe	Proportion of cases reporting bloody diarrhea from FoodNet surveillance of laboratory-confirmed *Campylobacter* infections (*3*). We used the same lower and upper endpoints derived from Scallan et al. (*3*).	PERT	Low, modal, high values: 0.36, 0.45, 0.52
Medical care seeking (severe)	Proportion (and 95% confidence interval [CI]) of survey respondents with bloody diarrhea who sought medical care, from FoodNet Population Surveys (2000–2001, 2002–2003, 2006–2007) (*3*).	PERT	Low, modal, high values: 0.19, 0.35, 0.51
Medical care seeking (mild)	Proportion (and 95% CI) of survey respondents with non-bloody diarrhea who sought medical care, from FoodNet Population Surveys (2000–2001, 2002–2003, 2006–2007) *(3)*.	PERT	Low, modal, high values: 0.15, 0.18, 0.20
Specimen submission (severe)	Proportion (and 95% CI) of survey respondents who submitted a fecal specimen among persons with bloody diarrhea who sought medical care, from FoodNet Population Surveys (2000–2001, 2002–2003, 2006–2007) (*3*).	PERT	Low, modal, high values: 0.11, 0.36, 0.62
Specimen submission (mild)	Proportion (and 95% CI) of survey respondents who submitted a fecal specimen among persons with non-bloody diarrhea who sought medical care, from FoodNet Population Surveys (2000–2001, 2002–2003, 2006–2007) (*3*).	PERT	Low, modal, high values: 0.12, 0.19, 0.25
Laboratory testing	Proportion of clinical laboratories routinely testing fecal samples for *Campylobacter*, from the FoodNet Laboratory Survey (*4*). Uncertainty with this proportion (97%) was based on a 50% relative increase/decrease from 0.97 on an odds scale.	PERT	Low, modal, high values: 0.94, 0.97, 1.00
Positive predictive value	Because a substantial proportion of *Campylobacter* cases in 2014 were diagnosed by culture-independent diagnostic test (CIDT) only (*5*), and CIDTs have a lower specificity than culture-based methods, it was necessary to account for possible false-positive results from CIDT-only cases. For reported cases that were confirmed by CIDT alone, we used the positive predictive value (PPV) to convert CIDT cases to culture-confirmed cases. The PPV was defined as the probability of having a positive result in a culture-based test given a positive CIDT test. Further, because the PPV of PCR-	PERT	PCR: Low, modal, high values: 0.80, 0.85, 0.90<br><br>Non-PCR: Low, modal, high values: 0.37, 0.52, 0.73

Pathogen: *Campylobacter* spp.			
Model input	Data source(s)	Distribution*	Parameters
	based tests differ from non-PCR CIDT methods, we used separate PPVs for PCR and non-PCR CIDTs. Cases based on CIDT tests only were grouped into PCR and non-PCR. PPVs were derived from a previous publication that used data from FoodNet sites (*6*). Once CIDT-only cases were adjusted using the PPV to convert CIDT cases to the equivalent number of culture-confirmed cases, they were added to the number of reported culture-confirmed cases to obtain the adjusted total number of culture-confirmed cases. The PPVs were assumed to follow the PERT distribution.		
Culture-based test sensitivity	We used a laboratory test sensitivity rate of 70% based on studies of *Salmonella* (*7,8*) for the equivalent number of culture-confirmed cases. We assumed a lower bound of 60% and an upper bound of 90%.	PERT	Low, modal, high values: 0.60, 0.70, 0.90
Proportion with treat-and-release ED visit	Proportion of treat-and-release emergency department (ED) visits (i.e., visits where the person was not admitted to the hospital) in the 2012–2014 Healthcare Cost and Utilization Project's National Emergency Department Sample (HCUP NEDS) for International Classification of Diseases, 9th Edition, Clinical Modification (ICD-9-CM) code 008.43 (campylobacteriosis) compared with hospitalizations in the 2012–2014 HCUP National Inpatient Sample (HCUP NIS) for ICD-9-CM code 008.43. This proportion was multiplied by the number of patients with FoodNet cases of *Campylobacter* infection who were hospitalized.	Empirical ratio	HCUP ED visits by year (2012–2014): 1,173, 1,636, 1,501<br>HCUP hospitalizations (2012–2014): 5,915, 6,515, 6,090<br>Proportion by year, 2012–2014: 0.20, 0.26, 0.25
Proportion hospitalized	Proportion of case-patients with FoodNet cases of *Campylobacter* infection who were hospitalized.	Empirical	By site and year (2012–2015); Table 3
Proportion who died	Proportion of case-patients with FoodNet cases of *Campylobacter* infection who died.	Empirical	By site and year (2012–2015); Table 4
Underdiagnosis (ED visits, hospitalizations, deaths)	Number of ED visits, hospitalizations, and deaths doubled to account for underdiagnosis.	PERT	Low, modal, high values: 1, 2, 3
Proportion travel-related	Proportion of case-patients with FoodNet cases of *Campylobacter* infection who reported travel outside the United States within 7 d of illness onset (2012–2015). Uncertainty with this proportion (15%) was based on a 50% relative increase/decrease on an odds scale.	PERT	Low, modal, high values: 0.10, 0.15, 0.21
Proportion waterborne	Structured expert judgement estimate for *Campylobacter* infection (*9*).	Empirical	2.5th percentile, median, mean, 97.5th percentile: 0.01, 0.11, 0.13, 0.31
Cost of treat-and-release ED visits	Sum of insurer and out-of-pocket payments for treat-and-release emergency department visits for ICD-9-CM code 008.43 (campylobacteriosis), in 2014 US dollars, in 2012–2013 IBM MarketScan research databases, as reported by Adam et al. (*10*).	Empirical	Mean (2.5th percentile, 97.5th percentile): 1,710 (137–5,810)
Cost of hospitalizations	Sum of insurer and out-of-pocket payments for hospitalizations for ICD-9-CM code 008.43 (campylobacteriosis), in 2014 US dollars, in 2012–2013 IBM MarketScan research databases	Empirical	Mean (2.5th percentile, 97.5th percentile): 13,600 (3,850–35,800)
Pathogen-specific limitations and discussion	Diagnostic testing for campylobacteriosis is changing rapidly and the proportion of reported cases diagnosed by culture-independent diagnostic test alone is increasing. The positive predictive value of CIDTs varies by method (PCR vs. non-PCR) and by brand. We attempted to account for variation by method but were unable to account for variation by brand. The proportion of laboratories routinely testing for *Campylobacter* is based on a survey conducted from 1995 to 2000. It is likely that laboratory testing practices have changed since 2000. However, after consultation with CDC enteric disease experts, updated data were not available, and it was agreed that 97% of laboratories routinely testing for *Campylobacter* was a conservative estimate (because the higher the proportion of laboratories routinely testing for a pathogen, the lower the underdiagnosis multiplier).		

Pathogen: *Cryptosporidium* spp.			
Model input	Data source(s)	Distribution	Parameters
Reported illnesses	Number of illnesses caused by *Cryptosporidium* spp. infection reported to CDC's Foodborne Diseases Active Surveillance Network (FoodNet) by FoodNet site (n = 10) and year (2012–2015) (*1*); scaled up to the US population (the FoodNet catchment area covers 10 sites around the United States and represented 15.3% of the US population in the study time period).	Empirical	By site and year (2012–2015); Appendix 1 Table 2
Population adjustment (year)	Incidence of *Cryptosporidium* spp. infection in each FoodNet site by year applied to 2014 US Census population estimates (*2*).	Degenerate	Adjustment by year (2012–2015): 1.01, 1.0, 1.0, 0.99

Pathogen: *Cryptosporidium* spp.			
Model input	Data source(s)	Distribution	Parameters
Underreporting	No underreporting multiplier; we assumed that all laboratory-confirmed *Cryptosporidium* spp. illnesses were enumerated by FoodNet active surveillance.	None	None
Underdiagnosis (for number of illnesses			
Percent severe	The proportion of laboratory-confirmed *Cryptosporidium* spp. cases reporting bloody diarrhea was assumed to be low.	PERT	Low, modal, high values: 0.0, 0.0, 0.05
Medical care seeking (severe)	Proportion (and 95% CI) of survey respondents with bloody diarrhea who sought medical care, from FoodNet Population Surveys (2000–2001, 2002–2003, 2006–2007) (*3*).	PERT	Low, modal, high values: 0.19, 0.35, 0.51
Medical care seeking (mild)	Proportion (and 95% CI) of survey respondents with non-bloody diarrhea who sought medical care, from FoodNet Population Surveys (2000–2001, 2002–2003, 2006–2007) (*3*).	PERT	Low, modal, high values: 0.15, 0.18, 0.20
Specimen submission (severe)	Proportion (and 95% CI) of survey respondents who submitted a fecal specimen among persons with bloody diarrhea who sought medical care, from FoodNet Population Surveys (2000–2001, 2002–2003, 2006–2007) (*3*).	PERT	Low, modal, high values: 0.11, 0.36, 0.62
Specimen submission (mild)	Proportion (and 95% CI) of survey respondents who submitted a fecal specimen among persons with non-bloody diarrhea who sought medical care, from FoodNet Population Surveys (2000–2001, 2002–2003, 2006–2007) (*3*).	PERT	Low, modal, high values: 0.12, 0.19, 0.25
Laboratory testing	Proportion of clinical laboratories routinely testing fecal samples for *Cryptosporidium* spp., from the FoodNet Laboratory Survey (*4*). Uncertainty with this proportion was based on a 50% relative increase/decrease on an odds scale.	PERT	Low, modal, high values: 0.27, 0.36, 0.46
Test sensitivity	Average from published studies (*3*). Uncertainty with this proportion (87%) was based on a 50% relative increase/decrease from 0.87 on an odds scale.	PERT	Low, modal, high values: 0.81, 0.87, 0.91
Proportion with treat-and-release ED visit	Proportion of treat-and-release ED visits (i.e., visits in which the person was not admitted to the hospital) in the 2012–2014 Healthcare Cost and Utilization Project's National Emergency Department Sample (HCUP NEDS) for International Classification of Diseases, 9th Edition, Clinical Modification (ICD-9-CM) code 007.4 (cryptosporidiosis) compared with hospitalizations in the 2012–2014 HCUP National Inpatient Sample (HCUP NIS) for ICD-9-CM code 007.4. This proportion was multiplied by the number of patients with FoodNet cases of *Cryptosporidium* spp. infection who were hospitalized.	Empirical ratio	HCUP ED visits by year (2012–2014): 604, 658, 610<br>HCUP hospitalizations (2012–2014): 5,915, 6,515, 6,090<br>Proportion by year, 2012–2014: 0.33, 0.36, 0.33
Proportion hospitalized	Proportion of case-patients with FoodNet cases of *Cryptosporidium* spp. infection who were hospitalized.	Empirical	By site and year (2012–2015); Appendix 1 Table 3
Proportion who died	Proportion of case-patients with FoodNet cases of *Cryptosporidium* spp. infection who died.	Empirical	By site and year (2012–2015); Appendix 1 Table 4
Underdiagnosis (ED visits, hospitalizations, deaths)	Number of emergency department visits, hospitalizations, and deaths doubled to account for underdiagnosis.	PERT	Low, modal, high values: 1, 2, 3
Proportion travel-related	Proportion of case-patients with FoodNet cases of *Cryptosporidium* spp. infection who reported travel outside the United States within 15 d of illness onset (2012–2015). Uncertainty with this proportion was based on a 50% relative increase/decrease on an odds scale.	PERT	Low, modal, high values: 0.067, 0.098, 0.138
Proportion waterborne	Structured expert judgement estimate for *Cryptosporidium* spp. infection (*9*).	Empirical	2.5th percentile, median, mean, 97.5th percentile: 0.17, 0.43, 0.43, 0.73
Cost of treat-and-release emergency visits	Sum of insurer and out-of-pocket payments for treat-and-release emergency department visits for ICD-9-CM code 007.4, in 2014 US dollars, in 2012–2013 IBM Watson Health MarketScan research databases, as reported by Adam et al. (*10*).	Empirical	Mean (2.5th percentile, 97.5th percentile): 1,960 (238–6,270)
Cost of hospitalizations	Sum of insurer and out-of-pocket payments for hospitalizations for ICD-9-CM code 007.4, in 2014 US dollars, in 2012–2013 IBM Watson Health MarketScan research databases.	Empirical	Mean (2.5th percentile, 97.5th percentile): 16,100 (4,360–55,400)
Pathogen-specific limitations and discussion	Testing methods for *Cryptosporidium*, a parasite, differ from culture-based bacterial methods. Immunochromatographic testing was likely the most common diagnostic testing method in the time span of this analysis. Specificity of immunochromatographic testing varies by brand and ranges from 67% to 100%. We did not account for false positives because of a lack of data on testing methods, brands, and whether follow-up testing was performed.		

Pathogen: *Giardia duodenalis*			
Model input	Data source(s)	Distribution	Parameters
Reported illnesses	Number of illnesses caused by *Giardia duodenalis* reported to CDC's National Notifiable Diseases Surveillance System (NNDSS) (2008–2015) (*11*). Because not all states report giardiasis to NNDSS, estimates were scaled up to the total US population.	Empirical	By year (2008–2015): 19,153, 19,562, 19,984, 16,870, 15,224, 15,318, 14,657, 14,678
Population adjustment (year)	Population ratios applied to each year from 2008–2014 based on US Census population estimates for states that report giardiasis to NNDSS (*2*)	Degenerate	Adjustment by year (2008–2014): 1.05, 1.04, 1.03, 1.02, 1.01, 1.0, 1.0
Underreporting	Passive surveillance multiplier used to adjust for underreporting (*3*)	PERT	Low, modal, high values: 1.0, 1.3, 1.6
Underdiagnosis (for number of illnesses)			
Percent severe	Assumed to be mostly mild (*12*).	PERT	Low, modal, high values: 0.0, 0.0, 0.05
Medical care seeking (severe)	Proportion (and 95% CI) of survey respondents with bloody diarrhea who sought medical care, from FoodNet Population Surveys (2000–2001, 2002–2003, 2006–2007) (*3*)	PERT	Low, modal, high values: 0.19, 0.35, 0.51
Medical care seeking (mild)	Proportion (and 95% CI) of survey respondents with non-bloody diarrhea who sought medical care, from FoodNet Population Surveys (2000–2001, 2002–2003, 2006–2007) (*3*).	PERT	Low, modal, high values: 0.15, 0.18, 0.20
Specimen submission (severe)	Proportion (and 95% CI) of survey respondents who submitted a fecal specimen among persons with bloody diarrhea who sought medical care, from FoodNet Population Surveys (2000–2001, 2002–2003, 2006–2007) (*3*).	PERT	Low, modal, high values: 0.11, 0.36, 0.62
Specimen submission (mild)	Proportion (and 95% CI) of survey respondents who submitted a fecal specimen among persons with non-bloody diarrhea who sought medical care, from FoodNet Population Surveys (2000–2001, 2002–2003, 2006–2007) (*3*).	PERT	Low, modal, high values: 0.12, 0.19, 0.25
Laboratory testing	Used the parameter generated by Scallan et al. (*3*), which was based on consultations with clinical and billing code experts. Uncertainty with this proportion (80%) was based on a 50% relative increase/decrease from 0.80 on an odds scale.	PERT	Low, modal, high values: 0.73, 0.80, 0.86
Test sensitivity	Average from published studies (*3*). We used uniform minimum variance unbiased (UMVU) estimators for lower and upper endpoints.	PERT	Low, modal, high values: 0.72, 0.83, 0.93
Proportion with treat-and-release ED visit	Proportion of treat-and-release ED visits (in which the person was not admitted to the hospital) in the 2012–2014 Healthcare Cost and Utilization Project's National Emergency Department Sample (HCUP NEDS) for International Classification of Diseases, 9th Edition, Clinical Modification (ICD-9-CM) code 007.1 (giardiasis) compared with hospitalizations in the 2012–2014 HCUP National Inpatient Sample (HCUP NIS) for ICD-9-CM code 007.1.	Empirical ratio	HCUP ED visits by year (2012–2014): 713, 712, 746<br>HCUP hospitalizations (2012–2014): 1,430, 1,425, 1,415<br>Proportion by year, 2012–2014: 0.5, 0.5, 0.53
Proportion hospitalized	Proportion of case-patients hospitalized, estimated using annual national estimates of hospitalization from the National Inpatient Sample (NIS) (2008–2014) using ICD-9-CM code 007.1 (giardiasis) compared with the number of illnesses reported in NNDSS (*11*).	Empirical	By year (2008–2014): 0.098, 0.094, 0.089, 0.094, 0.093, 0.097
Proportion who died	Proportion of case-patients who died, estimated using annual national estimates of in-hospital deaths from the NIS (2008–2015) using ICD-9-CM code 007.1 (giardiasis) compared with the total number of cases from NNDSS.	Empirical	Number of deaths by year (2008–2014): 2, 0, 1, 5, 3, 1, 2, 1<br>Proportion by year (per 100,000 cases): 10.4, 0, 5, 29.6, 19.7, 6.5, 13.6, 6.8
Underdiagnosis (ED visits, hospitalizations, deaths)	Number of ED visits, hospitalizations, and deaths doubled to account for underdiagnosis.	PERT	Low, modal, high values: 1, 2, 3
Proportion travel-related	12.1% based on a published study (*13*). Uncertainty with this proportion was based on a 50% relative increase/decrease on an odds scale	PERT	Low, modal, high values: 0.08, 0.12, 0.17
Proportion waterborne	Structured expert judgment estimate for giardiasis infection (*9*)	Empirical	2.5th percentile, median, mean, 97.5th percentile: 0.16, 0.43, 0.44, 0.78
Cost of treat-and-release emergency visits	Sum of insurer and out-of-pocket payments for treat-and-release emergency department visits for ICD-9-CM code 007.1, in 2014 US dollars, in 2012–2013 IBM Watson Health MarketScan research databases, as reported by Adam et al. (*10*)	Empirical	Mean (2.5th percentile, 97.5th percentile): 1,620 (196–7,510)
Cost of hospitalizations	Sum of insurer and out-of-pocket payments for hospitalizations for ICD-9-CM code 007.1, in 2014 US dollars, in 2012–2013 IBM Watson Health MarketScan research databases.	Empirical	Mean (2.5th percentile, 97.5th percentile): 21,800 (6,160–99,200)

Pathogen: *Giardia duodenalis*			
Model input	Data source(s)	Distribution	Parameters
Pathogen-specific limitations and discussion	Giardiasis is a nationally notifiable disease. However, each state has its own laws and regulations defining which diseases are reportable (https://wwwn.cdc.gov/nndss/data-collection.html). Clinical detection and diagnosis are challenging because many physicians lack familiarity with giardiasis, many symptoms (e.g., diarrhea) are nonspecific, and standard bacterial fecal cultures will not detect *Giardia* (*14–16*).		

Pathogen: *Legionella*			
Model input	Data source(s)	Distribution	Distribution values
Reported illnesses	Incidence of *Legionella* infection resulting in Legionnaires' disease reported to CDC's National Notifiable Diseases Surveillance System (NNDSS, 2008–2014 [*17*]).	Empirical	By year: 3181, 3522, 3346, 4202, 3688, 4954, 5166
Population adjustment (year)	Population ratios applied to each year from 2008–2014 based on US Census population estimates (*2*) and adjusted for increasing trend	Degenerate	Adjustment by year (2008–2014): 1.05, 1.04, 1.03, 1.02, 1.01, 1.0, 1.0
Underreporting	All cases assumed to be reported	Constant	100%
Percent severe	All cases of infection assumed to be severe	Constant	100%
Underdiagnosis (for number of illnesses)			
Medical care seeking	Assumed to have a high rate of medical care seeking (97.9% hospitalized in cases reported to CDC's Active Bacterial Core surveillance program, 2011–2015) (*18*).	PERT	Low, modal, high values: 0.99, 0.995, 1.0
Specimen submission	In one healthcare system where universal testing of patients with community-acquired pneumonia for Legionnaires' disease was implemented, 56% of patients with Legionnaires' disease would have been tested using standard guidelines (*19*).	PERT	Low, modal, high values: 0.46, 0.56, 0.66
Laboratory testing	We assumed that all facilities would have access to laboratories capable of performing the urinary antigen test for *Legionella pneumophila* serogroup 1.	Constant	100%
Laboratory test sensitivity	71% based on published study of sensitivity of urinary antigen test for all *Legionella* species and serogroups (*20*)	PERT	Low, modal, high values: 0.791, 0.794, 0.797
Proportion with a treat-and-release ED visit	Ratio of treat-and-release ED visits to hospitalizations from the Health Care Utilization Project's National Emergency Department Sample and National Inpatient Sample, 2012–2014, using ICD-9-CM code 482.84 (Legionnaires' disease). This proportion was multiplied by the number of case-patients with NNDSS cases of Legionnaires' disease who were hospitalized.	Empirical ratio	HCUP ED visits by year (2012–2014): 333, 445, 250<br>HCUP hospitalizations (2012–2014): 3,680, 4,810, 4,170<br>Proportion by year, 2012–2014: 0.09, 0.09, 0.06
Proportion hospitalized	Proportion hospitalized (97.9%) in cases reported to CDC's Active Bacterial Core surveillance program, 2011–2013 (*18*)	Empirical	By year (2008–2014): 0.981, 0.981, 0.981, 0.980, 0.976, 0.987, 0.980
Proportion who died	Proportion of case-patients who died, reported to CDC's Active Bacterial Core surveillance program, 2011–2015 (*18*)	Empirical	By year: 0.096, 0.1058, 0.0843, 0.0775
Underdiagnosis (ED visits, hospitalizations, deaths)	Because nearly all case-patients were hospitalized, the underdiagnosis multiplier for ED visits, hospitalizations, and deaths was assumed to be the same as the underdiagnosis multiplier for illnesses.	PERT	Low, modal, high values: 1.9, 2.3, 2.8
Proportion travel-related	Proportion of persons with Legionnaires' disease who reported travel outside the United States within 10 d of illness onset (2005–2014) in CDC's Supplemental Legionnaires' Disease Surveillance System (*14*). Uncertainty with this proportion (1%) was based on a 50% relative increase/decrease from 0.01 on an odds scale.	PERT	Low, modal, high values: 0.0067, 0.01, 0.0149
Proportion waterborne	Structured expert judgment estimate for Legionnaires' disease (*9*).	Empirical	2.5th percentile, median, mean, 97.5th percentile: 0.67, 1, 0.97, 1
Cost of treat-and-release ED visits	Sum of insurer and out-of-pocket payments for treat-and-release ED visits for ICD-9-CM code 482.84, in 2014 US dollars, in 2012–2013 IBM Watson Health MarketScan research databases, from data reported by Adam et al. (10).	Empirical	Mean (2.5th percentile, 97.5th percentile): 691 (288–1,390)
Cost of hospitalizations	Sum of insurer and out-of-pocket payments for hospitalizations for ICD-9-CM code 482.84, in 2014 US dollars, in 2012–2013 IBM Watson Health MarketScan research databases.	Empirical	Mean (2.5th percentile, 97.5th percentile): 37,100 (7,950–149,000)

Pathogen: *Legionella*			
Model input	Data source(s)	Distribution	Distribution values
Pathogen-specific limitations and discussion	Previously reported costs for treat-and-release ED visits did not report visits for Medicaid because of small sample size. Medicaid visit costs were included in this estimation. As a consequence, the weighted average cost per treat-and-release ED visits is lower that what was reported by Adam et al. (*10*).<br>In practice, Legionnaires' disease tends to be defined as a "severe" pneumonia, which is supported by the fact that nearly all reported case-patients have been hospitalized. Previous serologic studies, however, have shown that many persons not known to have a history of Legionnaires' disease have detectable titers of antibodies against *Legionella* (*21*). This indicates that less severe disease presentations may exist that have not been diagnosed or reported and would not be captured by this estimate.		

Pathogen: nontuberculous mycobacterial (NTM) infections			
Model input	Data source(s)	Distribution	Parameters
Reported hospitalizations	Number of case-patients hospitalized using annual national estimates from the National Inpatient Sample (NIS) (2012–2014) using International Classification of Diseases, 9th Edition, Clinical Modification (ICD-9-CM) code 031 (031.0, pulmonary NTM infection; 031.1, cutaneous NTM infection; 031.2, disseminated NTM infection; 031.8, other specified NTM disease; 031.9, unspecified diseases due to mycobacteria).	Mixture of normals	By year (2012–2014): 18,130, 19,415, 19,525
Population adjustment (year)	Population ratios applied to each year, 2012–2014, based on US Census population estimates (*2*)	Degenerate	Adjustment by year (2012–2014): 1.01, 1.0, 1.0
Underreporting	All cases with an NTM ICD-9-CM code in the hospitalization record were assumed to be reported to NIS.		
Underdiagnosis (illnesses, ED visits, hospitalizations, deaths)	Strollo et al. estimated that 27% of NTM cases had the ICD-9-CM code in the hospitalization record (*22*); 1/0.27 = 3.704. Uncertainty with this proportion was based on a 50% relative increase/decrease on an odds scale.	PERT	Low, modal, high values: 2.802, 3.704, 5.056
Proportion with a treat-and-release ED visit	Proportion of treat-and-release ED visits (in which the person was not admitted to the hospital) in the 2012–2014 Healthcare Cost and Utilization Project's National Emergency Department Sample (HCUP NEDS) for ICD-9-CM code 031 compared with hospitalizations in the 2012–2014 HCUP National Inpatient Sample (HCUP NIS) for ICD-9-CM code 031.	Empirical ratio	HCUP ED visits by year (2012–2014): 1,670, 1,846, 2,121<br>HCUP hospitalizations (2012–2014): 18,130, 19,415, 19,525<br>Proportion by year, 2012–2014: 0.09, 0.10, 0.11
Proportion hospitalized	After conferring with subject matter experts, we assumed 75% to be hospitalized. Uncertainty with this proportion was based on a 50% relative increase/decrease on an odds scale.	PERT	Low, modal, high values: 0.667, 0.750, 0.818
Number of deaths	We used the method of Gargano et al. (*23*). In-hospital deaths that occurred in the 2012–2014 HCUP National Inpatient Sample (HCUP NIS) for ICD-9-CM code 031 were combined with out-of-hospital deaths from the National Vital Statistics System (death certificates).	Empirical	Number of deaths by year (2012–2014): 965, 1150, 1035
Proportion travel-related	We assumed that NTM infections were similar to Legionnaires' disease, and used the proportion of patients with *Legionella* infection resulting in Legionnaires' disease who reported travel outside the United States within 30 d of illness onset (2005–2014) in CDC's Supplemental Legionnaires' Disease Surveillance System (*17*). Uncertainty with this proportion was based on a 50% relative increase/decrease on an odds scale.	PERT	Low, modal, high values: 0.0067, 0.01, 0.0149
Proportion waterborne	Structured expert judgment estimate for NTM infection (*9*).	Empirical	2.5th percentile, median, mean, 97.5th percentile: 0.39, 0.73, 0.72, 0.94
Cost of treat-and-release ED visits	Sum of insurer and out-of-pocket payments for treat-and-release ED visits for ICD-9-CM code 031, in 2014 US dollars, in 2012–2013 IBM Watson Health MarketScan research databases, as reported by Adam et al. (*10*).	Empirical	Mean (2.5th percentile, 97.5th percentile): 1,610 (129–6,430)

Pathogen: nontuberculous mycobacterial (NTM) infections			
Model input	Data source(s)	Distribution	Parameters
Cost of hospitalizations	Sum of insurer and out-of-pocket payments for hospitalizations for ICD-9-CM code 031, in 2014 US dollars, in 2012–2013 IBM Watson Health MarketScan research databases.	Empirical	Mean (2.5th percentile, 97.5th percentile): 29,600 (6,350–120,000)
Pathogen-specific limitations and discussion	Pulmonary NTM infections are believed to be the most common manifestation of NTM infection (≈90% of infections are thought to be pulmonary). NTM diagnosis codes are not an exact match to manifestations because the wording for the pulmonary and disseminated codes contain both the manifestation and a species, so they might not accurately capture the true course of illness if clinicians choose the code for the species and not the manifestation. In the Marketscan databases we have observed that persons will often have a disseminated code for one hospitalization and a pulmonary code for the next hospitalization, or vice versa. We chose to not present numbers by individual diagnosis code because we believe the overall numbers are more reliable. The total cost of an NTM infection is likely higher than the cost per hospitalization reported here, because a single infection can have multiple hospitalizations. Because data on the proportion of persons with an NTM infection who have traveled outside of the United States recently were not available, we used the proportion from Legionnaires' disease surveillance. Dedicated surveillance for NTM infectious would address these data gaps.<br>The illness and cost estimates in this work are in the range of with previous work. Previous estimates of the number of NTM infections in 2014 range from 50,976 (*24*) to 181,037 cases (*22*). We estimated 96,953 illnesses occurred (95% CrI 75,739–121,633). Strollo et al. estimated the US cost of pulmonary NTM infections in 2014 to be $1.7 billion, close to the $1.5 billion we estimated for all NTM infections (*22*).		

Pathogen: norovirus			
Model input	Data source(s)	Distribution	Parameters
Reported illnesses	Incidence of illnesses caused by norovirus infection reported to 3 sites (Georgia, Maryland/DC, and Oregon) in the Kaiser Permanente health system (*25,26*).	Mixture of PERTs	69.5/1,000 person-years (Georgia),<br>76.9/1000 person-years (Oregon),<br>61.8/1000 person-years (metro DC area)
Population adjustment (year)	Scaled up to the 2014 US population (*2*).		
Underreporting	Assumed to be equivalent to active surveillance during the study period.	–	–
Underdiagnosis (for number of illnesses)			
Medical care seeking	The Hall and Grytdal incidence estimates were adjusted for the proportion (and 95% CI) of survey respondents among persons with diarrhea who sought medical care, from CDC's Foodborne Diseases Active Surveillance Network (FoodNet) Population Surveys (2000–2001, 2002–2003, 2006–2007) (*3*). No further adjustment was made.	NA	
Specimen submission	The Grytdal et al. estimate was adjusted for the proportion of persons with diarrhea who submitted a fecal sample for bacterial laboratory testing in the Kaiser Permanente health system, while the Hall et al. estimate was adjusted for the proportion (and 95% CI) of survey respondents who submitted a fecal specimen among persons with diarrhea who sought medical care, from FoodNet Population Surveys (2000–2001, 2002–2003, 2006–2007) (*3*). No further adjustment was made.		
Proportion with an ED visit	Estimated annual rate of ED visits per 1,000 persons, from Gastañaduy et al. (*27*). Unlike other diseases in this analysis, the ED visit estimate for norovirus infection includes visits in which the person was admitted to the hospital.	PERT	Low, modal, high values: 0.8, 1.35, 1.89
Number hospitalized	Estimated annual rate of hospitalizations per 100,000 person-years, from Lopman et al. (*28*), applied to the 2014 US population to produce the annual number of hospitalizations.	Empirical	By year (1997–2007):<br>45354, 53608, 67250, 56827, 51306, 69571, 86794, 62477, 67010, 112566, 108927

Pathogen: norovirus			
Model input	Data source(s)	Distribution	Parameters
Number who died	Estimated annual rate of deaths per 1,000 persons, from Hall et al. (*29*), applied to the 2014 US population.	Empirical	By year (1999–2007): 346, 850, 723, 857, 826, 668, 714, 717, 640
Proportion travel-related	Assumed to be low within the incubation period for norovirus.		Low, modal, high values: 0.005, 0.01, 0.02
Proportion waterborne	Structured expert judgment estimate for norovirus (*9*).	Empirical	2.5th percentile, median, mean, 97.5th percentile: 0, 0.03, 0.06, 0.25
Cost of treat-and-release ED visits	Previously reported costs for ED visits, converted to 2014 dollars (*27*).		
Cost of hospitalizations	Previously reported costs for hospitalizations, converted to 2014 dollars (*28*).		
Pathogen-specific limitations and discussion	For norovirus only, costs were derived from previously published costs that did not provide uncertainty intervals. Thus, cost estimates for norovirus do not include credible intervals. The previously published costs were not specifically calculated for norovirus infection and depend on the assumption that costs for norovirus infection are similar to costs for other causes of acute gastroenteritis. The proportion of persons with international travel during the incubation period for norovirus infection was assumed to be low and was not based on information from surveillance. The credible interval for the number of norovirus illnesses that are domestically acquired and waterborne is very wide, reflecting some uncertainty about the true proportion of norovirus infection that is waterborne. For norovirus only, ED visits that resulted in admission to the hospital were included in the count and cost calculation of ED visits. For other diseases, costs of ED visits that resulted in hospitalization were included in hospitalization costs, and not included in emergency visit costs. There were 4,778 hospitalizations for waterborne norovirus (some of these patients could have been admitted to the hospital without an ED visit) and 26,279 ED visits (both treat-and-release and admitted) for waterborne norovirus. If all hospitalizations are assumed to have originated with an ED visit (to estimate the largest possible effect of this double-counting), there could have been as few as 26,279 – 4,778 = 21,501 treat-and-release ED visits, and total costs for norovirus ED visits would be lower by $6,079 $\times$ 4,778 = $5,466,032.		

Syndrome: otitis externa			
Model input	Data source(s)	Distribution	Parameters
Total illnesses	Calculated using the total number of doctors' office visits and ED visits (both treat-and-release and admitted to the hospital) for otitis externa without concurrent otitis media, and the proportion of persons with otitis externa believed to seek medical care.	Nonparametric	Sum of physician office visits and ED visits
Population adjustment (year)	Population ratios applied to each year, 2012–2014, based on US Census population estimates (*2*).	Degenerate	Adjustment by year (2012–2014): 1.01, 1.0, 1.0
Underreporting	All doctors' office visits and ED visits that received an ICD-9-CM code of interest were assumed to be reported to NAMCS and HCUP NEDS.	–	–
Medical care seeking (under-diagnosis factor for illnesses)	A study of >50,000 beachgoers reported that, of beachgoers experiencing an earache after their beach visit, 29.55% sought medical care of any kind (*30*).	PERT	Low, modal, high values: 0.2185, 0.2955, 0.3861
Number of doctors' office visits	Because no national case surveillance system for otitis externa exists, most patients were not expected to be hospitalized, and because diagnosis of otitis externa does not generally rely on laboratory testing, we used the number of doctors' office visits for otitis externa as 1 initial input for the number of total illnesses. All visits in the National Ambulatory Medical Care Survey (for International Classification of Diseases, 9th Edition, Clinical Modification (ICD-9-CM) codes 380.10 (infective otitis externa, unspecified); 380.12 (acute swimmers' ear); and 380.14 (malignant otitis externa). Because it can be difficult to distinguish otitis externa from otitis media and a conservative estimate was desired, all visits with a concurrent diagnosis of	Mixture of normals	By year (2012–2014): 1,648,338; 1,484,991; 909,753

Syndrome: otitis externa			
Model input	Data source(s)	Distribution	Parameters
	ICD-9-CM code 381 (nonsuppurative otitis media and Eustachian tube disorders) or 382 (suppurative and unspecified otitis media) were excluded.		
Number of ED visits (both treat-and-release and admitted to the hospital; used for total illness estimate)	All ED visits (in which the person was not admitted to the hospital) in the 2012–2014 Healthcare Cost and Utilization Project's National Emergency Department Sample (HCUP NEDS) for ICD-9-CM codes 380.10, 380.12, and 380.14 (excluding visits with a concurrent ICD-9-CM code of 381 or 382).	Mixture of normals	By year (2012–2014):<br>378,880; 375,869; 361,076
Number of treat-and-release ED visits	Treat-and-release ED visits (in which the person was not admitted to the hospital) in the 2012–2014 Healthcare Cost and Utilization Project's National Emergency Department Sample (HCUP NEDS) for ICD-9-CM codes 380.10, 380.12, and 380.14 (excluding visits with a concurrent ICD-9-CM code of 381 or 382).	Nonparametric	By year (2012–2014): 367,049; 364,500; 349,206
Number hospitalized	Number of hospitalizations, from the Health Care Utilization Project's National Inpatient Sample, 2012–2014, for ICD-9-CM codes 380.10, 380.12, and 380.14 (excluding hospitalizations with a concurrent ICD-9-CM code of 381 or 382).	Empirical	By year (2012–2014): 15,110; 14,785; 14,400
Number who died	We used the method of Gargano et al. to estimate deaths (*23*). Briefly, in-hospital deaths for otitis externa without concurrent otitis media that occurred in the 2012–2014 HCUP National Inpatient Sample (HCUP NIS) were combined with out-of-hospital deaths from the National Vital Statistics System.	Empirical	By year (2012–2014):115, 130, 150
Underdiagnosis (ED visits, hospitalizations, deaths)	Number of ED visits, hospitalizations, and deaths doubled to account for underdiagnosis.	PERT	Low, modal, high values: 1, 2, 3
Proportion travel-related	We assumed 7% of persons had traveled in the past week before developing otitis externa. Uncertainty with this proportion was based on a 50% relative increase/decrease on an odds scale.	PERT	Low, modal, high values: 0.048, 0.07, 0.10
Proportion waterborne	One study estimated 50% of otitis externa is caused by *Pseudomonas* and 25% by *Staphylococcus aureus* (*31*). We used the structured expert judgment estimates for *Pseudomonas* otitis externa and *Staphylococcus aureus* otitis externa (*9*) and averaged the water attribution rates with a weight ratio of 2:1.	Nonparametric	2.5th percentile, median, mean, 97.5th percentile: 0.67, 0.8, 0.79, 0.95
Cost of treat-and-release ED visits	Sum of insurer and out-of-pocket payments for treat-and-release ED visits for ICD-9-CM codes 380.10, 380.12, and 380.14 (excluding visits with a concurrent ICD-9-CM code of 381 or 382), in 2014 US dollars, in 2012–2013 IBM Watson Health MarketScan research databases, as reported by Adam et al. (*10*).	Empirical	Mean (2.5th percentile, 97.5th percentile): 494 (120–1,430)
Cost of hospitalizations	Sum of insurer and out-of-pocket payments for hospitalizations for ICD-9-CM codes ICD-9-CM codes 380.10, 380.12, and 380.14 (excluding hospitalizations with a concurrent ICD-9-CM code of 381 or 382), in 2014 US dollars, in 2012–2013 IBM Watson Health MarketScan research databases.	Empirical	Mean (2.5th percentile, 97.5th percentile): 12,200 (3,320–42,400)
Pathogen-specific limitations and discussion	We excluded all ED and doctors' office visits with any report of otitis media (because otitis media and otitis externa can be difficult to distinguish clinically) so these numbers are likely an underestimate.<br>Risk of otitis externa (commonly known as "swimmer's ear") is correlated with levels of *Pseudomonas* and other pathogens in water, and increases with bather load in recreational water venues (*32,33*). Risk of otitis externa in beachgoers who enter the water is 1.8 times higher than in beachgoers who do not enter the water (*30*).<br>Otitis externa can be acutely painful and is also a public health problem. Swimming in natural waters has been estimated to cause nearly 1 million excess cases of swimmer's ear in the United States every year (*34*). Otitis externa represents a burden on the healthcare system (an estimated 2.4 million healthcare visits, and nearly half a million hours of clinician time each year [*35*]). Otitis externa also represents a possible source of antimicrobial overuse. Despite clinical guidelines recommending topical treatment for uncomplicated acute otitis externa, one third of outpatient visits involved prescription of systemic antimicrobials for this preventable condition (*36*).<br>Finally, otitis externa is preventable, through keeping ears as dry as possible while swimming, and by making sure the ear is dry after swimming. Ear drops or a hair dryer set on low and held several inches away from the ear can aid in this process.		

Pathogen: *Pseudomonas* pneumonia			
Model input	Data source(s)	Distribution	Parameters
Reported hospitalizations	Number of patients hospitalized, using annual national estimates from the National Inpatient Sample (NIS) (2012–2014) with a primary diagnosis of International Classification of Diseases, 9th Edition, Clinical Modification (ICD-9-CM) code 482.1 (pneumonia due to *Pseudomonas*).	Mixture of normals	By year (2012–2014): 17,040; 15,540; 13,240
Population adjustment (year)	Population ratios applied to each year during 2012–2014 based on US Census population estimates (*2*).	Degenerate	Adjustment by year (2012–2014): 1.01, 1.0, 1.0
Underreporting	All cases with a primary diagnosis of *Pseudomonas* pneumonia in the hospital billing record were assumed to be reported to NIS.	–	–
Underdiagnosis (illnesses, ED visits, hospitalizations, deaths)	Number of ED visits, hospitalizations, and deaths doubled to account for underdiagnosis.	PERT	Low, modal, high values: 1, 2, 3
Proportion hospitalized	After conferring with subject matter experts, we assumed 97% to be hospitalized. Uncertainty with this proportion was based on a 50% relative increase/decrease on an odds scale.	PERT	Low, modal, high values: 0.95, 0.97, 0.99
Proportion with a treat-and-release ED visit	Proportion of treat-and-release ED visits (in which the person was not admitted to the hospital) in the 2012–2014 Healthcare Cost and Utilization Project's National Emergency Department Sample (HCUP NEDS) with a primary diagnosis of ICD-9-CM code 482.1.	Empirical ratio	HCUP ED visits by year (2012–2014): 259, 296, 309<br>HCUP hospitalizations (2012–2014): 17,040; 15,540; 13,240<br>Proportion by year, 2012–2014: 0.02, 0.02, 0.02
Proportion who died	We used the method of Gargano et al. (*26*). In-hospital deaths that occurred in the 2012–2014 HCUP National Inpatient Sample (HCUP NIS) with a primary diagnosis of ICD-9-CM code 482.1 were combined with out-of-hospital deaths from the National Vital Statistics System (death certificates).	Empirical	By year (2012–2014): 790, 555, 450
Proportion travel-related	We assumed *Pseudomonas* pneumonia was similar to Legionnaires' disease, and used the proportion of case-patients with *Legionella* infection resulting in Legionnaires' disease who reported travel outside the United States within 30 d of illness onset (2005–2014) in CDC's Supplemental Legionnaires' Disease Surveillance System (*17*). Uncertainty with this proportion was based on a 50% relative increase/decrease on an odds scale.	PERT	Low, modal, high values: 0.0067, 0.01, 0.0149
Proportion waterborne	Structured expert judgment estimate for *Pseudomonas* pneumonia (*9*).	Empirical	2.5th percentile, median, mean, 97.5th percentile: 0.14, 0.52, 0.51, 0.8
Cost of treat-and-release ED visits	Sum of insurer and out-of-pocket payments for treat-and-release ED visits with a primary diagnosis of ICD-9-CM code 482.1, in 2014 US dollars, in 2012–2013 IBM Watson Health MarketScan research databases, as reported by Adam et al. (*10*).	Empirical	Mean (2.5th percentile, 97.5th percentile): 856 (89–4,190)
Cost of hospitalizations	Sum of insurer and out-of-pocket payments for hospitalizations with a primary diagnosis of ICD-9-CM code 482.1, in 2014 US dollars, in 2012–2013 IBM Watson Health MarketScan research databases.	Empirical	Mean (2.5th percentile, 97.5th percentile): 29,300 (5,910–114,000)
Pathogen-specific limitations and discussion	Because data on the proportion of persons with *Pseudomonas* pneumonia who have traveled outside the United States recently were not available, we used the proportion from Legionnaires' disease surveillance. Because a conservative estimate was desired, we included only hospitalizations and ED visits with a primary diagnosis of *Pseudomonas* pneumonia, which could have excluded some hospitalizations and visits because of waterborne transmission.		

Pathogen: *Pseudomonas* septicemia			
Model input	Data source(s)	Distribution	Parameters
Reported hospitalizations	Number of case-patients hospitalized using annual national estimates from the National Inpatient Sample (NIS) (2012–2014) with a primary diagnosis of International Classification of Diseases, 9th Edition, Clinical Modification (ICD-9-CM) code 038.43 (Septicemia due to other gram-negative organisms – *Pseudomonas*).	Mixture of normals	By year (2012-2014): 17,040; 15,540; 13,240
Population adjustment (year)	Population ratios applied to each year from 2012–2014 based on US Census population estimates (*2*).	Degenerate	Adjustment by year (2012–2014): 1.01, 1.0, 1.0
Underreporting	All cases with a primary diagnosis of *Pseudomonas* septicemia in their hospitalization record were assumed to be reported to NIS.	–	–
Underdiagnosis (illnesses, ED visits, hospitalizations, deaths)	Number of ED visits, hospitalizations, and deaths doubled to account for underdiagnosis.	PERT	Low, modal, high values: 1, 2, 3
Proportion hospitalized	After conferring with CDC experts, we assumed 97% to be hospitalized. Uncertainty with this proportion was based on a 50% relative increase/decrease on an odds scale.	PERT	Low, modal, high values: 0.95, 0.97, 0.99
Proportion with a treat-and-release ED visit	Proportion of treat-and-release ED visits (in which the person was not admitted to the hospital) in the 2012–2014 Healthcare Cost and Utilization Project's National Emergency Department Sample (HCUP NEDS) with a primary diagnosis of ICD-9-CM code 038.43	Empirical ratio	HCUP ED visits by year (2012–2014): 79, 65, 100<br>HCUP hospitalizations (2012–2014): 11,865, 12,570, 13,300<br>Proportion by year, 2012–2014: 0.01, 0.01, 0.01
Proportion who died	We used the method of Gargano et al (*23*). In-hospital deaths that occurred in the 2012–2014 HCUP National Inpatient Sample (HCUP NIS) with a primary diagnosis of ICD-9-CM code 482.1 were combined with out-of-hospital deaths from the National Vital Statistics System (death certificates). Because death certificate data do not contain a specific code for *Pseudomonas* septicemia, we multiplied the number of deaths from "septicemia from other gram-negative organisms" by the proportion of septicemia from other gram-negative organisms attributed to *Pseudomonas* septicemia in HCUP NIS data.	Empirical	By year (2012–2014): 790, 555, 450
Proportion travel-related	We assumed *Pseudomonas* septicemia was similar to Legionnaires' disease, and used the proportion of patients with *Legionella* infection resulting in Legionnaires' disease who reported travel outside the United States within 30 d of illness onset (2005–2014) in CDC's Supplemental Legionnaires' Disease Surveillance System (*17*). Uncertainty with this proportion was based on a 50% relative increase/decrease on an odds scale.	PERT	Low, modal, high values: 0.0067, 0.01, 0.0149
Proportion waterborne	Structured expert judgment estimate for *Pseudomonas* septicemia (*9*).	Empirical	2.5th percentile, median, mean, 97.5th percentile: 0.03, 0.21, 0.22, 0.53
Cost of treat-and-release ED visits	Sum of insurer and out-of-pocket payments for treat-and-release ED visits with a primary diagnosis of ICD-9-CM code 482.1, in 2014 US dollars, in 2012–2013 IBM Watson Health MarketScan research databases, as reported by Adam et al. (*10*).	Empirical	Mean (2.5th percentile, 97.5th percentile): 923 (95–3,190)
Cost of hospitalizations	Sum of insurer and out-of-pocket payments for hospitalizations with a primary diagnosis of ICD-9-CM code 482.1, in 2014 US dollars, in 2012–2013 IBM Watson Health MarketScan research databases.	Empirical	Mean (2.5th percentile, 97.5th percentile): 38,200 (6,340–172,000)
Pathogen-specific limitations and discussion	Because data on the proportion of persons with *Pseudomonas* septicemia who have traveled outside the United States recently were not available, we used the proportion from Legionnaires' disease surveillance. Because a conservative estimate was desired, we included only hospitalizations and ED visits with a primary diagnosis of *Pseudomonas* septicemia, which could have excluded some hospitalizations and visits resulting from waterborne transmission.		

Pathogen: *Salmonella,* nontyphoidal serotypes			
Model input	Data source(s)	Distribution	Parameters
Reported illnesses	Incidence of *Salmonella* infections excluding serotype Typhi reported to CDC's Foodborne Diseases Active Surveillance Network (FoodNet) by FoodNet site (n = 10) and year (2012–	Empirical	By site and year (2012–2015); Appendix 1 Table 2

Pathogen: *Salmonella,* nontyphoidal serotypes			
Model input	Data source(s)	Distribution	Parameters
	2015) (*1*); scaled up to the US population (the FoodNet catchment area covers 10 sites around the United States and represented 15.3% of the US population in the study time period).		
Population adjustment (year)	Incidence of nontyphoidal *Salmonella* in each FoodNet site by year applied to 2014 US Census population estimates (*2*).	Degenerate	Adjustment by year (2012–2015): 1.01, 1.0, 1.0, 0.99
Underreporting	No underreporting multiplier; we assumed that all laboratory-confirmed nontyphoidal *Salmonella* illnesses were enumerated by FoodNet active surveillance.	–	–
Underdiagnosis (for number of illnesses)			
Percent severe	Proportion of cases reporting bloody diarrhea in FoodNet case-control studies of sporadic laboratory-confirmed *Salmonella* infections (*3*). We used uniform minimum variance unbiased (UMVU) estimators for lower and upper endpoints.	PERT	Low, modal, high values: 0.35, 0.45, 0.71
Medical care seeking (severe)	Proportion (and 95% confidence interval (CI)) of survey respondents with bloody diarrhea who sought medical care, from FoodNet Population Surveys (2000–2001, 2002–2003, 2006–2007) (*3*)	PERT	Low, modal, high values: 0.19, 0.35, 0.51
Medical care seeking (mild)	Proportion (and 95% CI) of survey respondents with a non-bloody diarrhea who sought medical care, from FoodNet Population Surveys (2000–2001, 2002–2003, 2006–2007) (*3*).	PERT	Low, modal, high values: 0.15, 0.18, 0.20
Specimen submission (severe)	Proportion (and 95% CI) of survey respondents who submitted a fecal specimen among persons with bloody diarrhea who sought medical care, from FoodNet Population Surveys (2000–2001, 2002–2003, 2006–2007) (*3*).	PERT	Low, modal, high values: 0.11, 0.36, 0.62
Specimen submission (mild)	Proportion (and 95% CI) of survey respondents who submitted a fecal specimen among persons with non-bloody diarrhea who sought medical care, from FoodNet Population Surveys (2000–2001, 2002–2003, 2006–2007) (*3*).	PERT	Low, modal, high values: 0.12, 0.19, 0.25
Laboratory testing	100% of clinical laboratories reported routinely testing fecal samples for *Salmonella* in the FoodNet Laboratory Survey (*4*). As Scallan et al. did (*3*), we assumed a slightly lower rate of 97%; uncertainty with this proportion was based on a 50% relative increase/decrease from 0.97 on an odds scale.	PERT	Low, modal, high values: 0.94, 0.97, 1.00
Laboratory test sensitivity	We assumed a laboratory test sensitivity rate of 70% based on studies of *Salmonella* (*7,8*). We assumed a lower bound of 60% and an upper bound of 90%.	PERT	Low, modal, high values:<br>0.60, 0.70, 0.90
Proportion with a treat-and-release ED visit	Proportion of treat-and-release ED visits (in which the person was not admitted to the hospital) in the 2012–2014 Healthcare Cost and Utilization Project's National Emergency Department Sample (HCUP NEDS) for International Classification of Diseases, 9th Edition, Clinical Modification (ICD-9-CM) code 003 (salmonellosis) compared with hospitalizations in the 2012–2014 HCUP National Inpatient Sample (HCUP NIS) for ICD-9-CM code 003. This proportion was multiplied by the number of FoodNet case-patients with nontyphoidal *Salmonella* infection who were hospitalized.	Empirical ratio	HCUP ED visits by year (2012–2014):<br>1,769, 1,554, 1,742<br>HCUP hospitalizations (2012–2014):<br>10,255, 9,470, 10,260<br>Proportion by year, 2012–2014:<br>0.17, 0.16, 0.17
Proportion hospitalized	Proportion of FoodNet case-patients with nontyphoidal *Salmonella* infection who were hospitalized (2012–2015).	Empirical	By site and year (2012–2015), Appendix 1 Table 3
Proportion who died	Proportion of FoodNet case-patients with nontyphoidal *Salmonella* infection who died (2012–2015).	Empirical	By site and year (2012–2015), Appendix 1 Table 4
Underdiagnosis (ED visits, hospitalizations, deaths)	Number of ED visits, hospitalizations, and deaths doubled to account for underdiagnosis.	PERT	Low, modal, high values: 1, 2, 3
Proportion travel-related	Proportion of FoodNet case-patients with nontyphoidal *Salmonella* infection who reported travel outside the United States within 7 days of illness onset (2012–2015). Uncertainty with this proportion was based on a 50% relative increase/decrease on an odds scale.	PERT	Low, modal, high values: 0.06, 0.096, 0.14
Proportion waterborne	Structured expert judgment estimate for nontyphoidal *Salmonella* infection (*9*).	Empirical	2.5th percentile, median, mean, 97.5th percentile: 0, 0.04, 0.06, 0.22
Cost of treat-and-release ED visits	Sum of insurer and out-of-pocket payments for treat-and-release ED visits for ICD-9-CM code 003 (salmonellosis), in 2014 US dollars, in 2012–2013 IBM Watson Health MarketScan research databases, as reported by Adam et al. (*10*).	Empirical	Mean (2.5th percentile, 97.5th percentile): 1,230 (161–4,500)
Cost of hospitalizations	Sum of insurer and out-of-pocket payments for hospitalizations for ICD-9-CM code 003 (salmonellosis), in 2014 US dollars, in 2012–2013 IBM Watson Health MarketScan research databases.	Empirical	Mean (2.5th percentile, 97.5th percentile): 14,900 (4,300–46,900)

Pathogen: *Salmonella,* nontyphoidal serotypes			
Model input	Data source(s)	Distribution	Parameters
Pathogen-specific limitations and discussion	Emergency department visits used ICD-9-CM codes, which might not fully capture all diagnosed nontyphoidal *Salmonella* infections. The proportion of persons with bloody and non-bloody diarrhea was based on data collected during 2000–2007. Healthcare-seeking behaviors might have changed over time.		

Pathogen: Shiga toxin-producing (STEC) *Escherichia coli* infection, serotype O157			
Model input	Data source(s)	Distribution	Parameters
Reported illnesses	Number of illnesses caused by STEC O157 infection reported to CDC's Foodborne Diseases Active Surveillance Network (FoodNet) by FoodNet site (n = 10) and year (2012–2015) (*1*); scaled up to the US population (the FoodNet catchment area covers 10 sites around the United States and represented 15.3% of the US population in the study time period).	Empirical	By site and year (2012–2015), Appendix 1 Table 2
Population adjustment (year)	Incidence of STEC O157 infection in each FoodNet site by year applied to 2014 US Census population estimates (*2*).	Degenerate	Adjustment by year (2012–2015): 1.01, 1.0, 1.0, 0.99
Underreporting	No underreporting multiplier; we assumed that all laboratory-confirmed STEC O157 illnesses were enumerated by FoodNet active surveillance.	–	–
Underdiagnosis (for number of illnesses)			
Percent severe	Proportion of case-patients by site reporting bloody diarrhea from FoodNet case-control study of sporadic laboratory-confirmed STEC O157 infections (*37*). We used the same lower and upper endpoints derived from Scallan et al. (*3*).	PERT	Low, modal, high values: 0.85, 0.90, 1.00
Medical care seeking (severe)	Proportion (and 95% CI) of survey respondents with bloody diarrhea who sought medical care, from FoodNet Population Surveys (2000–2001, 2002–2003, 2006–2007) (*3*).	PERT	Low, modal, high values: 0.19, 0.35, 0.51
Medical care seeking (mild)	Proportion (and 95% CI) of survey respondents with non-bloody diarrhea who sought medical care, from FoodNet Population Surveys (2000–2001, 2002–2003, 2006–2007) (*3*).	PERT	Low, modal, high values: 0.15, 0.18, 0.20
Specimen submission (severe)	Proportion (and 95% CI) of survey respondents who submitted a fecal specimen among persons with bloody diarrhea who sought medical care, from FoodNet Population Surveys (2000–2001, 2002–2003, 2006–2007) (*3*).	PERT	Low, modal, high values: 0.11, 0.36, 0.62
Specimen submission (mild)	Proportion (and 95% CI) of survey respondents who submitted a fecal specimen among persons with non-bloody diarrhea who sought medical care, from FoodNet Population Surveys (2000–2001, 2002–2003, 2006–2007) (*3*).	PERT	Low, modal, high values: 0.12, 0.19, 0.25
Laboratory testing	Among clinical laboratories that performed on-site testing, proportion that used a method that would isolate STEC O157 in 2014, FoodNet Laboratory Survey (B.B. Bruce, pers. comm. Methods for the FoodNet Laboratory Survey were described by Hoefer et al. [*38*]). Uncertainty with this proportion was based on a 50% relative increase/decrease on an odds scale.	PERT	Low, modal, high values: 0.78, 0.84, 0.89
Test sensitivity	We used a laboratory test sensitivity rate of 70% based on studies of *Salmonella* (*7,8*). We assumed a lower bound of 60% and an upper bound of 90%.	PERT	Low, modal, high values: 0.60, 0.70, 0.90
Proportion with treat-and-release ED visit	Proportion of treat-and-release ED visits (in which the person was not admitted to the hospital) in the 2012–2014 Healthcare Cost and Utilization Project's National Emergency Department Sample (HCUP NEDS) for International Classification of Diseases, 9th Edition, Clinical Modification (ICD-9-CM) code 041.41 (STEC O157 infection) compared to hospitalizations in the 2012–2014 HCUP National Inpatient Sample (HCUP NIS) for ICD-9-CM code 041.41. This proportion was multiplied by the number of FoodNet case-patients with STEC O157 infection who were hospitalized.	Empirical ratio	HCUP ED visits by year (2012–2014): 118, 134, 124<br>HCUP hospitalizations (2012–2014): 770, 880, 695<br>Proportion by year, 2012–2014: 0.15, 0.15, 0.18
Proportion hospitalized	Proportion of FoodNet case-patients with STEC O157 infection who were hospitalized.	Empirical	By site and year (2012–2015), Appendix 1 Table 3
Proportion who died	Proportion of FoodNet case-patients with STEC O157 infection who died.	Empirical	By site and year (2012–2015), Appendix 1 Table 4

Pathogen: Shiga toxin-producing (STEC) *Escherichia coli* infection, serotype O157			
Model input	Data source(s)	Distribution	Parameters
Underdiagnosis (ED visits, hospitalizations, deaths)	Number of ED visits, hospitalizations and deaths doubled to account for underdiagnosis.	PERT	Low, modal, high values: 1, 2, 3
Proportion travel-related	Proportion of FoodNet case-patients with STEC O157 infection who reported travel outside the United States within 7 d of illness onset (2012–2015). Uncertainty with this proportion was based on a 50% relative increase/decrease on an odds scale.	PERT	Low, modal, high values: 0.05, 0.10, 0.21
Proportion waterborne	Structured expert judgment estimate for STEC O157 infection (*9*).	Empirical	2.5th percentile, median, mean, 97.5th percentile: 0.01, 0.05, 0.05, 0.13
Cost of treat-and-release ED visits	Sum of insurer and out-of-pocket payments for treat-and-release ED visits for ICD-9-CM codes 041.41 and 041.42, in 2014 US dollars, in 2012–2013 IBM Watson Health MarketScan research databases. Costs for STEC O157 and STEC non-O157 were combined and payer proportion was derived from all ED visits instead of treat-and-release visits because of small sample size.	Empirical	Mean (2.5th percentile, 97.5th percentile): 1,070 (109–2,350)
Cost of hospitalizations	Sum of insurer and out-of-pocket payments for hospitalizations for ICD-9-CM code 041.41, in 2014 US dollars, in 2012–2013 IBM Watson Health MarketScan research databases.	Empirical	Mean (2.5th percentile, 97.5th percentile): 19,000 (3,790–85,000)
Pathogen-specific limitations and discussion	Scallan et al. (*3*) used the proportion of laboratories routinely testing for STEC O157, but we used the proportion of laboratories that could perform a test that would isolate STEC O157, whether they tested fecal samples routinely or upon physician request. We did this because a conservative estimate was desired, and because laboratory testing capability could have changed over time. Infections caused by STEC O157 have decreased in the past 10 y (*1*). The increasing use of culture-independent diagnostic tests (CIDTs) makes interpretation of trends in STEC infections difficult because CIDTs do not indicate which STEC serogroup caused the infection. The number of CIDT positive–only infections reported to FoodNet has been increasing markedly since 2013, as more clinical laboratories adopt CIDTs. Initially, increases were primarily limited to *Campylobacter* and STEC.		

Pathogen: Shiga toxin-producing *Escherichia coli* (STEC) infection, serotype non-O157			
Model input	Data source(s)	Distribution	Parameters
Reported illnesses	Number of illnesses caused by STEC non-O157 infection reported to CDC's Foodborne Diseases Active Surveillance Network (FoodNet) by FoodNet site (n = 10) and year (2012–2015) (*1*); scaled up to the US population (the FoodNet catchment area covers 10 sites around the United States and represented 15.3% of the US population in the study time period).	Empirical	By site and year (2012–2015), Appendix 1 Table 2
Population adjustment (year)	Incidence of STEC non-O157 infection in each FoodNet site by year, applied to 2014 US Census population estimates (*2*).	Degenerate	Adjustment by year (2012–2015): 1.01, 1.0, 1.0, 0.99
Underreporting	No underreporting multiplier; we assumed that all laboratory-confirmed non-O157 STEC illnesses were enumerated by FoodNet active surveillance.	–	–
Underdiagnosis (for number of illnesses)			
Percent severe	Proportion of non-O157 STEC cases of infection with bloody diarrhea from published studies in FoodNet sites (*39,40*). Uncertainty with this proportion (54%) was based on a 50% relative increase/decrease from 0.54 on an odds scale.	PERT	Low, modal, high values: 0.44, 0.54, 0.64
Medical care seeking (severe)	Proportion (and 95% CI) of survey respondents with bloody diarrhea who sought medical care, from FoodNet Population Surveys (2000–2001, 2002–2003, 2006–2007) (*3*).	PERT	Low, modal, high values: 0.19, 0.35, 0.51
Medical care seeking (mild)	Proportion (and 95% CI) of survey respondents with a non-bloody diarrhea who sought medical care, from FoodNet Population Surveys (2000–2001, 2002–2003, 2006–2007) (*3*).	PERT	Low, modal, high values: 0.15, 0.18, 0.20
Specimen submission (severe)	Proportion (and 95% CI) of survey respondents who submitted a fecal specimen among persons with bloody diarrhea who sought medical care, from FoodNet Population Surveys (2000–2001, 2002–2003, 2006–2007) (*3*).	PERT	Low, modal, high values: 0.11, 0.36, 0.62
Specimen submission (mild)	Proportion (and 95% CI) of survey respondents who submitted a fecal specimen among persons with non-bloody diarrhea who sought medical care, from FoodNet Population Surveys (2000–2001, 2002–2003, 2006–7) (*3*).	PERT	Low, modal, high values: 0.12, 0.19, 0.25

Pathogen: Shiga toxin-producing *Escherichia coli* (STEC) infection, serotype non-O157			
Model input	Data source(s)	Distribution	Parameters
Laboratory testing	Among clinical laboratories that performed on-site testing, proportion that used a method that would isolate STEC non-O157 in 2014, from a FoodNet Laboratory Survey (B.B. Bruce, pers. comm.). Methods for the FoodNet Laboratory Survey were described by Hoefer et al. (*38)*. Uncertainty with this proportion was based on a 50% relative increase/decrease on an odds scale.	PERT	Low, modal, high values: 0.45, 0.55, 0.65
Test sensitivity	We used a laboratory test sensitivity rate of 70% based on studies of *Salmonella* (*7,8*). We assumed a lower bound of 60% and an upper bound of 90%.	PERT	Low, modal, high values: 0.60, 0.70, 0.90
Proportion with treat-and-release ED visit	Proportion of treat-and-release ED visits (in which the person was not admitted to the hospital) in the 2012–2014 Healthcare Cost and Utilization Project's National Emergency Department Sample (HCUP NEDS) for International Classification of Diseases, 9th Edition, Clinical Modification (ICD-9-CM) code 041.42 (STEC O157 infection) compared with hospitalizations in the 2012–2014 HCUP National Inpatient Sample (HCUP NIS) for ICD-9-CM code 041.42. This proportion was multiplied by the number of FoodNet case-patients with STEC non-O157 infection who were hospitalized.	Empirical ratio	HCUP ED visits by year (2012–2014): 48, 38, 25<br>HCUP hospitalizations (2012–2014): 160, 305, 255<br>Proportion by year, 2012–2014: 0.30, 0.12, 0.10
Proportion hospitalized	Proportion of FoodNet case-patients with non-O157 STEC infection who were hospitalized.	Empirical	By site and year (2012–2015), Appendix 1 Table 3
Proportion who died	Proportion of FoodNet case-patients with non-O157 STEC infection who died.	Empirical	By site and year (2012–2015), Appendix 1 Table 4
Underdiagnosis (ED visits, hospitalizations, deaths)	Number of ED visits, hospitalizations, and deaths doubled to account for underdiagnosis.	PERT	Low, modal, high values: 1, 2, 3
Proportion travel-related	Proportion of FoodNet case-patients with non-O157 STEC infection who reported travel outside the United States within 7 d of illness onset (2012–2015). Uncertainty with this proportion was based on a 50% relative increase/decrease on an odds scale.	PERT	Low, modal, high values: 0.05, 0.095, 0.21
Proportion waterborne	Structured expert judgment estimate for non-O157 STEC infection (*9*).	Empirical	2.5th percentile, median, mean, 97.5th percentile: 0, 0.05, 0.06, 0.17
Cost of treat-and-release ED visits	Sum of insurer and out-of-pocket payments for treat-and-release ED visits for ICD-9-CM codes 041.41 and 041.42, in 2014 US dollars, in 2012–2013 IBM Watson Health MarketScan research databases. Costs for STEC O157 and STEC non-O157 were combined and payer proportion was derived from all ED visits instead of treat-and-release visits because of small sample size.	Empirical	Mean (2.5th percentile, 97.5th percentile): 1,070 (109–2,350)
Cost of hospitalizations	Sum of insurer and out-of-pocket payments for hospitalizations for ICD-9-CM code 041.42, in 2014 US dollars, in 2012–2013 IBM Watson Health MarketScan research databases.	Empirical	Mean (2.5th percentile, 97.5th percentile): 24,200 (4,780–138,000)
Pathogen-specific limitations and discussion	The increase in STEC incidence is driven by the increase in STEC non-O157, which is not typically included in routine fecal culture testing because it requires specialized methods (*1*). Routine fecal cultures performed in clinical laboratories typically include methods that identify only *Salmonella*, *Campylobacter*, *Shigella*, and, for some laboratories, STEC O157. The increased use of the syndrome panel tests might increase identification, and, thus, improve incidence estimates of pathogens for which testing was previously limited.		

Pathogen: *Shigella* spp.			
Model input	Data source(s)	Distribution	Parameters
Reported illnesses	Number of illnesses caused by *Shigella* spp. infection reported to CDC's Foodborne Diseases Active Surveillance Network (FoodNet) by FoodNet site (n = 10) and year (2012–2015) (*1*); scaled up to the US population (the FoodNet catchment area covers 10 sites around the United States and represented 15.3% of the US population in the study time period).	Empirical	By site and year (2012–2015), Appendix 1 Table 2
Population adjustment (year)	Incidence of *Shigella* spp. infection in each FoodNet site by year applied to 2014 US Census population estimates (*2*).	Degenerate	Adjustment by year (2012–2015): 1.01, 1.0, 1.0, 0.99

Pathogen: *Shigella* spp.			
Model input	Data source(s)	Distribution	Parameters
Underreporting	No underreporting multiplier; we assumed that all laboratory-confirmed *Shigella* spp. illnesses were enumerated by FoodNet active surveillance.	–	–
Underdiagnosis (for number of illnesses)			
Percent severe	Percent of laboratory-confirmed cases of *Shigella* spp. infection with bloody diarrhea reported to FoodNet surveillance in Minnesota and New York (*3*). We used uniform minimum variance unbiased (UMVU) estimators for lower and upper endpoints.	PERT	Low, modal, high values: 0.17, 0.35, 0.53
Medical care seeking (severe)	Proportion (and 95% CI) of survey respondents with bloody diarrhea who sought medical care, from FoodNet Population Surveys (2000–2001, 2002–2003, 2006–2007) (*3*).	PERT	Low, modal, high values: 0.19, 0.35, 0.51
Medical care seeking (mild)	Proportion (and 95% CI) of survey respondents with a non-bloody diarrhea who sought medical care, from FoodNet Population Surveys (2000–2001, 2002–2003, 2006–2007) (*3*).	PERT	Low, modal, high values: 0.15, 0.18, 0.20
Specimen submission (severe)	Proportion (and 95% CI) of survey respondents who submitted a fecal specimen among persons with bloody diarrhea who sought medical care, from FoodNet Population Surveys (2000–2001, 2002–2003, 2006–2007) (*3*).	PERT	Low, modal, high values: 0.11 0.36, 0.62
Specimen submission (mild)	Proportion (and 95% CI) of survey respondents who submitted a fecal specimen among persons with non-bloody diarrhea who sought medical care, from FoodNet Population Surveys (2000–2001, 2002–2003, 2006–2007) (*3*).	PERT	Low, modal, high values: 0.12, 0.19, 0.25
Laboratory testing	Proportion of clinical laboratories routinely testing fecal samples for *Shigella* spp., from the FoodNet Laboratory Survey (*4*). We assumed a slightly lower rate of 97%; uncertainty with this proportion was based on a 50% relative increase/decrease from 0.97 on an odds scale.	PERT	Low, modal, high values: 0.94, 0.97, 1.00
Test sensitivity	We used a laboratory test sensitivity rate of 70% based on studies of *Salmonella* (*7,8*). We assumed a lower bound of 60% and an upper bound of 90%.	PERT	Low, modal, high values: 0.60, 0.70, 0.90
Proportion with a treat-and-release ED visit	Proportion of treat-and-release ED visits (in which the person was not admitted to the hospital) in the 2012–2014 Healthcare Cost and Utilization Project's National Emergency Department Sample (HCUP NEDS) for International Classification of Diseases, 9th Edition, Clinical Modification (ICD-9-CM) code 004 (shigellosis) compared with hospitalizations in the 2012–2014 HCUP National Inpatient Sample (HCUP NIS) for ICD-9-CM code 004. This proportion was multiplied by the number of FoodNet case-patients with *Shigella* spp. infection who were hospitalized.	Empirical ratio	HCUP ED visits by year (2012–2014): 867, 652, 935<br>HCUP hospitalizations (2012–2014): 1,650, 1,405, 2,075<br>Proportion by year, 2012–2014: 0.53, 0.46, 0.46
Proportion hospitalized	Proportion of FoodNet case-patients with *Shigella* spp. infection who were hospitalized (2012–2015).	Empirical	By site and year (2012–2015), Appendix 1 Table 3
Proportion who died	Proportion of FoodNet case-patients with *Shigella* spp. infection who died (2012–2015).	Empirical	By site and year (2012–2015), Appendix 1 Table 4
Underdiagnosis (ED visits, hospitalizations, deaths)	Number of ED visits, hospitalizations and deaths doubled to account for underdiagnosis.	PERT	Low, modal, high values: 1, 2, 3
Proportion travel-related	Proportion of FoodNet case-patients with *Shigella* spp. infection who reported travel outside the United States within 7 d of illness onset (2012–2015). Uncertainty with this proportion was based on a 50% relative increase/decrease on an odds scale.	PERT	Low, modal, high values: 0.05, 0.078, 0.11
Proportion waterborne	Structured expert judgment estimate for *Shigella* spp. infection (*9*).	Empirical	2.5th percentile, median, mean, 97.5th percentile: 0.01, 0.03, 0.04, 0.21
Cost of treat-and-release ED visits	Sum of insurer and out-of-pocket payments for treat-and-release emergency department visits for ICD-9-CM code 004 (shigellosis), in 2014 US dollars, in 2012–2013 IBM Watson Health MarketScan research databases, as reported in Adam et al. (*10*).	Empirical	Mean (2.5th percentile, 97.5th percentile): 952 (115–3,980)
Cost of hospitalizations	Sum of insurer and out-of-pocket payments for hospitalizations for ICD-9-CM code 004, in 2014 US dollars, in 2012–2013 IBM Watson Health MarketScan research databases.	Empirical	Mean (2.5th percentile, 97.5th percentile): 14,200 (4,130–48,000)
Pathogen-specific limitations and discussion	The majority of *Shigella* spp. transmission in the United States is fecal–oral, transmitted person to person or through contaminated food.		

Pathogen: *Vibrio* spp., all			
Model input	Data source(s)	Distribution	Parameters
Reported illnesses	Sum of illnesses caused by *Vibrio spp.* including *V. alginolyticus, V. vulnificus*, *V. parahaemolyticus*, and other species reported to CDC's Cholera and Other Vibrio Illness Surveillance (COVIS) System (2008–2014) (*41*). Because of an apparent trend over time, linear regression was used to estimate the projected illness for reference year 2014. The uncertainty around the estimated illness was based on the residuals from linear regression (see Appendix 2 for more information).	Empirical	By year (2008–2014): 599, 825, 927, 853, 944, 1176, 1252
Population adjustment (year)	Population ratios applied to each year during 2008–2014 based on US Census population estimates (*2*).	Degenerate	Adjustment by year (2008–2014): 1.05, 1.04, 1.03, 1.02, 1.01, 1.0, 1.0
Underreporting	Passive surveillance multiplier used to adjust for underreporting (*3*).	PERT	Low, modal, high values: 0.9, 1.1, 1.3
Underdiagnosis (for number of illnesses)			
Percent severe	Assumed to be a similar illness to non-typhoidal *Salmonella* infection.	PERT	Low, modal, high values: 0.35, 0.45, 0.71
Medical care seeking (severe)	Proportion (and 95% CI) of survey respondents with bloody diarrhea who sought medical care, from FoodNet Population Surveys (2000–2001, 2002–2003, 2006–2007) (*3*)	PERT	Low, modal, high values: 0.19, 0.35, 0.51
Medical care seeking (mild)	Proportion (and 95% CI) of survey respondents with non-bloody diarrhea who sought medical care, from FoodNet Population Surveys (2000–2001, 2002–2003, 2006–2007) (*3*).	PERT	Low, modal, high values: 0.15, 0.18, 0.20
Specimen submission (severe)	Proportion (and 95% CI) of survey respondents who submitted a fecal specimen among persons with bloody diarrhea who sought medical care, from FoodNet Population Surveys (2000–2001, 2002–2003, 2006–2007) (*3*).	PERT	Low, modal, high values: 0.10, 0.36, 0.62
Specimen submission (mild)	Proportion (and 95% CI) of survey respondents who submitted a fecal specimen among persons with a non-bloody diarrhea who sought medical care from FoodNet Population Surveys (2000–2001, 2002–2003, 2006–2007) (*3*).	PERT	Low, modal, high values:<br>0.12, 0.19, 0.25
Laboratory testing	Proportion of clinical laboratories routinely testing fecal samples for *Vibrio spp.,* from the FoodNet Laboratory Survey (4).	PERT	Low, modal, high values: 0.41, 0.51, 0.61
Test sensitivity	Proportion of clinical laboratories using appropriate diagnostic tests to test fecal samples for *Vibrio* spp., from the FoodNet Laboratory Survey (*4*).	PERT	Low, modal, high values: 0.21, 0.28, 0.37
Proportion with a treat-and-release ED visit	Proportion of treat-and-release ED visits (in which the person was not admitted to the hospital) in the 2012–2014 Healthcare Cost and Utilization Project's National Emergency Department Sample (HCUP NEDS) for International Classification of Diseases, 9th Edition, Clinical Modification (ICD-9-CM) code 001.0 (cholera due to *Vibrio cholerae*), 001.1 (cholera due to *Vibrio cholera* El Tor), 001.9 (cholera, unspecified), 005.4 (food poisoning due to *Vibrio parahaemolyticus*), and 005.81 (food poisoning due to *Vibrio vulnificus*) compared to hospitalizations in the 2012–2014 HCUP National Inpatient Sample (HCUP NIS) for the same ICD-9-CM codes.	Empirical ratio	HCUP ED visits by year (2012–2014): 155, 197, 191<br>HCUP hospitalizations (2012–2014): 100, 175, 80<br>Proportion by year, 2012–2014: 1.55, 1.13, 2.39
Proportion hospitalized	Proportion of case-patients with *Vibrio spp.* infection reported to COVIS who were hospitalized (2008–2014).	Empirical	By year (2008–2014): 0.40, 0.36, 0.32, 0.34, 0.35, 0.35, 0.27
Proportion who died	Proportion of case-patients with *Vibrio spp.* infection reported to COVIS who died (2008–2014).	Empirical	By year (2008–2014): 0.06, 0.06, 0.06, 0.06, 0.06, 0.04, 0.04
Underdiagnosis (ED visits, hospitalizations, deaths)	Number of ED visits, hospitalizations, and deaths doubled to account for underdiagnosis.	PERT	Low, modal, high values: 1, 2, 3
Proportion travel-related	Based on proportion of case-patients with *Vibrio spp.* infection reported to COVIS who acquired the infection while traveling outside the United States in the 7 d before illness onset (2008–2014).	PERT	Low, modal, high values: 0.0992, 0.1339, 0.2275
Proportion waterborne	Structured expert judgment estimate for *Vibrio spp.* infection (*9*).	Empirical	2.5th percentile, median, mean, 97.5th percentile: 0.07, 0.24, 0.24, 0.38
Cost of treat-and-release ED visits	Sum of insurer and out-of-pocket payments for treat-and-release emergency department visits for ICD-9-CM codes 001, 005.4, and 005.81, in 2014 US dollars, in 2012–2013 IBM Watson Health MarketScan research databases, as reported by Adam et al. (*10*).	Empirical	Mean (2.5th percentile, 97.5th percentile): 1,030 (293–3,330)
Cost of hospitalizations	Sum of insurer and out-of-pocket payments for hospitalizations for ICD-9-CM codes 001, 005.4, and 005.81, in 2014 US dollars, in 2012–2013 IBM Watson Health MarketScan research databases.	Empirical	Mean (2.5th percentile, 97.5th percentile): 16,000 (3,780–39,900)

Pathogen: *Vibrio* spp., all			
Model input	Data source(s)	Distribution	Parameters
Pathogen-specific limitations and discussion	*Vibrio spp.* infection manifests in many different ways (e.g., acute gastrointestinal illness, wound infection, bacteremia). Medical care-seeking proportions likely differ for different manifestations, but the medical care-seeking proportions for acute gastrointestinal illness were used, because data were not available for other manifestations.		

Pathogen: *Vibrio alginolyticus*			
Model input	Data source(s)	Distribution	Parameters
Reported illnesses	Number of illnesses caused by *V. alginolyticus* reported to CDC's Cholera and Other Vibrio Illness Surveillance (COVIS) System (2008–2014) (*41*). Because of an apparent trend over time, linear regression was used to estimate the projected illness for reference year 2014. The uncertainty around the estimated illness was based on the residuals from linear regression (see Appendix 2 for more information).	Empirical	By year (2008–2014): 103, 129, 152, 157, 188, 205, 241
Population adjustment (year)	Population ratios applied to each year during 2008–2014 based on US Census population estimates (*2*).	Degenerate	Adjustment by year (2008–2014): 1.05, 1.04, 1.03, 1.02, 1.01, 1.0, 1.0
Underreporting	Passive surveillance multiplier used to adjust for underreporting (*3*).	PERT	Low, modal, high values: 0.9, 1.1, 1.3
Underdiagnosis (for number of illnesses)			
Percent severe	Assumed to be a similar illness to nontyphoidal *Salmonella* infection.	PERT	Low, modal, high values: 0.35, 0.45, 0.71
Medical care seeking (severe)	Proportion (and 95% CI) of survey respondents with bloody diarrhea who sought medical care, from FoodNet Population Surveys (2000–2001, 2002–2003, 2006–2007) (*3*)	PERT	Low, modal, high values: 0.19, 0.35, 0.51
Medical care seeking (mild)	Proportion (and 95% CI) of survey respondents with a non-bloody diarrhea who sought medical care, from FoodNet Population Surveys (2000–2001, 2002–2003, 2006–2007) (*3*).	PERT	Low, modal, high values: 0.15, 0.18, 0.20
Specimen submission (severe)	Proportion (and 95% CI) of survey respondents who submitted a fecal specimen among persons with bloody diarrhea who sought medical care, from FoodNet Population Surveys (2000–2001, 2002–2003, 2006–2007) (*3*).	PERT	Low, modal, high values: 0.11, 0.36, 0.62
Specimen submission (mild)	Proportion (and 95% CI) of survey respondents who submitted a fecal specimen among persons with a non-bloody diarrhea who sought medical care, from FoodNet Population Surveys (2000–2001, 2002–2003, 2006–2007) (*3*).	PERT	Low, modal, high values: 0.12, 0.19, 0.25
Laboratory testing	Proportion of clinical laboratories routinely testing fecal samples for *Vibrio* spp., from the FoodNet Laboratory Survey (*4*).	PERT	Low, modal, high values: 0.41, 0.51, 0.61
Test sensitivity	Proportion of clinical laboratories using appropriate diagnostic tests to test fecal samples for *Vibrio* spp., from the FoodNet Laboratory Survey (*4*).	PERT	Low, modal, high values: 0.21, 0.28, 0.37
Proportion with a treat-and-release ED visit	Could not calculate because there is no *V. alginolyticus*-specific International Classification of Diseases, 9th Edition, Clinical Modification (ICD-9-CM) code.		
Proportion hospitalized	Proportion of case-patients with *Vibrio alginolyticus* infection reported to COVIS who were hospitalized (2008–2014).	Empirical	By year (2008–2014): 0.25, 0.13, 0.14, 0.11, 0.13, 0.21, 0.14
Proportion who died	Proportion of case-patients with *Vibrio alginolyticus* reported to COVIS who died (2008–2014).	Empirical	By year (2008–2014): 0.02, 0.02, 0.01, 0.00, 0.01, 0.01, 0.00
Underdiagnosis (ED visits, hospitalizations, deaths)	Number of hospitalizations and deaths doubled to account for underdiagnosis.	PERT	Low, modal, high values: 1, 2, 3
Proportion travel-related	Based on proportion of case-patients with *Vibrio alginolyticus* infection reported to COVIS who acquired the infection while traveling outside the United States in the 7 d before illness onset (2008–2014).	PERT	Low, modal, high values: 0.0367, 0.0667, 0.0838
Proportion waterborne	Structured expert judgment estimate for *Vibrio alginolyticus* infection (*9*).	Empirical	2.5th percentile, median, mean, 97.5th percentile: 0.13, 0.36, 0.37, 0.71
Cost of treat-and-release ED visits	Could not calculate because there is no *V. alginolyticus*-specific ICD-9-CM code.		
Cost of hospitalizations	Could not calculate because there is no *V. alginolyticus*-specific ICD-9-CM code.		
Pathogen-specific limitations and discussion	Could not calculate costs because of poor ICD-9 code fit.		

Pathogen: *Vibrio parahaemolyticus*			
Model input	Data source(s)	Distribution	Parameters
Reported illnesses	Number of illnesses due to *Vibrio parahaemolyticus* infection reported to CDC's Cholera and Other Vibrio Illness Surveillance (COVIS) System (2008–2014) (*41*). Because of an apparent trend over time, linear regression was used to estimate the projected illness for reference year 2014. The uncertainty around the estimated illness was based on the residuals from linear regression (see Appendix 2 for more information).	Empirical	By year (2008–2014): 270, 386, 421, 334, 431, 594, 605
Population adjustment (year)	Population ratios applied to each year during 2008–2014 based on US Census population estimates (*2*).	Degenerate	Adjustment by year (2008–2014): 1.05, 1.04, 1.03, 1.02, 1.01, 1.0, 1.0
Underreporting	Passive surveillance multiplier used to adjust for underreporting (*3*).	PERT	Low, modal, high values: 0.9, 1.1, 1.3
Underdiagnosis (for number of illnesses)			
Percent severe	Assumed to be a similar illness to nontyphoidal *Salmonella* infection.	PERT	Low, modal, high values: 0.35, 0.45, 0.71
Medical care seeking (severe)	Proportion (and 95% CI) of survey respondents with bloody diarrhea who sought medical care, from FoodNet Population Surveys (2000–2001, 2002–2003, 2006–2007) (*3*)	PERT	Low, modal, high values: 0.19, 0.35, 0.51
Medical care seeking (mild)	Proportion (and 95% CI) of survey respondents with non-bloody diarrhea who sought medical care from FoodNet Population Surveys (2000–2001, 2002–2003, 2006–2007) (*3*).	PERT	Low, modal, high values: 0.15, 0.18, 0.20
Specimen submission (severe)	Proportion (and 95% CI) of survey respondents who submitted a fecal specimen among persons with bloody diarrhea who sought medical care, from FoodNet Population Surveys (2000–2001, 2002–2003, 2006–2007) (*3*).	PERT	Low, modal, high values: 0.11, 0.36, 0.62
Specimen submission (mild)	Proportion (and 95% CI) of survey respondents who submitted a fecal specimen among persons with a non-bloody diarrhea who sought medical care from FoodNet Population Surveys (2000–2001, 2002–2003, 2006–2007) (*3*).	PERT	Low, modal, high values: 0.12, 0.19, 0.25
Laboratory testing	Proportion of clinical laboratories routinely testing fecal samples for *Vibrio* spp., from the FoodNet Laboratory Survey (*4*).	PERT	Low, modal, high values: 0.41, 0.51, 0.61
Test sensitivity	Proportion of clinical laboratories using appropriate diagnostic tests to test fecal samples for *Vibrio* spp., from the FoodNet Laboratory Survey (*4*).	PERT	Low, modal, high values: 0.21, 0.28, 0.37
Proportion with a treat-and-release ED visit	Could not calculate because of a poor fit between International Classification of Diseases, 9th Edition, Clinical Modification (ICD-9-CM) codes and infections by individual *Vibrio* spp.		
Proportion hospitalized	Proportion of case-patients with *Vibrio parahaemolyticus* infection reported to COVIS who were hospitalized (2008–2014).	Empirical	By year (2008–2014): 0.260, 0.230, 0.220, 0.240, 0.250, 0.210, 0.150
Proportion who died	Proportion of case-patients with *Vibrio parahaemolyticus* infection reported to COVIS who died (2008–2014).	Empirical	By year (2008–2014): 0.020, 0.010, 0.010, 0.020, 0.020, 0.007, 0.010
Underdiagnosis (ED visits, hospitalizations, deaths)	Number of ED visits, hospitalizations, and deaths doubled to account for underdiagnosis.	PERT	Low, modal, high values: 1, 2, 3
Proportion travel-related	Based on proportion of case-patients with *Vibrio parahaemolyticus* infection reported to COVIS who acquired the infection while traveling outside the United States in the 7 d before illness onset (2008–2014).	PERT	Low, modal, high values: 0.0512, 0.0627, 0.1007
Proportion waterborne	Structured expert judgment estimate for *Vibrio parahaemolyticus* infection (*9*).	Empirical	2.5^{th} percentile, median, mean, 97.5^{th} percentile: 0.07, 0.24, 0.24, 0.38
Cost of treat-and-release emergency visits	Could not calculate because of a poor fit between ICD-9-CM codes and infections by individual *Vibrio* species.		
Cost of hospitalizations	Could not calculate because of a poor fit between ICD-9-CM codes and infections by individual *Vibrio* species.		
Pathogen-specific limitations and discussion	Could not calculate costs because of poor ICD-9 code fit.		

Pathogen: *Vibrio vulnificus*			
Model input	Data source(s)	Distribution	Parameters
Reported illnesses	Number of illnesses caused by *Vibrio vulnificus* infection reported to CDC's Cholera and Other Vibrio Illness Surveillance (COVIS) System (2008–2014) (*41*). Because of an apparent trend over time, linear regression was used to estimate the projected illness for reference year 2014. The uncertainty around the estimated illness was based on the residuals from linear regression (see Appendix 2 for more information).	Empirical	By year (2008–2014): 85, 107, 133, 113, 119, 137, 124
Population adjustment (year)	Population ratios applied to each year during 2008–2014 based on US Census population estimates (*2*).	Degenerate	Adjustment by year (2008–2014): 1.05, 1.04, 1.03, 1.02, 1.01, 1.0, 1.0
Underreporting	Passive surveillance multiplier used to adjust for underreporting (*3*).	PERT	Low, modal, high values: 0.9, 1.1, 1.3
Underdiagnosis (for number of illnesses)			
Percent severe	Almost all cases assumed to be severe.	PERT	Low, modal, high values: 0.95, 1, 1
Medical care seeking (severe)	Assumed to have a high rate of medical care seeking.	PERT	Low, modal, high values: 0.80, 0.90, 1.00
Medical care seeking (mild)	Assumed to have a high rate of medical care seeking.	PERT	Low, modal, high values: 0.80, 0.90, 1.00
Specimen submission (severe)	Assumed to have a high rate of specimen submission.	PERT	Low, modal, high values: 0.70, 0.80, 0.90
Specimen submission (mild)	Assumed to have a high rate of specimen submission.	PERT	Low, modal, high values: 0.70, 0.80, 0.90
Laboratory testing	We assumed that most persons with *Vibrio vulnificus* who submitted a specimen for testing would be tested.	PERT	Low, modal, high values: 0.94, 0.97, 1.00
Test sensitivity	Based on sensitivity of blood cultures (*42,43*).	PERT	Low, modal, high values: 0.70, 0.85, 1.00
Proportion with a treat-and-release ED visit	Could not calculate because of a poor fit between International Classification of Diseases, 9th Edition, Clinical Modification (ICD-9-CM) codes and infections by individual *Vibrio* species		
Proportion hospitalized	Proportion of case-patients with *Vibrio vulnificus* infection reported to COVIS who were hospitalized (2008–2014).	Empirical	By year (2008–2014): 0.86, 0.90, 0.75, 0.87, 0.87, 0.94, 0.79
Proportion who died	Proportion of case-patients with *Vibrio vulnificus* infection reported to COVIS who died (2008–2014).	Empirical	By year (2008–2014): 0.30, 0.32, 0.31, 0.31, 0.32, 0.28, 0.18
Underdiagnosis (ED visits, hospitalizations, deaths)	Underdiagnosis/reporting for hospitalizations and deaths (UDR H/D) were set to be the product of underdiagnosis for illness and under-reporting for illness. Underdiagnosis/ reporting for ED visit was set using a PERT distribution with parameters of (1, 2, 3).	Empirical	UDR H/D: 2.5%, median and 97.5%: 1.483, 1.855, 2.321
Proportion travel-related	Based on proportion of case-patients with *Vibrio vulnificus* infection reported to COVIS who acquired the infection while traveling outside the United States in the 7 d before illness onset (2008–2014).	PERT	Low, modal, high values: 0, 0.0111, 0.045
Proportion waterborne	Structured expert judgment estimate for *Vibrio vulnificus* infection (*9*).	Empirical	2.5th percentile, median, mean, 97.5th percentile: 0.4, 0.8, 0.77, 0.91
Cost of treat-and-release ED visits	Could not calculate because of a poor fit between ICD-9-CM codes and infections by individual *Vibrio* species.		
Cost of hospitalizations	Could not calculate because of a poor fit between ICD-9-CM codes and infections by individual *Vibrio* species.		
Pathogen-specific limitations and discussion	Could not calculate costs because of poor ICD-9 code fit.		

Pathogen: *Vibrio* spp., other			
Model input	Data source(s)	Distribution	Parameters
Reported illnesses	Number of illnesses due to *Vibrio spp.* other than *V. alginolyticus, V. vulnificus*, and *V. parahaemolyticus* reported to CDC's Cholera and Other Vibrio Illness Surveillance (COVIS) System (2008–2014) (*41)*. Because of an apparent trend over time, linear regression was used to estimate the projected illness for reference year 2014. The uncertainty around the estimated illness was based on the residuals from linear regression (see Appendix 2 for more information).	Empirical	By year (2008–2014): 141, 203, 221, 249, 206, 240, 282
Population adjustment (year)	Population ratios applied to each year from 2008–2014 based on US Census population estimates (*2*).	Degenerate	Adjustment by year (2008–2014): 1.05, 1.04, 1.03, 1.02, 1.01, 1.0, 1.0

Pathogen: *Vibrio* spp., other			
Model input	Data source(s)	Distribution	Parameters
Underreporting	Passive surveillance multiplier used to adjust for underreporting (*3*).	PERT	Low, modal, high values: 0.9, 1.1, 1.3
Underdiagnosis (for number of illnesses)			
Percent severe	Assumed to be a similar illness to non-typhoidal *Salmonella* infection.	PERT	Low, modal, high values: 0.35, 0.45, 0.71
Medical care seeking (severe)	Proportion (and 95% CI) of survey respondents with bloody diarrhea who sought medical care ,from FoodNet Population Surveys (2000–2001, 2002–2003, 2006–2007) (*3*)	PERT	Low, modal, high values: 0.19, 0.35, 0.51
Medical care seeking (mild)	Proportion (and 95% CI) of survey respondents with non-bloody diarrhea who sought medical care, from FoodNet Population Surveys (2000–2001, 2002–2003, 2006–2007) (*3*).	PERT	Low, modal, high values: 0.15, 0.18, 0.20
Specimen submission (severe)	Proportion (and 95% CI) of survey respondents who submitted a fecal specimen among persons with bloody diarrhea who sought medical care from FoodNet Population Surveys (2000–2001, 2002–2003, 2006–2007) (*3*).,	PERT	Low, modal, high values: 0.11, 0.36, 0.62
Specimen submission (mild)	Proportion (and 95% CI) of survey respondents who submitted a fecal specimen among persons with a non-bloody diarrhea who sought medical care, from FoodNet Population Surveys (2000–2001, 2002–2003, 2006–2007) (*3*).	PERT	Low, modal, high values: 0.12, 0.19, 0.25
Laboratory testing	Proportion of clinical laboratories routinely testing fecal samples for *Vibrio* spp., from the FoodNet Laboratory Survey (*4*).	PERT	Low, modal, high values: 0.41, 0.51, 0.61
Test sensitivity	Proportion of clinical laboratories using appropriate diagnostic tests to test fecal samples for *Vibrio* spp., from the FoodNet Laboratory Survey (*4*).	PERT	Low, modal, high values: 0.21, 0.28, 0.37
Proportion with a treat-and-release ED visit	Could not calculate because of a poor fit between International Classification of Diseases, 9th Edition, Clinical Modification (ICD-9-CM) codes and infections by individual *Vibrio* species		
Proportion hospitalized	Proportion of case-patients with *Vibrio,* other infection reported to COVIS who were hospitalized (2008–2014).	Empirical	By year (2008–2014): 0.48, 0.45, 0.38, 0.36, 0.44, 0.46, 0.41
Proportion who died	Proportion of case-patients with *Vibrio,* other infection reported to COVIS who died (2008–2014).	Empirical	By year (2008–2014): 0.04, 0.05, 0.03, 0.03, 0.04, 0.03, 0.04
Underdiagnosis (ED visits, hospitalizations, deaths)	Number of emergency department visits, hospitalizations, and deaths doubled to account for underdiagnosis.	PERT	Low, modal, high values: 1, 2, 3
Proportion travel-related	Based on proportion of case-patients with *Vibrio,* other infection reported to COVIS who acquired the infection while traveling outside the United States in the 7 d before illness onset (2008–2014).	PERT	Low, modal, high values: 0.0992, 0.1339, 0.2275
Proportion waterborne	Structured expert judgment estimate for *Vibrio,* other infection (*9*).	Empirical	2.5th percentile, median, mean, 97.5th percentile: 0, 0.01, 0.02, 0.23
Cost of treat-and-release ED visits	Could not calculate because of a poor fit between ICD-9-CM codes and infections by individual *Vibrio* species.		
Cost of hospitalizations	Could not calculate because of a poor fit between ICD-9-CM codes and infections by individual *Vibrio* species.		
Pathogen-specific limitations and discussion	Could not calculate costs because of poor ICD-9 code fit.		

* A note on the descriptions of the distributions used here: The term “empirical” used here (as in Empirical Cumulative Density Function [ECDF]) refers to using a “finite sample” to construct a distribution to approximate the true underlying distribution/theoretical distribution. In this sense, bootstrapping observed data by simulation and simulating pseudodata for the burden outcomes are both empirical. “Degenerate” in probability terms means a constant here, in contrast to a random variable. In other words, it refers to a random variable that has a single possible value (a constant with probability 1). NA, not applicable; PERT, Program Evaluation and Review Technique.

Appendix 1 Table 2. Number of cases of illness reported to CDC's Foodborne Diseases Active Surveillance Network (FoodNet) by pathogen, year, and FoodNet site

		FoodNet site									
Pathogen	Year	CA	CO	CT	GA	MD	MN	NM	NY	OR	TN
Campylobacter spp.	2012	1189	414	600	1058	662	1192	399	614	907	660
Campylobacter spp.	2013	1076	344	694	983	708	1190	415	673	870	741
Campylobacter spp.	2014	1040	330	812	946	729	1082	431	708	906	679
Campylobacter spp.	2015	1245	451	783	1090	772	1411	463	636	884	759
Cryptosporidium spp.	2012	48	26	41	275	83	340	94	50	230	71
Cryptosporidium spp.	2013	46	51	38	302	65	324	47	79	177	88
Cryptosporidium spp.	2014	32	26	44	269	76	337	80	88	113	124
Cryptosporidium spp.	2015	63	69	82	406	103	319	54	99	197	266
E. coli, STEC O157	2012	39	37	19	34	33	123	15	69	95	69
E. coli, STEC O157	2013	64	26	32	48	28	144	11	42	105	54
E. coli, STEC O157	2014	51	19	17	25	20	128	14	29	72	69
E. coli, STEC O157	2015	47	38	27	27	24	114	7	27	108	47
E. coli, STEC non-O157	2012	23	42	31	91	37	110	41	43	72	50
E. coli, STEC non-O157	2013	26	70	36	66	34	127	18	45	58	66
E. coli, STEC non-O157	2014	57	67	40	64	44	164	33	48	86	72
E. coli, STEC non-O157	2015	114	69	53	76	53	110	26	57	107	116
Salmonella spp.	2012	484	264	455	2681	906	781	327	501	387	1057
Salmonella spp.	2013	586	323	446	2288	829	811	350	451	364	859
Salmonella spp.	2014	618	332	464	2247	897	722	327	503	376	953
Salmonella spp.	2015	601	316	450	2113	935	974	429	486	518	897
Shigella spp.	2012	215	56	44	666	181	391	96	211	78	203
Shigella spp	2013	193	90	59	907	105	133	61	68	52	665
Shigella spp	2014	345	55	65	1038	248	93	63	42	45	780
Shigella spp	2015	299	66	60	1301	198	292	72	43	106	208

Appendix 1 Table 3. Proportion of case-patients hospitalized, from CDC's Foodborne Diseases Active Surveillance Network (FoodNet) by pathogen, year, and FoodNet site

		FoodNet sites									
Pathogen	Year	CA	CO	CT	GA	MD	MN	NM	NY	OR	TN
Campylobacter spp.	2012	0.1490	0.1418	0.1760	0.2375	0.1755	0.2097	0.2532	0.1800	0.0907	0.2931
Campylobacter spp.	2013	0.1157	0.1462	0.1879	0.2810	0.2193	0.1630	0.2029	0.1985	0.0820	0.3175
Campylobacter spp.	2014	0.1360	0.1311	0.2145	0.2804	0.1886	0.1867	0.2266	0.2201	0.1199	0.3002
Campylobacter spp.	2015	0.1165	0.1496	0.1826	0.2922	0.2011	0.1979	0.2078	0.2208	0.1017	0.3225
Cryptosporidium spp.	2012	0.1795	0.2000	0.1220	0.3187	0.4691	0.1088	0.2903	0.1000	0.0437	0.2941
Cryptosporidium spp.	2013	0.1282	0.1765	0.0263	0.3199	0.3860	0.1296	0.3043	0.1899	0.0739	0.3125
Cryptosporidium spp.	2014	0.2069	0.2308	0.1591	0.3529	0.2917	0.1335	0.1125	0.1494	0.0545	0.1933
Cryptosporidium spp.	2015	0.2931	0.1159	0.1481	0.2926	0.2059	0.0909	0.1296	0.1429	0.0663	0.1587
E. coli, STEC O157	2012	0.2821	0.4167	0.3158	0.4706	0.4242	0.3171	0.4000	0.3676	0.3158	0.4478
E. coli, STEC O157	2013	0.3548	0.3077	0.3438	0.2766	0.3571	0.3542	0.3636	0.5476	0.4571	0.4118
E. coli, STEC O157	2014	0.3265	0.2632	0.5882	0.3200	0.4500	0.2969	0.4286	0.3793	0.2917	0.4559
E. coli, STEC O157	2015	0.3696	0.3421	0.4074	0.2800	0.4583	0.3158	0.4286	0.6296	0.4206	0.4419
E. coli, STEC non-O157	2012	0.1500	0.1220	0.2581	0.0769	0.2162	0.1455	0.3171	0.2326	0.1389	0.1042
E. coli, STEC non-O157	2013	0.0833	0.0571	0.1714	0.0606	0.1250	0.1654	0.3333	0.2667	0.0877	0.1563
E. coli, STEC non-O157	2014	0.0800	0.0758	0.1282	0.0702	0.1905	0.1707	0.2121	0.1250	0.1279	0.3676
E. coli, STEC non-O157	2015	0.1273	0.1471	0.1698	0.1757	0.1132	0.2545	0.1923	0.1754	0.1028	0.1415
Salmonella spp.	2012	0.2249	0.2727	0.2539	0.2986	0.3464	0.2586	0.3137	0.3353	0.2506	0.3834
Salmonella spp.	2013	0.2246	0.2755	0.2320	0.2896	0.3350	0.2762	0.2607	0.3073	0.2149	0.3522
Salmonella spp.	2014	0.2147	0.2515	0.3218	0.3169	0.3546	0.2645	0.2936	0.3153	0.2139	0.3297
Salmonella spp.	2015	0.2255	0.2283	0.2978	0.2938	0.2971	0.2710	0.3380	0.2934	0.2058	0.3184
Shigella spp.	2012	0.2100	0.1964	0.2727	0.2606	0.2652	0.1432	0.3053	0.2559	0.1538	0.3094
Shigella spp	2013	0.2640	0.1778	0.2712	0.1674	0.4043	0.2556	0.2131	0.2794	0.2885	0.1866
Shigella spp	2014	0.3526	0.3091	0.2462	0.2133	0.2544	0.2151	0.3016	0.1190	0.1778	0.1397
Shigella spp	2015	0.2955	0.2576	0.2167	0.2311	0.2974	0.2158	0.1806	0.3256	0.3113	0.2402

Appendix 1 Table 4. Proportion of case-patients who died, from CDC's Foodborne Diseases Active Surveillance Network (FoodNet) by pathogen, year, and FoodNet site

		FoodNet sites									
Pathogen	Year	CA	CO	CT	GA	MD	MN	NM	NY	OR	TN
Campylobacter spp.	2012	0.0000	0.0024	0.0000	0.0042	0.0016	0.0034	0.0026	0.0016	0.0000	0.0016
Campylobacter spp.	2013	0.0000	0.0000	0.0000	0.0083	0.0015	0.0042	0.0000	0.0045	0.0011	0.0014
Campylobacter spp.	2014	0.0000	0.0000	0.0000	0.0012	0.0000	0.0046	0.0023	0.0028	0.0033	0.0030
Campylobacter spp.	2015	0.0082	0.0022	0.0013	0.0080	0.0013	0.0064	0.0043	0.0016	0.0011	0.0040
Cryptosporidium spp.	2012	0.0000	0.0000	0.0000	0.0000	0.0366	0.0029	0.0108	0.0000	0.0043	0.0000
Cryptosporidium spp.	2013	0.0000	0.0196	0.0000	0.0066	0.0000	0.0031	0.0000	0.0000	0.0000	0.0000
Cryptosporidium spp.	2014	0.0000	0.0000	0.0000	0.0040	0.0000	0.0059	0.0000	0.0000	0.0000	0.0082
Cryptosporidium spp.	2015	0.0000	0.0145	0.0000	0.0076	0.0000	0.0000	0.0000	0.0000	0.0051	0.0038
E. coli, STEC O157	2012	0.0000	0.0000	0.0000	0.0323	0.0000	0.0000	0.0000	0.0000	0.0000	0.0000
E. coli, STEC O157	2013	0.0000	0.0000	0.0000	0.0000	0.0000	0.0000	0.0000	0.0000	0.0190	0.0000
E. coli, STEC O157	2014	0.0000	0.0000	0.0000	0.0000	0.0000	0.0078	0.0000	0.0000	0.0139	0.0145
E. coli, STEC O157	2015	0.0000	0.0000	0.0370	0.0000	0.0000	0.0000	0.1429	0.0000	0.0093	0.0000
E. coli, STEC non-O157	2012	0.0476	0.0000	0.0000	0.0000	0.0000	0.0000	0.0000	0.0000	0.0000	0.0000
E. coli, STEC non-O157	2013	0.0000	0.0000	0.0000	0.0000	0.0000	0.0079	0.0000	0.0000	0.0000	0.0000
E. coli, STEC non-O157	2014	0.0000	0.0000	0.0000	0.0000	0.0000	0.0000	0.0000	0.0208	0.0000	0.0000
E. coli, STEC non-O157	2015	0.0000	0.0000	0.0000	0.0000	0.0000	0.0000	0.0000	0.0000	0.0093	0.0000
Salmonella spp.	2012	0.0022	0.0038	0.0066	0.0035	0.0034	0.0038	0.0031	0.0080	0.0103	0.0030
Salmonella spp.	2013	0.0036	0.0093	0.0090	0.0026	0.0062	0.0012	0.0000	0.0044	0.0055	0.0061
Salmonella spp.	2014	0.0051	0.0091	0.0043	0.0049	0.0033	0.0042	0.0061	0.0020	0.0027	0.0043
Salmonella spp.	2015	0.0018	0.0032	0.0044	0.0059	0.0053	0.0010	0.0140	0.0000	0.0039	0.0023
Shigella spp.	2012	0.0000	0.0000	0.0000	0.0017	0.0055	0.0026	0.0000	0.0000	0.0000	0.0000
Shigella spp	2013	0.0000	0.0000	0.0000	0.0011	0.0000	0.0000	0.0000	0.0000	0.0000	0.0032
Shigella spp	2014	0.0032	0.0000	0.0000	0.0031	0.0000	0.0000	0.0000	0.0000	0.0000	0.0000
Shigella spp	2015	0.0000	0.0000	0.0000	0.0008	0.0000	0.0000	0.0000	0.0000	0.0000	0.0000

References

1. Marder EP, Griffin PM, Cieslak PR, Dunn J, Hurd S, Jervis R, et al. Preliminary incidence and trends of infections with pathogens transmitted commonly through food—Foodborne Diseases Active Surveillance Network, 10 US Sites, 2006–2017. MMWR Morb Mortal Wkly Rep. 2018;67:324–8. PubMed https://doi.org/10.15585/mmwr.mm6711a3

2. US Census Bureau Population Division. Annual estimates of the resident population: April 1, 2010 to July 1, 2019 [cited 2020 Sep 24]. https://www2.census.gov/programs-surveys/popest/tables/2010-2019/state/totals/nst-est2019-01.xlsx

3. Scallan E, Hoekstra RM, Angulo FJ, Tauxe RV, Widdowson MA, Roy SL, et al. Foodborne illness acquired in the United States—major pathogens. Emerg Infect Dis. 2011;17:7–15. PubMed https://doi.org/10.3201/eid1701.P11101

4. Voetsch AC, Angulo FJ, Rabatsky-Ehr T, Shallow S, Cassidy M, Thomas SM, et al.; Emerging Infections Program FoodNet Working Group. Laboratory practices for stool-specimen culture for bacterial pathogens, including *Escherichia coli* O157:H7, in the FoodNet sites, 1995–2000. Clin Infect Dis. 2004;38(Suppl 3):S190–7. PubMed https://doi.org/10.1086/381586

5. Geissler AL, Bustos Carrillo F, Swanson K, Patrick ME, Fullerton KE, Bennett C, et al. Increasing *Campylobacter* infections, outbreaks, and antimicrobial resistance in the United States, 2004–2012. Clin Infect Dis. 2017;65:1624–31. PubMed https://doi.org/10.1093/cid/cix624

6. Gu W, Dutta V, Patrick M, Bruce BB, Geissler A, Huang J, et al. Statistical adjustment of culture-independent diagnostic tests for trend analysis in the Foodborne Diseases Active Surveillance Network (FoodNet), USA. Int J Epidemiol. 2018;47:1613–22. PubMed

7. Chalker RB, Blaser MJ. A review of human salmonellosis: III. Magnitude of *Salmonella* infection in the United States. Rev Infect Dis. 1988;10:111–24. PubMed https://doi.org/10.1093/clinids/10.1.111

8. Voetsch AC, Van Gilder TJ, Angulo FJ, Farley MM, Shallow S, Marcus R, et al.; Emerging Infections Program FoodNet Working Group. FoodNet estimate of the burden of illness caused by nontyphoidal *Salmonella* infections in the United States. Clin Infect Dis. 2004;38(Suppl 3):S127–34. PubMed https://doi.org/10.1086/381578

9. Beshearse E, Bruce BB, Nane GF, Cooke RM, Aspinall W, Hald T, et al. Using structured expert judgment for attribution of foodborne and waterborne illnesses to comprehensive transmission pathways, United States. Emerg Infect Dis. 2021 Jan [in press]. https://doi.org/10.3201/eid2701.200316

10. Adam EA, Collier SA, Fullerton KE, Gargano JW, Beach MJ. Prevalence and direct costs of emergency department visits and hospitalizations for selected diseases that can be transmitted by water, United States. J Water Health. 2017;15:673–83. PubMed https://doi.org/10.2166/wh.2017.083

11. Painter JE, Gargano JW, Collier SA, Yoder JS; Centers for Disease Control and Prevention. Giardiasis surveillance—United States, 2011–2012. MMWR Suppl. 2015;64:15–25.

12. Huang DB, White AC. An updated review on *Cryptosporidium* and *Giardia*. Gastroenterol Clin North Am. 2006;35:291–314, viii. PubMed https://doi.org/10.1016/j.gtc.2006.03.006

13. Reses HE, Gargano JW, Liang JL, Cronquist A, Smith K, Collier SA, et al. Risk factors for sporadic *Giardia* infection in the USA: a case-control study in Colorado and Minnesota. Epidemiol Infect. 2018;146:1071–8. PubMed https://doi.org/10.1017/S0950268818001073

14. Beer KD, Collier SA, Du F, Gargano JW. Giardiasis diagnosis and treatment practices among commercially insured persons in the United States. Clin Infect Dis. 2017;64:1244–50. PubMed https://doi.org/10.1093/cid/cix138

15. Hennessy TW, Marcus R, Deneen V, Reddy S, Vugia D, Townes J, et al.; Emerging Infections Program FoodNet Working Group. Survey of physician diagnostic practices for patients with acute diarrhea: clinical and public health implications. Clin Infect Dis. 2004;38(Suppl 3):S203–11. PubMed https://doi.org/10.1086/381588

16. Polage CR, Stoddard GJ, Rolfs RT, Petti CA. Physician use of parasite tests in the United States from 1997 to 2006 and in a Utah *Cryptosporidium* outbreak in 2007. J Clin Microbiol. 2011;49:591–6. PubMed https://doi.org/10.1128/JCM.01806-10

17. Centers for Disease Control and Prevention. Legionnaires' disease surveillance summary report, 2014–2015; 2018 [cited 2018 Nov 14]. https://www.cdc.gov/legionella/health-depts/surv-reporting/2014-15-surv-report-508.pdf

18. Dooling KL, Toews KA, Hicks LA, Garrison LE, Bachaus B, Zansky S, et al. Active bacterial core surveillance for legionellosis—United States, 2011–2013. MMWR Morb Mortal Wkly Rep. 2015;64:1190–3. PubMed https://doi.org/10.15585/mmwr.mm6442a2

19. Decker BK, Harris PL, Muder RR, Hong JH, Singh N, Sonel AF, et al. Improving the diagnosis of *Legionella* pneumonia within a healthcare system through a systematic consultation and testing program. Ann Am Thorac Soc. 2016;13:1289–93. PubMed https://doi.org/10.1513/AnnalsATS.201510-715BC

20. Helbig JH, Uldum SA, Lück PC, Harrison TG. Detection of *Legionella pneumophila* antigen in urine samples by the BinaxNOW immunochromatographic assay and comparison with both Binax Legionella Urinary Enzyme Immunoassay (EIA) and Biotest Legionella Urin Antigen EIA. J Med Microbiol. 2001;50:509–16. PubMed https://doi.org/10.1099/0022-1317-50-6-509

21. Valcina O, Pūle D, Lucenko I, Krastina D, Šteingolde Ž, Krūmina A, et al. *Legionella pneumophila* seropositivity-associated factors in Latvian blood donors. Int J Environ Res Public Health. 2015;13:58. https://doi.org/10.3390/ijerph13010058

22. Strollo SE, Adjemian J, Adjemian MK, Prevots DR. The burden of pulmonary nontuberculous mycobacterial disease in the United States. Ann Am Thorac Soc. 2015;12:1458–64. PubMed https://doi.org/10.1513/AnnalsATS.201503-173OC

23. Gargano JW, Adam EA, Collier SA, Fullerton KE, Feinman SJ, Beach MJ. Mortality from selected diseases that can be transmitted by water—United States, 2003–2009. J Water Health. 2017;15:438–50. PubMed https://doi.org/10.2166/wh.2017.301

24. Donohue MJ. Increasing nontuberculous mycobacteria reporting rates and species diversity identified in clinical laboratory reports. BMC Infect Dis. 2018;18:163. PubMed https://doi.org/10.1186/s12879-018-3043-7

25. Grytdal SP, DeBess E, Lee LE, Blythe D, Ryan P, Biggs C, et al. Incidence of norovirus and other viral pathogens that cause acute gastroenteritis (AGE) among Kaiser Permanente member populations in the United States, 2012–2013. PLoS One. 2016;11:e0148395. PubMed https://doi.org/10.1371/journal.pone.0148395

26. Hall AJ, Rosenthal M, Gregoricus N, Greene SA, Ferguson J, Henao OL, et al. Incidence of acute gastroenteritis and role of norovirus, Georgia, USA, 2004–2005. Emerg Infect Dis. 2011;17:1381–8. PubMed https://doi.org/10.3201/eid1708.101533

27. Gastañaduy PA, Hall AJ, Curns AT, Parashar UD, Lopman BA. Burden of norovirus gastroenteritis in the ambulatory setting—United States, 2001–2009. J Infect Dis. 2013;207:1058–65. PubMed https://doi.org/10.1093/infdis/jis942

28. Lopman BA, Hall AJ, Curns AT, Parashar UD. Increasing rates of gastroenteritis hospital discharges in US adults and the contribution of norovirus, 1996–2007. Clin Infect Dis. 2011;52:466–74. PubMed https://doi.org/10.1093/cid/ciq163

29. Hall AJ, Lopman BA, Payne DC, Patel MM, Gastañaduy PA, Vinjé J, et al. Norovirus disease in the United States. Emerg Infect Dis. 2013;19:1198–205. PubMed https://doi.org/10.3201/eid1908.130465

30. Collier SA, Wade TJ, Sams EA, Hlavsa MC, Dufour AP, Beach MJ. Swimming in the USA: beachgoer characteristics and health outcomes at US marine and freshwater beaches. J Water Health. 2015;13:531–43. PubMed https://doi.org/10.2166/wh.2014.095

31. Roland PS, Stroman DW. Microbiology of acute otitis externa. Laryngoscope. 2002;112:1166–77. PubMed https://doi.org/10.1097/00005537-200207000-00005

32. van Asperen IA, de Rover CM, Schijven JF, Oetomo SB, Schellekens JF, van Leeuwen NJ, et al. Risk of otitis externa after swimming in recreational fresh water lakes containing *Pseudomonas aeruginosa*. BMJ. 1995;311:1407–10. PubMed https://doi.org/10.1136/bmj.311.7017.1407

33. Hajjartabar M. Poor-quality water in swimming pools associated with a substantial risk of otitis externa due to *Pseudomonas aeruginosa*. Water Sci Technol. 2004;50:63–7. PubMed https://doi.org/10.2166/wst.2004.0020

34. Wade TJ, Sams EA, Beach MJ, Collier SA, Dufour AP. The incidence and health burden of earaches attributable to recreational swimming in natural waters: a prospective cohort study. Environ Health. 2013;12:67. PubMed https://doi.org/10.1186/1476-069X-12-67

35. Centers for Disease Control and Prevention (CDC). Estimated burden of acute otitis externa—United States, 2003–2007. MMWR Morb Mortal Wkly Rep. 2011;60:605–9. PubMed

36. Collier SA, Hlavsa MC, Piercefield EW, Beach MJ. Antimicrobial and analgesic prescribing patterns for acute otitis externa, 2004–2010. Otolaryngol Head Neck Surg. 2013;148:128–34. PubMed https://doi.org/10.1177/0194599812467000

37. Voetsch AC, Kennedy MH, Keene WE, Smith KE, Rabatsky-Ehr T, Zansky S, et al. Risk factors for sporadic Shiga toxin-producing *Escherichia coli* O157 infections in FoodNet sites, 1999–2000. Epidemiol Infect. 2007;135:993–1000. PubMed https://doi.org/10.1017/S0950268806007564

38. Hoefer D, Hurd S, Medus C, Cronquist A, Hanna S, Hatch J, et al.; Emerging Infections Program FoodNet Working Group. Laboratory practices for the identification of Shiga toxin-producing *Escherichia coli* in

the United States, FoodNet sites, 2007. Foodborne Pathog Dis. 2011;8:555–60. PubMed https://doi.org/10.1089/fpd.2010.0764

39. Gould LH, Mody RK, Ong KL, Clogher P, Cronquist AB, Garman KN, et al.; Emerging Infections Program Foodnet Working Group. Increased recognition of non-O157 Shiga toxin-producing *Escherichia coli* infections in the United States during 2000–2010: epidemiologic features and comparison with *E. coli* O157 infections. Foodborne Pathog Dis. 2013;10:453–60. PubMed https://doi.org/10.1089/fpd.2012.1401

40. Hedican EB, Medus C, Besser JM, Juni BA, Koziol B, Taylor C, et al. Characteristics of O157 versus non-O157 Shiga toxin-producing *Escherichia coli* infections in Minnesota, 2000–2006. Clin Infect Dis. 2009;49:358–64. PubMed https://doi.org/10.1086/600302

41. Centers for Disease Control and Prevention. National Enteric Disease Surveillance: COVIS Annual Summary, 2014; 2016 [cited 2018 Nov 14]. https://www.cdc.gov/nationalsurveillance/pdfs/covis-annual-summary-2014-508c.pdf

42. Cockerill FR III, Wilson JW, Vetter EA, Goodman KM, Torgerson CA, Harmsen WS, et al. Optimal testing parameters for blood cultures. Clin Infect Dis. 2004;38:1724–30. PubMed https://doi.org/10.1086/421087

43. Lee A, Mirrett S, Reller LB, Weinstein MP. Detection of bloodstream infections in adults: how many blood cultures are needed? J Clin Microbiol. 2007;45:3546–8. PubMed https://doi.org/10.1128/JCM.01555-07
