# Supplementary material for: Estimate of Burden and Direct Healthcare Cost of Infectious Waterborne Disease in the United States
Source: Emerg Infect Dis. 2021 Jan;27(1):140–9. doi: 10.3201/eid2701.190676 (PMC7774540; doi:10.3201/eid2701.190676)
Supplement: Appendix 2 — Model types used to make estimates for study of the burden and direct healthcare cost of infectious waterborne disease in the United States. [file 19-0676-Techapp-s2.pdf]

Article DOI: https://doi.org/10.3201/eid2701.190676

Estimate of Burden and Direct Healthcare Cost of Infectious Waterborne Disease in the United States

Appendix 2

Model Types Used to Make Estimates

The process of estimating the burden of waterborne illness requires the use of disparate data sources and making subjective decisions on how to combine them. Briefly, after we identified our illnesses of interest, we combined data from available data sources (surveillance data systems, administrative data, or data from the literature) and applied multipliers to account for population standardization, underreporting, underdiagnosis, proportion domestically acquired, and proportion attributable to waterborne transmission. For pathogens with surveillance data, we adapted an approach laid out previously to estimate the burden of foodborne illness (*1*), with some modifications and differences, detailed in this appendix. For pathogens with administrative or literature data only, we developed new models to estimate the burden of waterborne illness. The summary statistics are based on distributions constructed from Monte Carlo simulation records. We report the mean and 95% credible interval (CrI), a range that covers 95% of the sample.

Burden Outcomes

We used the estimated annual total number of illnesses, hospitalizations, deaths, emergency department (ED) visits, total health care cost for hospitalizations, and total health care cost for ED visits to measure the burden of waterborne diseases in the United States.

Model Structures

We used 3 broad model types to estimate the burden outcomes, except health care cost burdens, for 17 known waterborne pathogens. Variations exist within each model type depending

on the pathogen, the diagnostic test type, severity of the disease, availability of input data, and choices made on multiplier values. Details on the variations by pathogen are available in Appendix 1.

Model type A was used for surveillance data. This model scales counts of laboratory-confirmed (reported) illnesses up to an estimated number of illnesses, accounting for both underreporting and underdiagnosis factors that contribute to illnesses not being reported to surveillance systems. This model was applied to both active and passive surveillance data (Appendix 2 Table).

Model type B was used for administrative data. This model scales hospitalization counts reported in administrative datasets up to an estimated number of illness, accounting for both hospitalization rate and underreporting and underdiagnosis factors that contribute to an illness not being seen in a hospital or reported to hospital discharge databases (Appendix 2 Table).

Model type C was used for publication-based data. This model scales populations at risk down to an estimated number of illnesses using publication reported incidence rates (Appendix 2 Table).

Model Type A: Burden Estimate for Pathogens Reported from Surveillance Systems

Model inputs (illnesses, hospitalizations, and deaths) were assigned distributions using previously defined methods (*1*). For pathogens reported in the active surveillance system (FoodNet) (*2*), data from different sites or years were treated as representatives from distinct populations. We chose to treat, for example, FoodNet confirmed case counts from 10 sites over 4 years (2012–2015) as representing 40 distinct population means. Each population contributes to the empirical distribution with equal probability. For pathogens reported in the passive surveillance systems, linear regression was applied to fit multiyear (2008–2014) national data and to estimate the average burden count for the reference year 2014. Residuals from all 7 years were randomly sampled with equal chance and then used in the calculation of the uncertainty of the predicted count, simulating the distribution of reported count.

All model inputs are multiplicative. Each multiplier either expands or contracts the observed/reported burden counts to produce the final burden estimate. We assume all multipliers in model type A to be mutually independent except for the ones associated with the

underdiagnosis of illness, where the multipliers associated with care-seeking and specimen submission rate depend on the severity of cases.

The distributions of model outputs were obtained via Monte Carlo simulation. During each simulation run, a random sample was drawn from the theoretical/empirical distribution of each model input; then they were multiplied sequentially depending on their positions in the model. The final product of all factors yielded the burden estimate. The empirical distribution pooled from a large number of simulated records (100,000 iterations) allowed us to estimate the uncertainty of the burden outcomes.

Only a fraction of cases whose records passed a series of stages in the reporting process could be seen in our surveillance system. Each multiplier value refers to the proportion of case records advancing to the next stage (e.g., the proportion of patients seeking medical care), and these multiplier values are all <1. To estimate the burden of illness, we use the reciprocal of these multiplier values, called expansive factors, to scale up the number of reported cases from the surveillance system (Appendix 2 Figure 1 and Figure 2, panel B). Appendix 2 Figure 1 describes the modeling process of scaling up reported confirmed cases by surveillance system up (model type A) in a mathematical format. The order of the multiplication does not matter, as the factors are commutative. The diagram shows 9 primary model outputs, identified in the box in the middle and obtained by inclusion of elements from vectors (column [1 or H or D] and row [1 or Dom or W]). For example, a combination of choosing D, Dom, and W yields the output for domestic waterborne deaths. Each of these factors is either a random variable, following an empirical distribution constructed from observed or estimated data or a parametric distribution, or a constant, such as the year adjustment factor to the 2014 population size. As illustrated in Appendix 2 Figures 2 and 3, the central location and spread of each model output reflects not only the multiplicative effect of its components but also the cumulative and joint effect of their uncertainties.

For all multipliers except the water attribution rate, we assumed the same distribution properties as those in the foodborne burden paper (*1*). We updated the distribution parameters whenever new data or information were available. For the waterborne attribution proportion, a calibrated and synthesized distribution for each pathogen was obtained from elicitation results of a panel of experts (*3*). The same assumptions about multiplier distributions were made among all

model types (A, B, and C). The underdiagnosis/underreporting factors for ED visits, hospitalizations, and deaths were set as beta/PERT distributions (*4*) with values of (min, mode, max)= (1, 2, 3). This rule was applied to all pathogens in the surveillance systems unless otherwise stated. Details of the choices made to define the distributions of model inputs by pathogen are available in Appendix 1.

Appendix 2 Figures 2 and 3 demonstrate the distributions involved in constructing estimates for *Shigella*, including the annual illness estimate (Appendix 2 Figure 2, panel A), the underdiagnosis multiplier (Appendix 2 Figure 2, panel B) and the hospitalization estimate (Appendix 2 Figure 3). The empirical and discrete nature of the source data is apparent in the first panel of Appendix 2 Figure 2, panel A. The right skewness in the water attribution rate dominates the distribution of the domestic waterborne illness. As shown in Appendix 2 Figure 2, panel B, a series of multipliers contributed to illnesses not being seen or verified or reported. These multipliers expanded the laboratory reported case counts in a multiplicative fashion and their impacts were passed onto the combined underdiagnosis multiplier in the main model (Appendix 2 Figure 2, panel A). The hospitalization estimate, as shown in Appendix 2 Figure 3, was similar to the illness estimate.

Model Type B: Burden Estimate for Pathogens/Data Reported from Administrative Systems and ED Visits for All Pathogens

Pathogens for which model type B was used did not have data available from national surveillance systems. Instead, data from the Agency for Healthcare Research and Quality Health Care Utilization Project's National Inpatient Sample (HCUP NIS) (*5*) and National Emergency Department Sample (HCUP NEDS) (*6*) were used. The NIS is the largest publicly available US hospital discharge database that includes all sources of payment (i.e., private insurance, public insurance, and the uninsured). HCUP NIS is a complex sample survey that produces weighted national estimates from a stratified sample of about 20% of hospital stays from community hospitals in the United States. Similarly, HCUP NEDS is a complex sample survey that produces weighted national estimates of emergency department visits. Appendix 2 Figure 4 shows the estimation steps for model type B pathogens. Each of these factors is either a random variable, following a parametric distribution (e.g., normal distribution or beta/PERT distribution), or a constant (year adjustment factor). Unlike pathogens in surveillance systems (model type A), here

hospitalization counts served as the initial model input. They were scaled up to estimate the number of illnesses by dividing by the hospitalization rate.

For ED visits, hospitalizations, and death counts reported in HCUP datasets, we assumed a mixture of 3 normal distributions with equal probability, with each year of data providing the parameters of a mixture component. Each individual year represented 1 normal population. The associated parameter mean was taken from the weighted nationwide frequency count and the standard deviation was calculated from the lower and upper limits assuming a normal distribution was used in the confidence interval construction.

The death counts were derived from 2 sources. The total death count is the sum of in-hospital deaths and out-of-hospital deaths, as described by Gargano et al. (*7*). In-hospital deaths were obtained using the number of hospitalizations for a particular illness in the HCUP NIS database that ended in death. Out-of-hospital deaths were obtained from out-of-hospital deaths reported for a particular illness in the National Vital Statistics System (NVSS) (*8*), which contains information on all death certificates filed in the United States. No uncertainty was reported for the out-of-hospital death count. Here we used it as a constant rather than a distribution. The model outputs and the distributions were quantified via Monte Carlo simulation.

Special treatments were employed in the simulation algorithms to ensure that the biological or clinical constraints of the model outputs were met. First, when a negative number occurred in the simulation under a normal distribution, it was replaced with zero. Second, for pathogens with high hospitalization rates ($\geq$75%), the 3 underdiagnosis factors (for illnesses, hospitalizations, and deaths) were set to be the same for each simulated record. This treatment ensured that the number of illness was greater than the number of hospitalizations and the number of deaths, true not only for the mean value but also for each simulated individual record.

The multiplicative impact of each factor on the final burden estimate was illustrated in Appendix 2 Figure 5. Details of the choices made on the multipliers and their parameters are provided in Appendix 1.

Appendix 2 Figure 5 demonstrates the distributions involved in constructing estimates of annual illnesses for *Pseudomonas* pneumonia. As shown previously, the hospitalization was a mixture of 3 normal distributions with variable mean values. Although the annual illness was

multimodal, the other multipliers followed 1-mode beta/PERT-distribution, spreading narrowly around their modes. The resulting smoothed distribution of domestically acquired waterborne illness was unimodal. The estimations for hospitalizations, deaths, and ED visits were modeled in a similar way except that the hospitalization rate component (the third panel) was removed from the equation.

Model Type C: Burden Estimate for Pathogen Reported from Literature Data

Model type C was used for 1 pathogen, norovirus. For norovirus, instead of using acute gastrointestinal illness (AGI) as a starting point for the estimate (*1*), we used incidence estimates (already adjusted for underdiagnosis) from 2 studies (*9,10*). The Hall et al. study (*9*) was conducted at 1 site of the Kaiser Permanente health care system. The Grytdal et al. study (*10*) was conducted at 2 additional sites of the Kaiser Permanente health care system. To combine the studies, the reported 3-number summary statistics (mean, lower, and upper limit) for incidence rate at each site were fit to a 4-parameter PERT distribution with the variation parameter fixed, while minimizing the overall distance between the 3 summary statistics and the model predicted values. The process was repeated for different values of the variation parameter. The corresponding parameter combination under the best fit, verified by subject matter experts via visual examinations, was assigned as the PERT distribution parameters for that site. The sampling distribution for the annual incidence rate was a mixture of the 3 beta distributions with 1 distribution representing 1 site. Each site had an equal probability of being drawn. We chose beta/PERT distribution to describe the incidence rate for the following reasons. First, the reported confidence intervals were asymmetric, making the normal distribution not immediately applicable. Second, the original datasets used to produce CIs in the publications were not available to us. Third, the beta distribution family has the capacity to accommodate left-skewed, right-skewed, and symmetric confidence intervals or distributions. Fourth, incidence rate takes values on a range with an upper and lower bound. The generalized beta/PERT distribution has the flexibility to set a range on incidence rates. In addition, we selected a different value from the default setup for the variation parameter of the PERT distribution, the same strategy used in the previous foodborne burden paper (*11*) because it gave us a fit with a narrower range and a more realistic distribution spread than the fit under the default value.

The most recent published norovirus hospitalization rate estimates were obtained by fitting a complex statistical model to multiyear (1996–2007) HCUP data (*12*). Although the data

showed a trend of increase in hospitalization rates during 1996–2007, we cannot say whether this trend continued or not, nor can we estimate the hospitalization rate in the reference year 2014 without additional data. Instead, we assumed that the reported multiyear hospitalization rates were a random sample from 1 homogeneous population following a beta/PERT distribution. The minimum, maximum, and mode of the multiyear rates were assigned as the input parameters for the PERT distribution. The same strategy was applied to death rates (*13*) and ED visits (*14*).

Appendix 2 Figure 6 describes the modeling process for norovirus for which populations at risk of illness were scaled down to estimate burden outcomes. As in model type A and B, all model inputs are assumed to be independent and multiplicative.

Appendix 2 Figures 7 and 8 demonstrate the distributions involved in constructing estimates for norovirus including the annual illness estimate (Appendix 2 Figure 7) and the annual hospitalization estimation (Appendix 2 Figure 8). The deaths and ED visits were estimated in the same way as hospitalizations. The norovirus model estimates start with population size and incidence rate. As shown in Appendix 2 Figure 7, a mixture of 3 beta/PERT-distributions from 3 sites of the Kaiser studies was used to describe the incidence rate for illnesses. Consequently, the multimodal feature was presented in the annual illness estimate (second panel in Appendix 2 Figure 7). There was no underdiagnosis adjustment for illness, as the publication already took that into account. Although the estimated annual illness distribution was multimodal, the long tail of the water attribution rate dominated in the final output. The resulting distribution of domestic waterborne illness was right-skewed with an extended right tail. The only difference in the estimation equation between hospitalizations (Appendix 2 Figure 8), deaths, or ED visits and the illnesses was that the incidence rate consisted of only 1 beta/PERT distribution instead of a mixture of PERT distributions.

Discussion

In selecting data sources for the burden estimate, we chose an active surveillance system over a passive one when both were available, as there is a greater nonstatistical uncertainty around passive estimates. In the case of *Vibrio* estimates, we used data reported in Cholera and Other *Vibrio* Illness Surveillance (COVIS) (*15*) instead of FoodNet because most *Vibrio* cases occur in Gulf states, and FoodNet sites do not include any of those states.

Previously, 90% CrIs were reported in the foodborne burden paper (*1*,*11*). A 90% CrI adds less uncertainty in more extreme quantile distributions, is a more robust estimate, and is narrower compared with a 95% CrI. Here, we chose to report 95% CrIs to be consistent with the coverable probability (95%) commonly used in publications. In all models, we assumed that the factors are stochastically independent. In some circumstances, this assumption may not hold.

In the determination of distribution parameters for multipliers, we relied on statistics reported in previous publications or statistics calculated based on updated data. In the absence of new data, we applied the same parameter values as those used in the foodborne burden paper (*1*). When only a point estimate was available for PERT distribution (e.g., international travel rate), we estimated the range based on a 50% relative increase/decrease from the mode/point estimate on an odds scale for proportion parameters. In general, the information on underdiagnosis/underreporting for hospitalizations, deaths, and ED visits is lacking. A factor of 2 was assumed in previous publications on burden estimation (*1*,*16*). We applied the factor of 2 and expanded the range by 1 (i.e., 2 ± 1). There are alternatives of modeling uncertainty, such as using multiplicative models or/and applying different magnitudes of variability. We chose the aforementioned strategies because, overall, the approach produced reasonable estimates.

In model B, we treated the out-of-hospital death count as a constant because the variability associated with the point estimate was not available. Because the number of out-of-hospital deaths is much smaller than number of in-hospital deaths, the contribution of its uncertainty to the uncertainty of total number of deaths is negligible. During the simulation of all 4 burden outcomes, we took special measures to ensure the counts to be nonnegative by assigning zeros to negative values. Most of the simulated counts were >0, with a few exceptions that occurred in the ED visit simulation. Overall, the proportion of negative values was very small (<0.81%). Therefore, the impact of truncating a normal distribution is considered negligible.

In this study, we took a different approach for norovirus from the one employed in the foodborne disease burden study (*1*) by modeling the incidence rates, hospitalization rates, death rates, and ED rates as following a PERT distribution. The distribution parameters were extracted from statistics reported in recent publications. Despite the differences in the modeling process,

data sources, time coverage, population coverage of the data, and the nonstatistical uncertainties (*11*) compared with our estimate, the burden estimates for norovirus illnesses were comparable.

References

1. Scallan E, Hoekstra RM, Angulo FJ, Tauxe RV, Widdowson MA, Roy SL, et al. Foodborne illness acquired in the United States—major pathogens. Emerg Infect Dis. 2011;17:7–15. PubMed https://doi.org/10.3201/eid1701.P11101

2. Foodborne Diseases Active Surveillance Network (FoodNet). FoodNet 2015 surveillance report (final Data). 2017 [cited 2020 Sep 24]. https://www.cdc.gov/foodnet/reports/annual-reports-2015.html

<jrn>3. Beshearse E, Bruce BB, Nane GF, Cooke RM, Aspinall W, Hald T, et al. Using structured expert judgment for attribution of foodborne and waterborne illnesses to comprehensive transmission pathways, United States. Emerg Infect Dis. 2021 Jan [in press]. https://doi.org/10.3201/eid2701.200316

4. Vose D. Risk analysis: a quantitative guide. Chichester (England); Hoboken (NJ): John Wiley; 2008.

5. Healthcare Cost and Utilization Project (HCUP). NIS overview; 2018 [cited 2020 Sep 24]. https://hcup-us.ahrq.gov/nisoverview.jsp

6. Healthcare Cost and Utilization Project (HCUP). NEDS overview; 2018 [cited 2020 Sep 24]. https://hcup-us.ahrq.gov/nedsoverview.jsp.

7. Gargano JW, Adam EA, Collier SA, Fullerton KE, Feinman SJ, Beach MJ. Mortality from selected diseases that can be transmitted by water—United States, 2003–2009. J Water Health. 2017;15:438–50. PubMed https://doi.org/10.2166/wh.2017.301

8. National Center for Health Statistics. National Vital Statistics System—mortality statistics. 2018 [cited 2020 Sep 24]. https://www.cdc.gov/nchs/nvss/deaths.htm.

9. Hall AJ, Rosenthal M, Gregoricus N, Greene SA, Ferguson J, Henao OL, et al. Incidence of acute gastroenteritis and role of norovirus, Georgia, USA, 2004–2005. Emerg Infect Dis. 2011;17:1381–8. PubMed https://doi.org/10.3201/eid1708.101533

10. Grytdal SP, DeBess E, Lee LE, Blythe D, Ryan P, Biggs C, et al. Incidence of norovirus and other viral pathogens that cause acute gastroenteritis (AGE) among Kaiser Permanente member populations in the United States, 2012–2013. PLoS One. 2016;11:e0148395. PubMed https://doi.org/10.1371/journal.pone.0148395

11. Scallan E, Griffin PM, Angulo FJ, Tauxe RV, Hoekstra RM. Foodborne illness acquired in the United States—unspecified agents. Emerg Infect Dis. 2011;17:16–22. PubMed https://doi.org/10.3201/eid1701.P21101

12. Lopman BA, Hall AJ, Curns AT, Parashar UD. Increasing rates of gastroenteritis hospital discharges in US adults and the contribution of norovirus, 1996–2007. Clin Infect Dis. 2011;52:466–74. PubMed https://doi.org/10.1093/cid/ciq163

13. Hall AJ, Curns AT, McDonald LC, Parashar UD, Lopman BA. The roles of *Clostridium difficile* and norovirus among gastroenteritis-associated deaths in the United States, 1999–2007. Clin Infect Dis. 2012;55:216–23. PubMed https://doi.org/10.1093/cid/cis386

14. Gastañaduy PA, Hall AJ, Curns AT, Parashar UD, Lopman BA. Burden of norovirus gastroenteritis in the ambulatory setting—United States, 2001–2009. J Infect Dis. 2013;207:1058–65. PubMed https://doi.org/10.1093/infdis/jis942

15. Centers for Disease Control and Prevention. Cholera and Other *Vibrio* Illness Surveillance (COVIS); 2018 [cited 2018 Dec 4]. https://www.cdc.gov/vibrio/surveillance.html

16. Mead PS, Slutsker L, Dietz V, McCaig LF, Bresee JS, Shapiro C, et al. Food-related illness and death in the United States. Emerg Infect Dis. 1999;5:607–25. PubMed https://doi.org/10.3201/eid0505.990502

Appendix 2 Table. Model types for burden estimate of waterborne diseases, by pathogen

Model	Burden outcomes			
Pathogen	Illness	Hospitalization	Death	ED visits
Campylobacter spp.	Model type A	Model type A	Model type A	Model type B
Cryptosporidium spp.	Model type A	Model type A	Model type A	Model type B
Shiga toxin-producing *Escherichia coli* (STEC) O157	Model type A	Model type A	Model type A	Model type B
Shiga toxin-producing *E. coli* (STEC), non-O157	Model type A	Model type A	Model type A	Model type B
Giardia duodenalis	Model type A	Model type A	Model type A	Model type B
Legionella	Model type A	Model type A	Model type A	Model type B
Norovirus	Model type C	Model type C	Model type C	Model type C
Nontuberculous mycobacteria	Model type B	Model type B	Model type B	Model type B
Otitis externa	Model type B	Model type B	Model type B	Model type B
Pseudomonas pneumonia	Model type B	Model type B	Model type B	Model type B
Pseudomonas septicemia	Model type B	Model type B	Model type B	Model type B
Salmonella, nontyphoidal	Model type A	Model type A	Model type A	Model type B
Shigella spp.	Model type A	Model type A	Model type A	Model type B
Vibrio alginolyticus	Model type A	Model type A	Model type A	Model type B
Vibrio parahaemolyticus	Model type A	Model type A	Model type A	Model type B
Vibrio spp., other	Model type A	Model type A	Model type A	Model type B
Vibrio vulnificus	Model type A	Model type A	Model type A	Model type B

$$Count \times \begin{bmatrix} \textbf{Year} \\ \&/or \\ \textbf{Geo} \end{bmatrix} \times UR \times UD^{\dagger} \times \begin{bmatrix} 1 \\ or \\ H \\ or \\ D \end{bmatrix} \times [1 \quad or \quad Dom \quad or \quad W] \Rightarrow$$

Illnesses	*Domestic Illnesses*	*Domestic Waterborne Illnesses*
Hospitalizations	*Domestic Hospitalizations*	*Domestic Waterborne Hospitalizations*
Deaths	*Domestic Deaths*	*Domestic Waterborne Deaths*

Underdiagnosis (UD) for Illnesses

$$\left\{ \begin{matrix} CS(Severe) \times SS(Severe) \times P(S) \\ + \\ CS(Mild) \times SS(Mild) \times (1 - P(S)) \end{matrix} \right\} \times LT \times LS$$

Appendix 2 Figure 1. Schematic illustration of model type A, which scales case counts up, and is based on surveillance data (*11*). *Count* refers to data in the form of cases of reported illnesses. *Year* is a deterministic factor to standardize non-2014 counts to 2014 (applied as needed). *Geo* is a deterministic expansive factor to scale FoodNet counts up to the entire US population (applied as needed). *UR* is an expansive factor to scale passive surveillance case counts up to active surveillance counts to account for underreporting (applied as needed). *UD* is an expansive factor to scale laboratory-confirmed cases to illnesses not being reported to the surveillance system to account for underdiagnosis. †*UR and UD*: for hospitalization or death or ED visits, there was only 1 factor accounting for both underreporting and underdiagnosis. This multiplier follows PERT distribution with mode 2. *CS* is an expansive factor to scale care seekers up to all ill, with severe and mild illness versions. It is the reciprocal of the proportion of cases seeking care. *SS* is an expansive factor to scale submitted samples up to all ill visits, with severe and mild illness versions. It is the reciprocal of the proportion of specimen submitted for laboratory testing. *P(S)* is the proportion of actual illness that is severe. *LT* is an expansive factor to scale tests performed up to samples submitted. It is the reciprocal of the proportion of specimen being tested. *LS* is an expansive factor to scale positive tests up to true positive specimens. It is the reciprocal of sensitivity. *H* is a contractive factor to scale illnesses down to hospitalized illnesses. *D* is a contractive factor to scale illnesses down to deaths. *Dom* is a contractive factor to scale total counts down to counts that are domestically acquired (applied as needed). *W* is a contractive factor to scale overall counts down to counts that are waterborne.

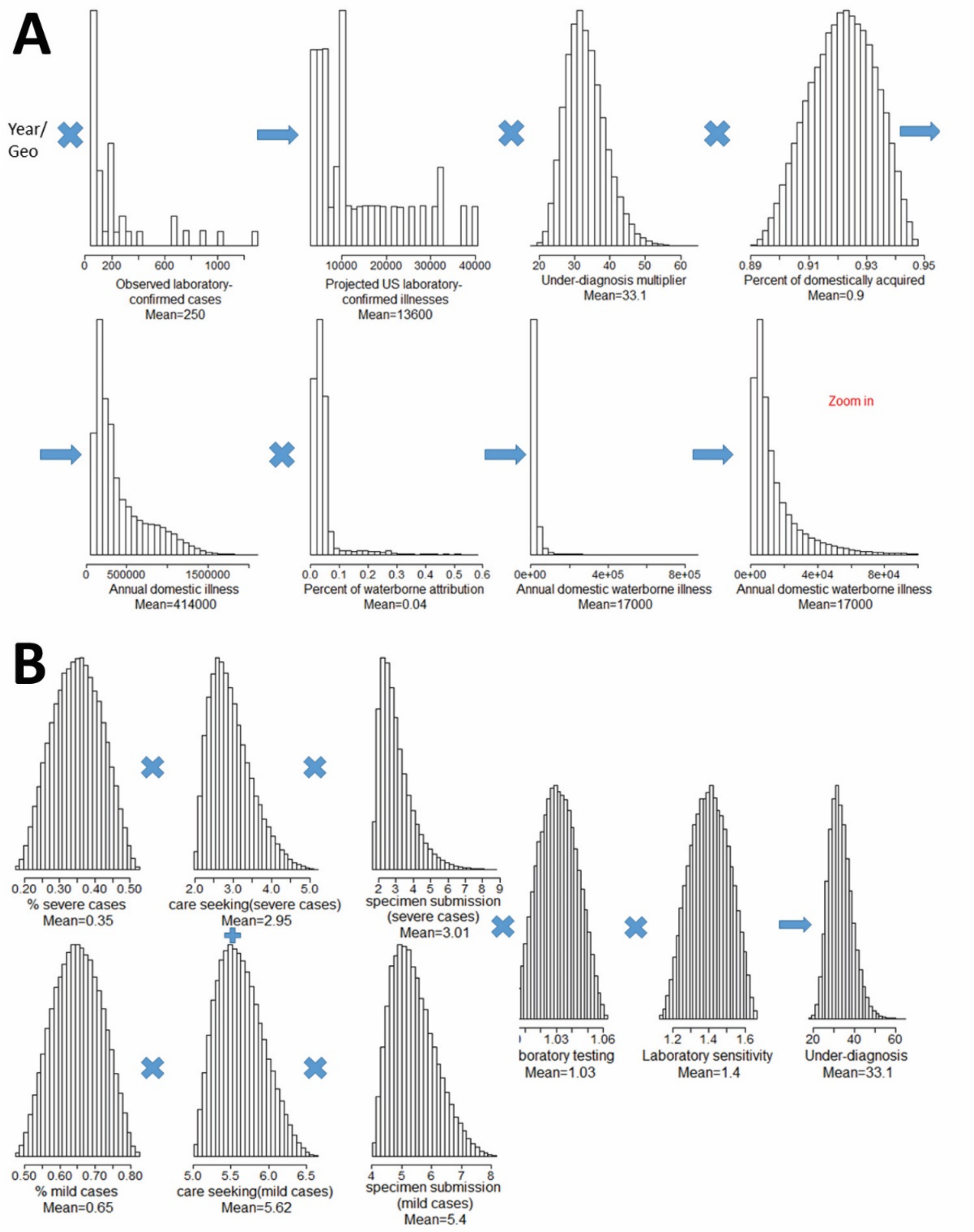

Appendix 2 Figure 2. A) Schematic diagram of the estimation of annual illnesses for *Shigella. X* axes show the relative frequency of observed or simulated values for each input or multiplier. *Year* is a deterministic factor to standardize non-2014 counts to 2014 (applied as needed). *Geo* is a deterministic expansive factor to scale FoodNet counts up to the entire U.S. population (applied as needed). B) Schematic diagram of underdiagnosis of illnesses for *Shigella. X* axes show the relative frequency of observed or simulated values for each input or multiplier.

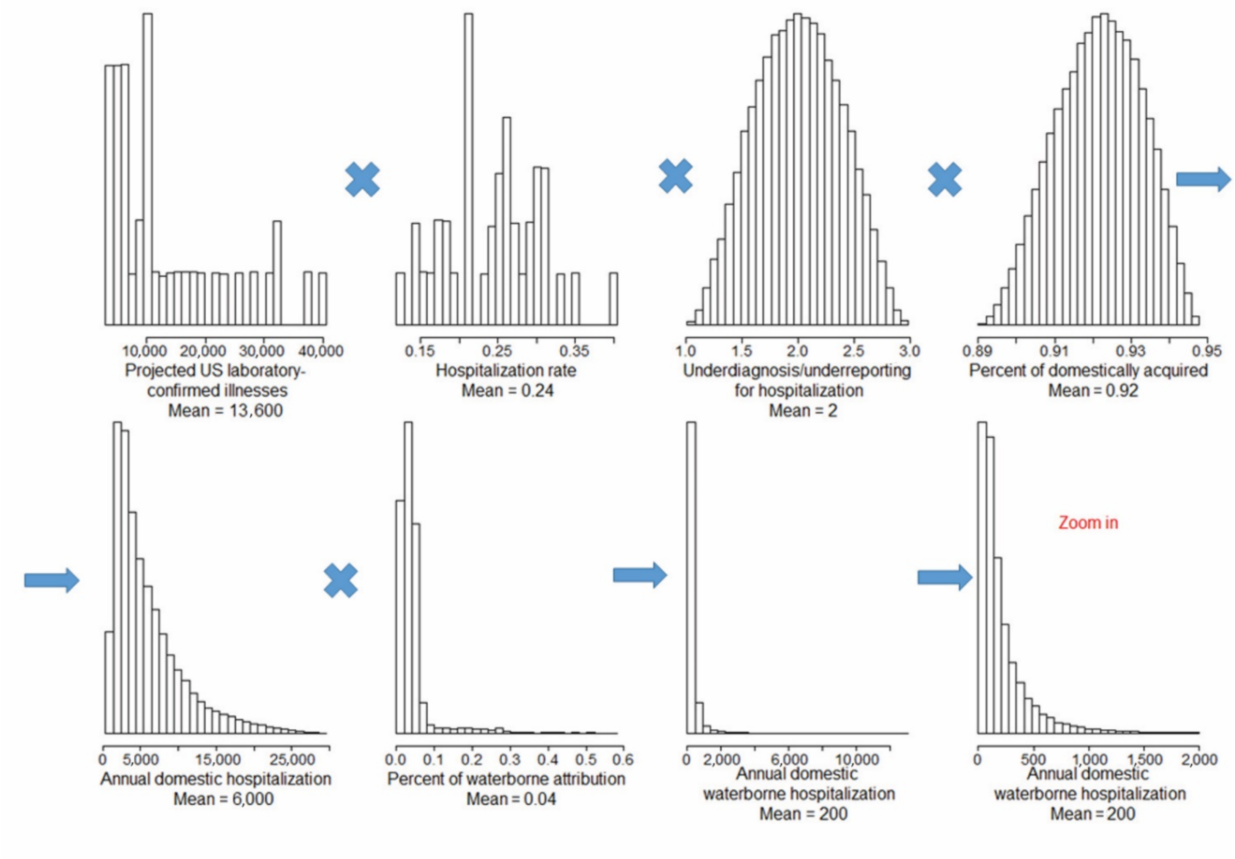

Appendix 2 Figure 3. Schematic diagram of the estimation of hospitalizations for *Shigella. X* axes show the relative frequency of observed or simulated values for each input or multiplier.

$$\begin{bmatrix} Hospitalization\ count \\ Hospitalization\ count \\ Death\ count \\ ED\ visit\ count \end{bmatrix} \times Year \times \begin{bmatrix} \frac{1}{Hospitalization\ rate} \\ 1 \\ 1 \\ 1 \end{bmatrix} \times UR \times UD \times [1 \quad or \quad Dom \quad or \quad W] \Rightarrow$$

Illness	*Domestic Illness*	*Domestic waterborne illness*
Hospitalization	*Domestic hospitalization*	*Domestic waterborne hospitalization*
Death	*Domestic death*	*Domestic waterborne death*
ED visits	*Domestic ED visits*	*Domestic waterborne ED visits*

Appendix 2 Figure 4. Schematic illustration of model type B, which scales hospitalization counts up, and is based on administrative data. *Hospitalization count, death counts,* and *ED visit counts* refer to counts reported in HCUP NIS or HCUP NEDS datasets. *Year* is a deterministic factor to standardize non-2014 counts to 2014 (applied as needed). *1/Hospitalization rate* is an expansive factor to scale the hospitalization count up to the illness count. *UR* is an expansive factor to scale passive surveillance case counts up to active surveillance counts to account for underreporting (applied as needed). *UD* is an expansive factor to scale laboratory-confirmed cases to illnesses not being reported to the surveillance system to account for underdiagnosis. *UR and UD*: for hospitalization or death or ED visits, only 1 factor accounted for both underreporting and underdiagnosis. This multiplier follows PERT distribution with mode 2. *Dom* is a contractive factor to scale total counts down to counts that are domestically acquired (applied as needed). *W* is a contractive factor to scale overall counts down to counts that are waterborne.

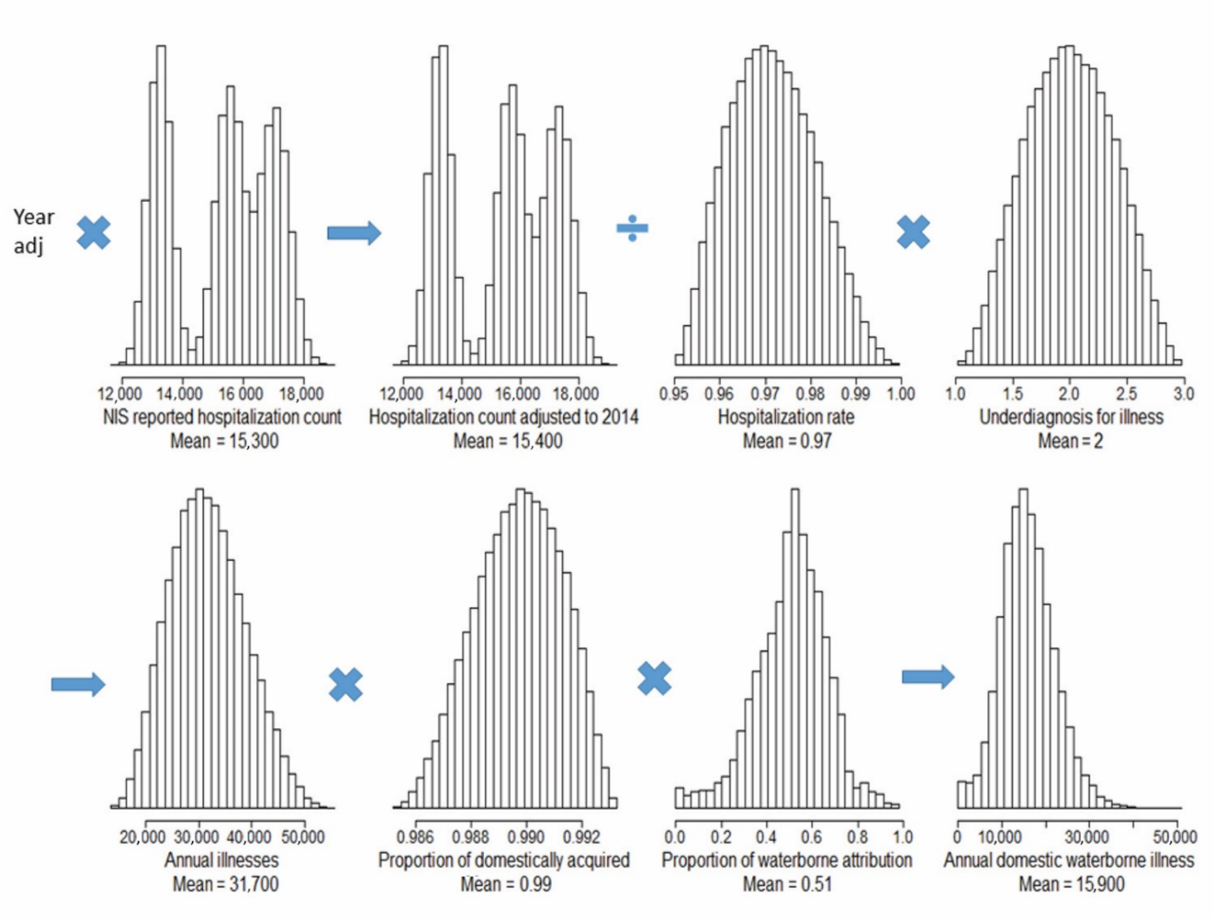

Appendix 2 Figure 5. Schematic diagram of the estimation of annual illness for *Pseudomonas* pneumonia. *X* axes show the relative frequency of observed or simulated values for each input or multiplier. *Year adj* is a deterministic factor to standardize non-2014 counts to 2014 (applied as needed).

$$\textit{US Population 2014} \times \begin{bmatrix} \textit{Illness incidence rate} \\ \textit{Hospitalization incidence rate} \\ \textit{Death incidence rate} \\ \textit{ED visit incidence rate} \end{bmatrix} \times [1 \quad or \quad Dom \quad or \quad W] \Rightarrow$$

Illness	*Domestic Illness*	*Domestic waterborne illness*
Hospitalization	*Domestic hospitalization*	*Domestic waterborne hospitalization*
Death	*Domestic death*	*Domestic waterborne death*
ED visits	*Domestic ED visits*	*Domestic waterborne ED visits*

Appendix 2 Figure 6. Schematic illustration of model type C, which scales the population at risk down, and is based on literature reported summary statistics. *Illness incidence rate:* the proportion of ill persons relative to the whole population at risk. *Hospitalization incidence rate and Death incidence rate* are the proportion of patients who were hospitalized or died relative to the whole population at risk. *ED visit incidence rate* is the proportion of patients who had ED visits (including both treated-and-released and admitted) relative to the whole population at risk. *Dom* is a contractive factor to scale total counts down to counts that are domestically acquired (applied as needed). *W* is a contractive factor to scale overall counts down to counts that are waterborne.

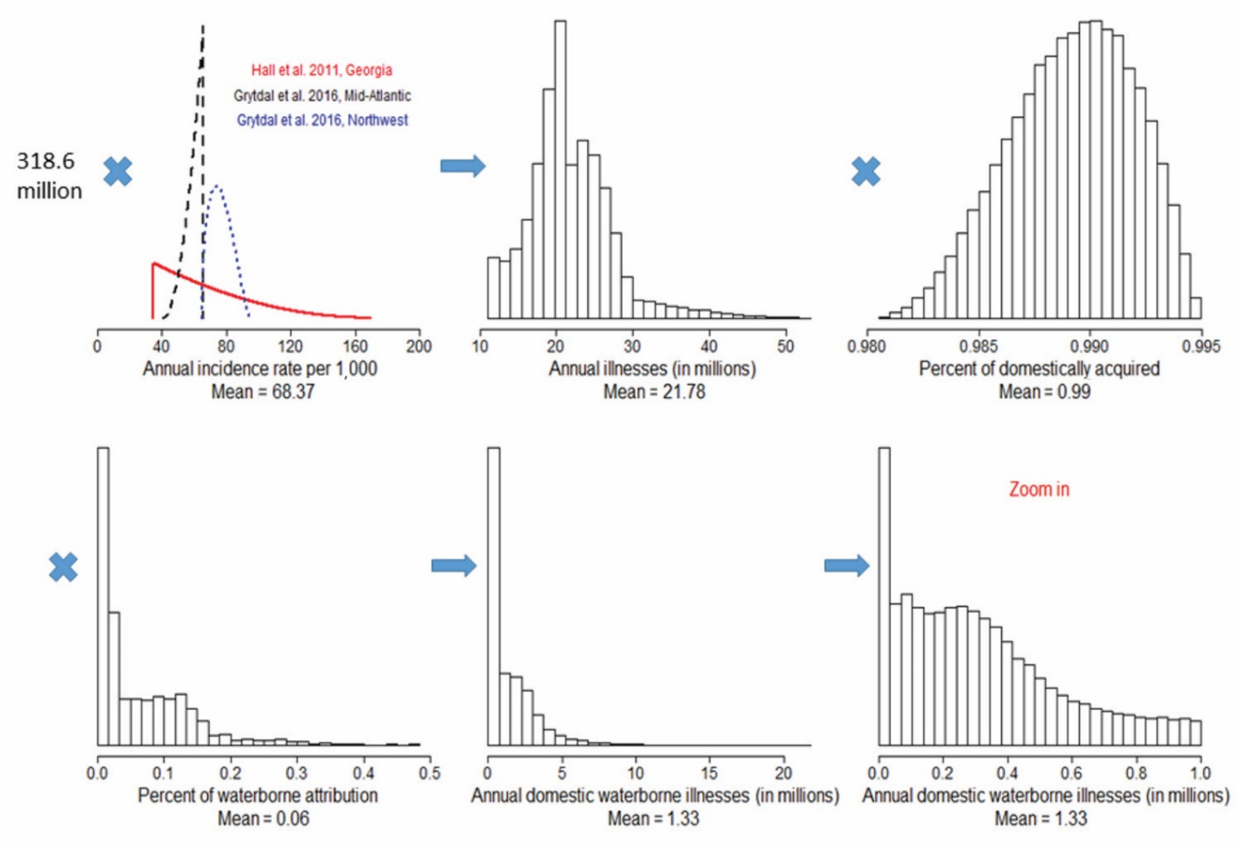

Appendix 2 Figure 7. Schematic diagram of the estimation of annual illnesses for norovirus. *X* axes show the relative frequency of observed or simulated values for each input or multiplier.

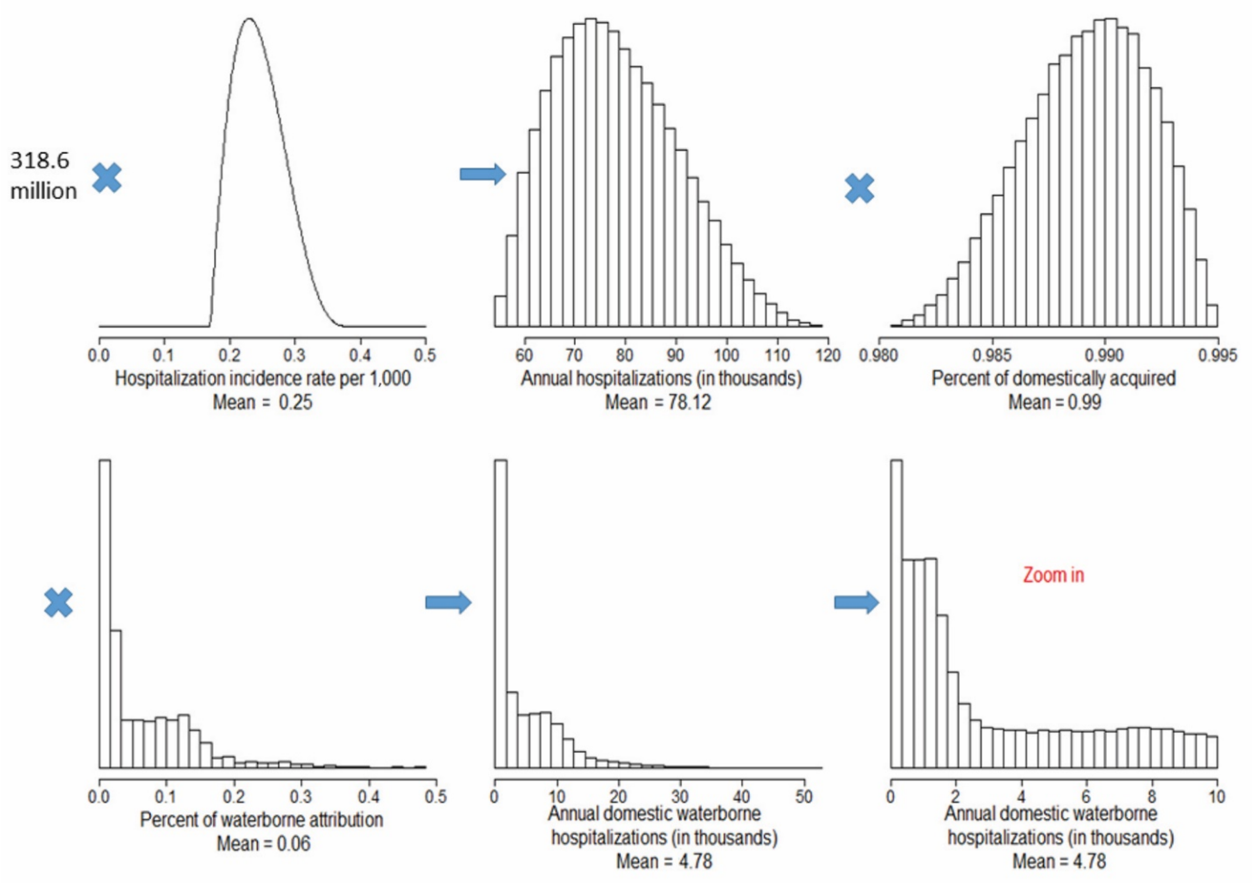

Appendix 2 Figure 8. Schematic diagram of the estimation of hospitalizations for norovirus. *X* axes show the relative frequency of observed or simulated values for each input or multiplier.
