# Supplementary material for: Estimate of Burden and Direct Healthcare Cost of Infectious Waterborne Disease in the United States
Source: Emerg Infect Dis. 2021 Jan;27(1):140–9. doi: 10.3201/eid2701.190676 (PMC7774540; doi:10.3201/eid2701.190676)
Supplement: Appendix 3 — Additional information on the burden and direct healthcare cost of infectious waterborne disease in the United States. [file 19-0676-Techapp-s3.pdf]

Article DOI: https://doi.org/10.3201/eid2701.190676

Estimate of Burden and Direct Healthcare Cost of Infectious Waterborne Disease in the United States

Appendix 3

Additional Tables

Appendix 3 Table 1. Total annual cost to commercial insurers (sum of insurer payments, in 2014 US dollars) of emergency department visits and hospitalizations from waterborne transmission of selected infections, 2014, United States

	Treat-and-release ED visit			Hospitalization			Total
Disease	Mean insurer payment per visit (95% CrI)	Annual number of commercial visits paid due to waterborne transmission (95% CrI)	Total insurer cost of treat-and-release ED visits because of waterborne disease (95% CrI)	Mean insurer payment per stay (95% CrI)	Total annual number of stays because of waterborne transmission (95% CrI)	Total insurer cost of hospitalizations for waterborne disease (95% CrI)	Total annual insurer cost for waterborne disease (95% CrI)
Campylobacteriosis	2,120 (21–8,730)	190 (19–577)	402,000 (2,190–2,200,000)	14,100 (1,250–47,100)	944 (84–3,030)	13,000,000 (354,000–57,400,000)	13,400,000 (589,000–57,800,000)
Cryptosporidiosis	1,910 (16–9,280)	310 (105–603)	593,000 (5,050–2,800,000)	16,900 (1,560–82,700)	522 (48–1,650)	8,830,000 (217,000–47,000,000)	9,420,000 (588,000–47,500,000)
Giardiasis	1,800 (43–9,280)	365 (119–722)	656,000 (10,100–3,210,000)	24,100 (1,320–167,000)	503 (167–999)	12,100,000 (553,000–81,000,000)	12,800,000 (979,000–81,300,000)
Legionnaires' disease	1,230 (79–2,960)	275 (120–495)	338,000 (20,700–991,000)	44,900 (1,500–293,000)	4,610 (3,110–5,590)	207,000,000 (7,280,000–1,340,000,000)	208,000,000 (7,640,000–1,340,000,000)
Nontuberculous mycobacterial (NTM) infection	1,480 (34–6,460)	1,460 (739–2,240)	2,170,000 (40,600–9,380,000)	43,600 (1,320–243,000)	13,500 (7,030–19,400)	587,000,000 (16,400,000–3,340,000,000)	589,000,000 (18,900,000–3,340,000,000)
Otitis externa	517 (12–2,030)	324,000 (193,000–470,000)	167,000,000 (3,660,000–682,000,000)	13,100 (1,300–57,600)	8,750 (5,230–12,700)	114,000,000 (9,700,000–522,000,000)	282,000,000 (44,500,000–994,000,000)
Pseudomonas pneumonia	1,710 (34–8,890)	81 (21–153)	138,000 (2,240–819,000)	44,400 (1,300–192,000)	2,980 (795–5,420)	132,000,000 (2,750,000–640,000,000)	132,000,000 (2,870,000–640,000,000)
Pseudomonas septicemia	2,590 (161–11,800)	9 (1–26)	23,300 (228–169,000)	62,900 (1,330–385,000)	1,020 (131–2,540)	64,000,000 (732,000–397,000,000)	64,000,000 (742,000–397,000,000)
Salmonellosis, nontyphoidal	1,520 (56–7,900)	107 (8–372)	165,000 (1,540–977,000)	16,200 (1,360–74,100)	661 (44–2,470)	10,600,000 (173,000–59,100,000)	10,700,000 (276,000–59,300,000)
Shiga toxin-producing *E. coli* infection, O157	1,570 (3–5,390)*	5 (1–14)	7,870 (15–37,300)	24,800 (1,700–148,000)	69 (7–250)	1,720,000 (36,600–11,400,000)	1,730,000 (43,500–11,400,000)

Disease	Treat-and-release ED visit: Mean insurer payment per visit (95% CrI)	Treat-and-release ED visit: Annual number of commercial visits paid due to waterborne transmission (95% CrI)	Treat-and-release ED visit: Total insurer cost of treat-and-release ED visits because of waterborne disease (95% CrI)	Hospitalization: Mean insurer payment per stay (95% CrI)	Hospitalization: Total annual number of stays because of waterborne transmission (95% CrI)	Hospitalization: Total insurer cost of hospitalizations for waterborne disease (95% CrI)	Total: Total annual insurer cost for waterborne disease (95% CrI)
Shiga toxin-producing *E. coli* infection, non-O157	1,570 (3–5,390)*	2 (0–06)	2,650 (00–15,600)	22,900 (1,140–95,700)	19 (0–77)	431,000 (00–2,990,000)	433,000 (644–3,000,000)
Shigellosis	1,960 (09–12,400)	24 (2–115)	46,600 (124–289,000)	17,700 (1,220–84,100)	113 (6–524)	1,920,000 (31,100–12,100,000)	1,960,000 (50,900–12,100,000)
Vibrio spp. infection	1,280 (300–4,440)	51 (10–112)	65,300 (4,150–262,000)	16,400 (399–49,200)	110 (67–158)	1,790,000 (37,300–6,290,000)	1,860,000 (134,000–6,380,000)
Total cost			172,000,000 (7,840,000–686,000,000)			1,160,000,000 (268,000,000–4,210,000,000)	1,330,000,000 (361,000,000–4,440,000,000)

*For emergency department (ED) visits only, costs for STEC O157 and STEC non-O157 were combined and payer proportion was derived from all ED visits instead of treat-and-release visits because of small sample size.

Appendix 3 Table 2. Total annual cost to Medicare (sum of Medicare payments, in 2014 US dollars) of emergency department visits and hospitalizations from waterborne transmission of selected infections, 2014, United States

	Treat-and-release ED visit			Hospitalization			Total
Disease	Mean insurer payment per visit (95% CrI)	Annual number of commercial visits paid because of waterborne transmission (95% CrI)	Total insurer cost of treat-and-release ED visits for waterborne disease (95% CrI)	Mean insurer payment per stay (95% CrI)	Total annual number of stays because of waterborne transmission (95% CrI)	Total insurer cost of hospitalizations for waterborne disease (95% CrI)	Total annual insurer cost for waterborne disease (95% CrI)
Campylobacteriosis	1,190 (92–9,290)	51 (5–155)	60,500 (959–519,000)	13,700 (404–57,400)	842 (75–2,700)	11,500,000 (154,000–61,000,000)	11,500,000 (212,000–61,000,000)
Cryptosporidiosis	4,110 (129–18,000)	58 (20–113)	238,000 (4,250–1,270,000)	16,900 (00–155,000)	339 (31–1,080)	5,750,000 (9–39,400,000)	5,990,000 (142,000–39,500,000)
Giardiasis	1,040 (51–2,640)	76 (25–150)	78,800 (2,390–274,000)	22,900 (388–118,000)	341 (113–677)	7,790,000 (186,000–38,600,000)	7,870,000 (286,000–38,600,000)
Legionnaires' disease	626 (135–2,160)	43 (19–78)	27,200 (3,160–131,000)	32,800 (1,080–181,000)	4,930 (3,330–5,980)	162,000,000 (5,750,000–908,000,000)	162,000,000 (5,770,000–908,000,000)
Nontuberculous mycobacterial (NTM) infection	1,960 (47–10,000)	2,800 (1,420–4,280)	5,490,000 (81,200–33,400,000)	26,800 (647–159,000)	28,700 (15,000–41,400)	771,000,000 (17,200,000–4,440,000,000)	777,000,000 (19,500,000–4,440,000,000)
Otitis externa	421 (34–2,500)	43,600 (25,900–63,300)	18,400,000 (1,330,000–111,000,000)	13,500 (632–63,800)	8,740 (5,230–12,700)	118,000,000 (5,130,000–605,000,000)	136,000,000 (13,700,000–644,000,000)
Pseudomonas pneumonia	411 (54–1,970)	166 (43–315)	68,200 (4,490–393,000)	26,500 (572–145,000)	10,500 (2,790–19,000)	278,000,000 (3,880,000–1,530,000,000)	278,000,000 (3,940,000–1,530,000,000)
Pseudomonas septicemia	213 (67–529)	23 (1–68)	4,920 (188–20,100)	32,800 (768–179,000)	4,000 (516–9,990)	131,000,000 (1,410,000–783,000,000)	131,000,000 (1,410,000–783,000,000)
Salmonellosis, nontyphoidal	1,470 (79–8,960)	24 (2–82)	34,700 (391–257,000)	17,100 (474–80,900)	515 (34–1,920)	8,870,000 (64,400–50,300,000)	8,900,000 (84,500–50,300,000)
Shiga toxin-producing *E. coli* infection, O157	555 (84–1,510)*	5 (1–14)	2,790 (124–13,500)	16,600 (291–88,200)	42 (4–153)	700,000 (866–4,320,000)	702,000 (2,760–4,320,000)
Shiga toxin-producing *E. coli* infection, non-O157	555 (84–1,510)*	2 (0–06)	940 (0–5,710)	33,200 (1,040–243,000)	40 (0–167)	1,320,000 (0–11,000,000)	1,320,000 (274–11,000,000)
Shigellosis	297 (139–570)	3 (0–15)	892 (49–3,900)	10,200 (194–38,200)	54 (3–252)	546,000 (2,690–3,320,000)	547,000 (3,380–3,320,000)
Vibrio spp. infection	374 (269–479)	8 (2–18)	3,090 (528–7,690)	21,600 (5,280–76,300)	106 (65–153)	2,290,000 (457,000–8,750,000)	2,290,000 (461,000–8,750,000)
Total cost			24,400,000 (2,710,000–121,000,000)			1,500,000,000 (319,000,000–5,820,000,000)	1,520,000,000 (338,000,000–5,840,000,000)

*For emergency department (ED) visits only, costs for STEC O157 and STEC non-O157 were combined and payer proportion was derived from all ED visits instead of treat-and-release visits because of small sample size.

Appendix 3 Table 3. Total annual cost to Medicaid (sum of Medicaid payments, in 2014 US dollars) of emergency department visits and hospitalizations from waterborne transmission of selected infections, 2014, United States

	Treat-and-release ED visit			Hospitalization			Total
Disease	Mean insurer payment per visit (95% CrI)	Annual number of commercial visits paid because of waterborne transmission (95% CrI)	Total insurer cost of treat-and-release ED visits for waterborne disease (95% CrI)	Mean insurer payment per stay (95% CrI)	Total annual number of stays because of waterborne transmission (95% CrI)	Total insurer cost of hospitalizations for waterborne disease (95% CrI)	Total annual insurer cost for waterborne disease (95% CrI)
Campylobacteriosis	436 (13–1,480)	77 (08–234)	33,600 (383–170,000)	5,710 (43–29,000)	363 (32–1,160)	2,050,000 (4,870–11,600,000)	2,080,000 (16,100–11,700,000)
Cryptosporidiosis	511 (1–3,420)	124 (42–241)	63,200 (161–405,000)	10,700 (16–64,200)	258 (24–817)	2,730,000 (3,300–15,300,000)	2,790,000 (29,200–15,400,000)
Giardiasis	555 (1–2,000)	126 (41–249)	69,600 (146–278,000)	14,300 (79–88,000)	254 (84–506)	3,630,000 (22,100–21,500,000)	3,700,000 (75,900–21,600,000)
Legionnaires' disease	*	111 (48–199)	65,600 (16,600–174,000)	18,600 (17–99,300)	1,260 (846–1,520)	23,300,000 (21,000–130,000,000)	23,400,000 (78,600–130,000,000)
Nontuberculous mycobacterial (NTM) infection	699 (4–3,130)	807 (407–1,230)	565,000 (3,520–2,490,000)	14,900 (45–70,300)	9,250 (4,830–13,300)	138,000,000 (361,000–687,000,000)	138,000,000 (811,000–689,000,000)
Otitis externa	194 (24–545)	200,000 (119,000–290,000)	38,700,000 (4,440,000–115,000,000)	6,530 (25–36,900)	5,740 (3,430–8,310)	37,400,000 (143,000–203,000,000)	76,200,000 (11,400,000–270,000,000)
Pseudomonas pneumonia	301 (20–1,710)	44 (11–84)	13,300 (556–77,400)	11,500 (18–53,500)	2,020 (540–3,680)	23,200,000 (26,800–115,000,000)	23,200,000 (40,200–115,000,000)
Pseudomonas septicemia	535 (51–2,470)	4 (0–12)	2,210 (25–17,100)	19,600 (46–113,000)	576 (74–1,440)	11,300,000 (12,900–72,200,000)	11,300,000 (14,300–72,200,000)
Salmonellosis, nontyphoidal	415 (17–2,090)	63 (5–218)	26,100 (302–173,000)	6,820 (32–26,300)	340 (23–1,270)	2,360,000 (4,580–13,500,000)	2,380,000 (13,300–13,500,000)
Shiga toxin-producing *E. coli* infection, O157	165 (10–672) †	2 (0–6)	368 (8–2,110)	4,270 (03–30,200)	27 (3–100)	116,000 (46–773,000)	116,000 (210–774,000)
Shiga toxin-producing *E. coli* infection, non-O157	165 (10–672) †	1 (0–3)	125 (0–832)	4,660 (41–32,000)	16 (0–64)	73,200 (0–524,000)	73,300 (28–524,000)
Shigellosis	294 (15–1,480)	37 (3–181)	10,900 (221–57,400)	7,620 (37–51,300)	78 (4–362)	611,000 (1,370–3,510,000)	622,000 (7,360–3,520,000)
Vibrio spp. infection	260 (30–1,010)	16 (3–36)	4,230 (361–15,400)	4,600 (13–46,000)	35 (22–51)	162,000 (445–1,350,000)	167,000 (1,550–1,360,000)
Total cost			39,600,000 (5,200,000–116,000,000)			245,000,000 (35,100,000–860,000,000)	284,000,000 (62,700,000–906,000,000)

*N<5, costs not reported.

†For emergency department (ED) visits only, costs for STEC O157 and STEC non-O157 were combined and payer proportion was derived from all ED visits instead of treat-and-release visits because of small sample size.

Appendix 3 Table 4. Total annual cost (in 2014 US dollars) of emergency department visits and hospitalizations from all transmission routes of selected diseases, 2014, United States

	Treat-and-release ED visit			Hospitalization			Total
Disease or syndrome	Cost per visit (95% CrI)	Total annual number of treat-and-release ED visits because of all transmission routes (95% CrI)	Total cost of treat-and-release ED visits for selected diseases (95% CrI)	Cost per stay (95% CrI)	Total annual number of stays because of all transmission routes (95% CrI)	Total cost of hospitalizations for selected diseases (95% CrI)	Total annual direct healthcare cost of selected diseases (95% CrI)
Campylobacteriosis	1,710 (137–5,810)	2900 (1620–4630)	4,950,000 (398,000–18,700,000)	13,600 (3,850–35,800)	19,300 (8790–34,900)	261,000,000 (56,800,000–790,000,000)	266,000,000 (61,500,000–794,000,000)
Cryptosporidiosis	1,960 (238–6,270)	1260 (742–1880)	2,460,000 (257,000–8,150,000)	16,100 (4,360–55,400)	2,860 (439–8,060)	45,900,000 (3,710,000–195,000,000)	48,400,000 (5,670,000–197,000,000)
Giardiasis	1,620 (196–7,510)	1460 (902–2,090)	2,360,000 (284,000–9,880,000)	21,800 (6,160–99,200)	2,830 (1760–4,070)	61,800,000 (14,300,000–265,000,000)	64,200,000 (16,200,000–267,000,000)
Legionnaires' disease	691 (288–1,390)	691 (316–1,220)	477,000 (137,000–1,160,000)	37,100 (7,950–149,000)	11,200 (8,750–13,300)	416,000,000 (84,800,000–1,740,000,000)	416,000,000 (85,400,000–1,740,000,000)
Nontuberculous mycobacterial (NTM) infection	1,610 (129–6,430)	7,150 (5110–9,620)	11,500,000 (892,000–46,100,000)	29,600 (6,350–120,000)	72,400 (57,300–89,700)	2,140,000,000 (436,000,000–8,720,000,000)	2,160,000,000 (448,000,000–8,730,000,000)
Norovirus*	1,140	429,000	491,000,000	6,080	78,100	475,000,000	966,000,000
Otitis externa	494 (120–1,430)	726,000 (466,000–994,000)	358,000,000 (79,600,000–1,070,000,000)	12,200 (3,320–42,400)	29,700 (19,200–40,600)	365,000,000 (89,900,000–1,330,000,000)	723,000,000 (250,000,000–1,990,000,000)
Pseudomonas pneumonia	856 (89–4,190)	580 (321–902)	496,000 (45,500–2,440,000)	29,300 (5,910–114,000)	30,800 (18,700–44,700)	901,000,000 (164,000,000–3,710,000,000)	901,000,000 (165,000,000–3,710,000,000)
Pseudomonas septicemia	923 (95–3,190)	164 (36–326)	151,000 (9140–731,000)	38,200 (6,340–172,000)	25,300 (16300–34,800)	968,000,000 (149,000,000–4,240,000,000)	968,000,000 (149,000,000–4,240,000,000)
Salmonellosis, nontyphoidal	1,230 (161–4,500)	3400 (2100–4900)	4,200,000 (510,000–16,000,000)	14,900 (4,300–46,900)	26,600 (11400–52,800)	395,000,000 (79,500,000–1,410,000,000)	400,000,000 (83,300,000–1,420,000,000)
Shiga toxin-producing *E. coli* infection, O157†	1,070 (109–2,350)	252 (92–465)	269,000 (16600–794,000)	19,000 (3,790–85,000)	2640 (487–7630)	50,400,000 (4,270,000–244,000,000)	50,700,000 (4,530,000–244,000,000)
Shiga toxin-producing *E. coli* infection, non-O157†	1,070 (109–2,350)	75 (12–171)	79,600 (3090–274,000)	24,200 (4,780–138,000)	1420 (264–3810)	34,500,000 (2490000–223,000,000)	34,600,000 (2,570,000–223,000,000)
Shigellosis	952 (115–3,980)	1650 (540–2860)	1,570,000 (123,000–7,420,000)	14,200 (4,130–48,000)	6380 (929–20,300)	90,700,000 (8,170,000–398,000,000)	92,300,000 (9,280,000–400,000,000)
Vibrio spp. infection	1,030 (293–3,330)	366 (122–700)	376,000 (53,700–1,270,000)	16,000 (3,780–39,900)	782 (567–1030)	12,500,000 (2,620,000–32,300,000)	12,900,000 (3,010,000–32,800,000)
Total cost			878,000,000 (596,000,000–1,590,000,000)			6,220,000,000 (2,980,000,000–15,400,000,000)	7,100,000,000 (3,770,000,000–16,300,000,000)

*For norovirus only, costs were derived from previously published estimates that did not include uncertainty intervals. In addition, the number of emergency department visits includes visits in which the patient was admitted to the hospital.

†For emergency department (ED) visits only, costs for STEC O157 and STEC non-O157 were combined and payer proportion was derived from all ED visits instead of treat-and-release visits because of small sample size.
